# An ecological measure of immune-cancer colocalization as a prognostic factor for breast cancer

**DOI:** 10.1186/s13058-015-0638-4

**Published:** 2015-09-22

**Authors:** Carlo C. Maley, Konrad Koelble, Rachael Natrajan, Athena Aktipis, Yinyin Yuan

**Affiliations:** Centre for Evolution and Cancer, The Institute of Cancer Research, London, UK; Center for Evolution and Cancer, University of California San Francisco, San Francisco, CA USA; Biodesign Institute, School of Life Sciences, Arizona State University, Tempe, AZ USA; Division of Breast Cancer, The Institute of Cancer Research, London, UK; Department of Histopathology, The Royal Marsden Hospital, London, UK; Division of Molecular Pathology, The Institute of Cancer Research, London, UK; Department of Psychology, Arizona State University, Tempe, AZ USA; Center for Evolution and Medicine, Biodesign Institute, Arizona State University, AZ, USA; Centre for Molecular Pathology, The Royal Marsden Hospital, London, UK

## Abstract

**Introduction:**

Abundance of immune cells has been shown to have prognostic and predictive significance in many tumor types. Beyond abundance, the spatial organization of immune cells in relation to cancer cells may also have significant functional and clinical implications. However there is a lack of systematic methods to quantify spatial associations between immune and cancer cells.

**Methods:**

We applied ecological measures of species interactions to digital pathology images for investigating the spatial associations of immune and cancer cells in breast cancer. We used the Morisita-Horn similarity index, an ecological measure of community structure and predator–prey interactions, to quantify the extent to which cancer cells and immune cells colocalize in whole-tumor histology sections. We related this index to disease-specific survival of 486 women with breast cancer and validated our findings in a set of 516 patients from different hospitals.

**Results:**

Colocalization of immune cells with cancer cells was significantly associated with a disease-specific survival benefit for all breast cancers combined. In HER2-positive subtypes, the prognostic value of immune-cancer cell colocalization was highly significant and exceeded those of known clinical variables. Furthermore, colocalization was a significant predictive factor for long-term outcome following chemotherapy and radiotherapy in HER2 and Luminal A subtypes, independent of and stronger than all known clinical variables.

**Conclusions:**

Our study demonstrates how ecological methods applied to the tumor microenvironment using routine histology can provide reproducible, quantitative biomarkers for identifying high-risk breast cancer patients. We found that the clinical value of immune-cancer interaction patterns is highly subtype-specific but substantial and independent to known clinicopathologic variables that mostly focused on cancer itself. Our approach can be developed into computer-assisted prediction based on histology samples that are already routinely collected.

**Electronic supplementary material:**

The online version of this article (doi:10.1186/s13058-015-0638-4) contains supplementary material, which is available to authorized users.

## Introduction

In recent years, the recognition that cancer is an evolutionary process has penetrated much of cancer biology and evolutionary biology [1, 2], and a variety of evolutionary approaches have been adapted for use in cancer biology (e.g., diversity measures for predicting progression) [3–7]. However, cancer is more than just an evolutionary process; it is also an ecological process [8]. Cancer cells utilize resources and construct habitats within the tissues of the body just as organisms do in the natural world. The ecology of cancer is therefore critical for our understanding of the natural forces that shape cancer development, yet has rarely been systematically investigated in tumors. This parallel between organismal ecology and the tumor microenvironment means that there are unrealized opportunities for adopting measures from ecology for understanding the dynamics and selective pressures on a tumor, which may lead to improved cancer prognosis, prediction, risk stratification and therapeutics.

Immune cell infiltration is one of the most important aspects of the ecology of neoplastic cells [9–11]. An array of studies has shown that the spatial location of immune cells relative to cancer cells is clinically important in many different cancer types [9, 12–15]. However, the spatial patterns of associations between cancer cells and immune cells are rarely quantified, and the evolutionary and ecological processes underlying the role of immune infiltration in human tumors are poorly understood. Recently, we discovered that dense concentrations (“hotspots”) formed by both immune and cancer cells, rather than those formed by one cell type alone, are associated with good prognosis in estrogen receptor (ER)-negative breast cancer [16]. This highlights the importance of investigating how cancer and immune cells are spatially related and raises the question: can we characterize the spatial association between cancer and immune cells and thereby elucidate the ecological dynamics that could ultimately influence tumor progression and response to treatment?

Evolution and ecology can provide a framework for understanding these complex dynamics and predicting clinical outcomes [2]. The Morisita-Horn index [17], a measure that has been used to study community structure in ecology [18, 19], can be adapted for quantifying spatial colocalization of immune and cancer cells. The relationship between colocalization (as measured by the Morisita-Horn index) and patient survival can further reveal whether the immune cells are having a pro- or anti-tumor effect and so provide information about the types of ecological interaction occurring in the tumor. Thus, the aims of this study were 1) to use ecological measures for quantifying spatial associations between cancer cells and immune cells, 2) perform a comprehensive evaluation of the spatial parameters involved in that quantification and 3) to investigate the implications of immune cell colocalization with cancer cells on cancer progression across all subtypes of breast cancer in a large patient cohort.

## Methods

### Clinical samples and histology analysis

Hematoxylin and eosin (H&E)-stained section images representing 1002 consenting patients in the METABRIC study, under ethical approval by relevant review boards as reported in our previous publication [20], were analyzed using our tool CRImage [21]. More details on the sample set can be found in Additional file 1. The principle of our image analysis tool is to identify immune cells on H&E images based on their typical morphology of small, round and homogeneously basophilic nuclei, which differentiates them from other leukocytes such as neutrophils with more pleomorphic nuclei [2, 3, 12]. Cancer cells typically have nuclei of large size and greater variability in texture and shape. They can be differentiated from the generally more elongated nuclei of fibroblasts and endothelial cells. H&E images have previously been used for evaluating immune infiltration in breast cancer [12, 14, 22, 23]. We have performed three experiments to test the accuracy of our image analysis tool: 1) comparison with visual scoring of tumor cellularity and immune infiltrate (Additional file 2: Figure S1, Jonckheere-Terpstra (JT) trend test *p* <0.0001); 2) 10-fold cross-validation within the training set (90.1 % accuracy) [21]; 3) 10,000 single-cell annotation by an expert pathologist in random samples (correlation *R*^2^ = 0.98; specifically for lymphocyte *R*^2^ = 0.99) [21]. On average three sections from different tumor locations were obtained for each tumor to increase our ability to capture intra-tumor spatial heterogeneity. The patients were split into a discovery cohort (486 samples, 475 with survival data) and a validation cohort (516 samples, 514 with survival data), thus obtaining two cohorts of similar size. Information on chemotherapy (CT), radiotherapy (RT) and hormone therapy (HT) was available for all patients.

### Immune infiltrate scores

Visual scores for immune abundance is available for 675 samples provided by three pathologists in the METABRIC consortium in three categories: absent, mild and severe. The automated score for immune abundance is defined as the percentage of immune cells identified by image analysis, and was highly correlated with the visual score (*p* <0.0001, JT trend test). We used a cutoff of 8 % as reported in our previous study for estrogen receptor-negative (ER−) cancer [21]. An optimal cutoff search did not yield a more significant result for survival analysis in human epidermal growth factor receptor 2-positive (Her2+) tumors. The visual score for immune-cancer cell colocalization was obtained semiquantitatively for 40 randomly selected cases on the same digital images, which were subjected to automated image analysis by a pathologist blinded to the results of the automated scoring, using a four-tiered scale comprising the following intratumoral distribution patterns (modified after the previous publication [24]): focal (disperse circumscribed immune cell aggregates), low (mild immune cell infiltrate irregularly distributed and spatially unassociated with the invasive tumor epithelia), moderate (immune cell infiltrate displaying some spatial association with the invasive tumor epithelia) and marked (dense immune cell infiltrate closely associated with the invasive tumor epithelia).

### Other statistical methods

Monotone trend between a continuous variable and a categorical variable was tested using the JT trend test [25]. Association with clinicopathologic variables was tested using the Kruskal-Wallis test [26] or Fisher’s test in the discovery and validation cohorts. Survival analysis was performed with breast cancer-specific 10-year survival data. The Kaplan-Meier estimator was used for patient stratification and the log-rank test was used for testing for differences among groups. The Cox proportional hazards regression model was fitted to the survival data, and hazard ratios (HR) and 95 % confidence intervals were computed to determine the correlation with disease-specific survival, where the log-rank test with *p* <0.05 was considered significant. Optimal cutoff searching for dichotomizing the proposed indices was carried out by searching stepwise in the discovery cohort from 20 to 80 percentiles at an interval of 1.5. The cutoffs that displayed the highest prognostic significance with the log-rank test were selected and used for the validation cohort. Tests using a decreasing amount of tissue were performed by dividing slides into areas containing 75 %, 50 % and 25 % of square polygons that were used to compute the Morisita index (Additional file 1). R code and data for reproducing all our results are provided (Additional file 3: Sweave file).

## Results

### Immune-cancer cell colocalization was independent of known parameters of breast cancer

**Fig. 1:**
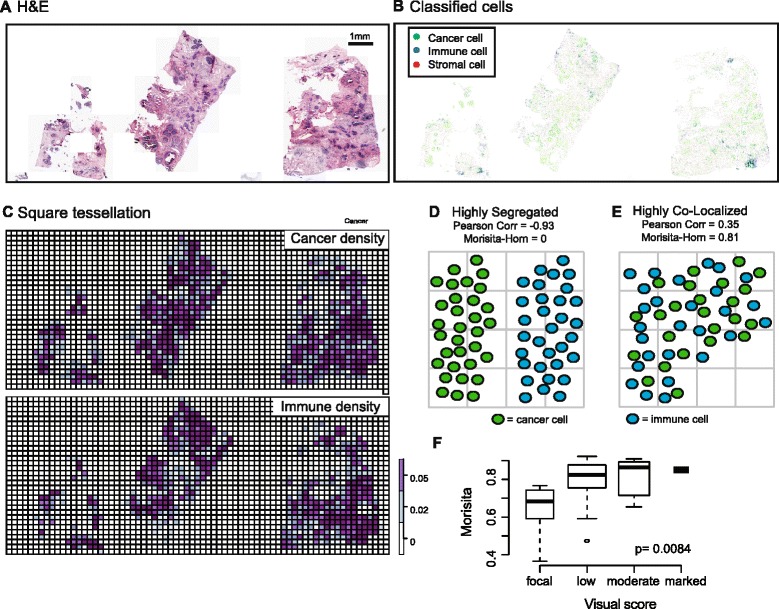
Measuring spatial colocalization of immune and cancer cells through image analysis and spatial statistics. **a** Example H&E image of a breast tumor. Three sections obtained from different locations of the tumor were stained with H&E. **b** Automated image analysis was used to identify cell types (cancer, immune and stromal cells including fibroblasts and endothelial cells) in this image. **c** Density of cancer cells and immune cells per square after applying a square tessellation to this image; squares with less than a predefined amount of tissue would be excluded from analysis. **d** and **e** Schematic over an arbitrary spatial plane demonstrating how colocalization statistics can discriminate a highly segregated cell pattern from a highly colocalized cell pattern. **f** Significant correlation between the Morisita index and visual scoring for immune-cancer colocalization in 40 randomly selected samples (JT-test *p* = 0.0084); focal (disperse immune cell aggregates), low (mild infiltrate unassociated with cancer), moderate (some spatial association with cancer) and marked (dense infiltrate closely associated with cancer)

H&E-stained tumor section images representing 1002 primary breast tumors were analyzed using our image analysis tool to identify cancer cells and immune cells based on their morphology (Fig. 1a-b, Table 1, Additional file 1). To study the spatial distribution of cancer cells and immune cells, each H&E image was virtually divided into non-overlapping squares of 250 μm × 250 μm based on the effective cell − cell communication distance [27]. The number of cancer cells and immune cells within each square was counted (Fig. 1c). To calculate colocalization using cell counts, we then applied two statistics to the data: Morisita-Horn’s similarity index and Pearson correlation (Additional file 1). The value of the Morisita-Horn similarity index ranges from 0, indicating no similarity between two community structures, to 1, when the two structures are the same (an equal number of immune cells and cancer cells in each tessellation square), indicating that the two species are highly colocalized (Fig. 1d and e). We observed a good correlation between visual scoring by an expert pathologist and image analysis scores for immune-cancer colocalization in a subset of samples that were randomly selected (*p* = 0.008, Fig. 1f, “Methods”). Furthermore, the Morisita index was not correlated with clinical and molecular variables including grade, node, size, ER and Her2 status or PAM50 gene set expression subtypes [28] (*p* >0.05 in at least one cohort).

**Table 1:** Distribution of the clinicopathological characteristics of all samples or Her2-amplified samples split into discovery and validation cohorts according to the Morisita index

Characteristics		All subtypes (discovery)			All subtypes (validation)			Her2+ (discovery)			HER2+ (validation)		
		Morisita high	Morisita low	*P*	Morisita high	Morisita low	*P*	Morisita high	Morisita low	*P*	Morisita high	Morisita low	*P*
Number		316	170		377	139		42	34		37	20	
Follow up (months)		117.7 (4.9–120)	110.3 (4.2–120)		64.8 (0.3–120)	50.5 (1.9–120)		108.1 (21.2–120)	61.5 (6.8–120)		62.3 (1.8–120)	36.3 (10–88.4)	
Age, years		59.8 (27.6–86.1)	59.3 (21.9–83.4)	0.38	60.8 (30–92.1)	60.9 (26.4–96.3)	0.81	57 (28.4–80.6)	54.2 (21.9–71)	0.23	57.4 (33.6–87)	55.9 (35.9–87.2)	0.65
Death	No	246 (77.8 %)	113 (66.5 %)	0.0074*	334 (88.6 %)	115 (82.7 %)	0.1	25 (59.5 %)	14 (41.2 %)	0.097	35 (94.6 %)	13 (65 %)	0.0063*
	Yes	64 (20.3 %)	53 (31.2 %)		43 (11.4 %)	24 (17.3 %)		14 (33.3 %)	19 (55.9 %)		2 (5.4 %)	7 (35 %)	
Size	<2 cm	122 (38.6 %)	66 (38.8 %)	0.88	120 (31.8 %)	41 (29.5 %)	0.27	9 (21.4 %)	13 (38.2 %)	0.15	9 (24.3 %)	9 (45 %)	0.056
	>2 cm, <5 cm	177 (56 %)	97 (57.1 %)		226 (59.9 %)	80 (57.6 %)		30 (71.4 %)	17 (50 %)		24 (64.9 %)	7 (35 %)	
	>5 cm	17 (5.4 %)	7 (4.1 %)		29 (7.7 %)	17 (12.2 %)		3 (7.1 %)	4 (11.8 %)		3 (8.1 %)	4 (20 %)	
Node	Neg (pN0)	153 (48.4 %)	84 (49.4 %)	0.85	180 (47.7 %)	57 (41 %)	0.19	19 (45.2 %)	10 (29.4 %)	0.23	15 (40.5 %)	9 (45 %)	0.78
	Pos (pN1-pN3)	163 (51.6 %)	86 (50.6 %)		193 (51.2 %)	80 (57.6 %)		23 (54.8 %)	24 (70.6 %)		22 (59.5 %)	11 (55 %)	
Grade	1	37 (11.7 %)	18 (10.6 %)	0.39	42 (11.1 %)	9 (6.5 %)	0.069	1 (2.4 %)	0 (0 %)	1	2 (5.4 %)	0 (0 %)	0.86
	2	120 (38 %)	56 (32.9 %)		123 (32.6 %)	59 (42.4 %)		8 (19 %)	7 (20.6 %)		6 (16.2 %)	3 (15 %)	
	3	149 (47.2 %)	92 (54.1 %)		192 (50.9 %)	64 (46 %)		32 (76.2 %)	27 (79.4 %)		26 (70.3 %)	15 (75 %)	
ER	Neg	76 (24.1 %)	54 (31.8 %)	0.069	82 (21.8 %)	39 (28.1 %)	0.16	22 (52.4 %)	19 (55.9 %)	0.82	14 (37.8 %)	14 (70 %)	0.028*
	Pos	240 (75.9 %)	116 (68.2 %)		295 (78.2 %)	100 (71.9 %)		20 (47.6 %)	15 (44.1 %)		23 (62.2 %)	6 (30 %)	
Her2	Not amplified	271 (85.8 %)	139 (81.8 %)	0.29	335 (88.9 %)	123 (88.5 %)	0.87	42 (100 %)	34 (100 %)	NA	37 (100 %)	20 (100 %)	NA
	Amplified	45 (14.2 %)	31 (18.2 %)		41 (10.9 %)	16 (11.5 %)		20 (47.6 %)	14 (41.2 %)		24 (64.9 %)	6 (30 %)	
*TP53*	WT	224 (70.9 %)	110 (64.7 %)	0.076	252 (66.8 %)	94 (67.6 %)	1	20 (47.6 %)	20 (58.8 %)	0.49	7 (18.9 %)	10 (50 %)	0.011*
	Mutated	83 (26.3 %)	59 (34.7 %)		94 (24.9 %)	35 (25.2 %)		9 (21.4 %)	28 (82.4 %)		7 (18.9 %)	14 (70 %)	
IA	Low	89 (28.2 %)	148 (87.1 %)	1.1×10^−37^*	71 (18.8 %)	111 (79.9 %)	1.5×10^−37^*	33 (78.6 %)	6 (17.6 %)	1.6×10^−7^*	30 (81.1 %)	6 (30 %)	0.00036*
	High	227 (71.8 %)	22 (12.9 %)		306 (81.2 %)	28 (20.1 %)		4 (9.5 %)	4 (11.8 %)		5 (13.5 %)	2 (10 %)	
Pam50	Basal	54 (17.1 %)	43 (25.3 %)	0.15	57 (15.1 %)	21 (15.1 %)	0.45	25 (59.5 %)	16 (47.1 %)	0.83	13 (35.1 %)	9 (45 %)	0.46
	HER2	50 (15.8 %)	18 (10.6 %)		30 (8 %)	12 (8.6 %)		5 (11.9 %)	4 (11.8 %)		7 (18.9 %)	1 (5 %)	
	LumA	106 (33.5 %)	49 (28.8 %)		129 (34.2 %)	50 (36 %)		6 (14.3 %)	7 (20.6 %)		9 (24.3 %)	4 (20 %)	
	LumB	86 (27.2 %)	48 (28.2 %)		103 (27.3 %)	28 (20.1 %)		2 (4.8 %)	3 (8.8 %)		3 (8.1 %)	4 (20 %)	
	Normal	19 (6 %)	11 (6.5 %)		58 (15.4 %)	28 (20.1 %)		42	34		37	20	

Results are presented as number (%) or median (range). *P*-values are for Kruskal-Wallis test or Fisher’s exact test; *statistically significant. Samples with missing data are not shown. *Death* breast cancer-specific death, *Size* tumor size, *Node* lymph node status, *Grade* tumor grade, *ER* estrogen receptor expression status defined by gene expression data, *Her2* human epidermal growth factor receptor 2 status defined by SNP6 copy number data, *TP53 TP53* mutation status, *WT* wild type, *IA* immune abundance, *Pam50* intrinsic subtypes, Pos positive, Neg negative, *LumA* luminal A, *Lum B* luminal B

### Immune-cancer cell colocalization measured by the Morisita-Horn ecological index is associated with good prognosis in unselected breast cancer

**Fig. 2:**
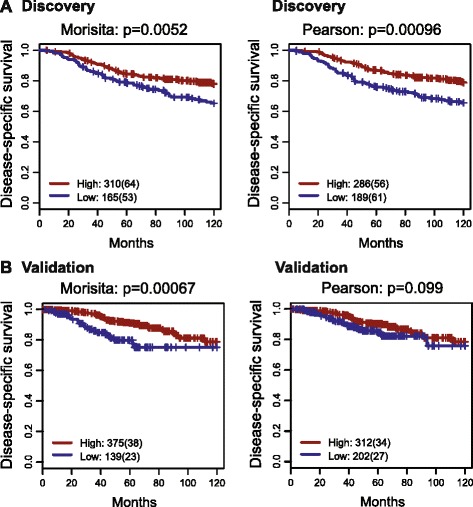
Association between immune-cancer cell colocalization and disease-specific survival in breast cancer. Kaplan-Meier curves illustrate disease-specific survival probabilities of patient groups in two subsets stratified by the Morisita index and Pearson correlation in the discovery (**a**) and validation (**b**) cohorts. The thresholds for dichotomizing two indices were optimized in the discovery cohort and then used without modification in the validation cohort (0.6940734 for the Morisita index and 0.4884236 for Pearson correlation). Numbers in the legend show the number of patients in each group and numbers in brackets show the number of disease-specific deaths

We then examined these measures and their association with 10-year breast cancer disease-specific survival. The optimal cutoff for dichotomizing each measure was selected using the discovery cohort (n = 475) and then tested in the independent validation cohort (n = 514, “Methods”). For both measures, a higher score was associated with significantly better disease-specific survival in the discovery cohort (Morisita-Horn index *p* = 0.0052, HR = 0.6, 95 % CI = 0.42 − 0.86; Pearson correlation *p* = 0.00096, HR = 0.55, 95 % CI = 0.38 − 0.79; Fig. 2a), suggesting that a high degree of immune-cancer colocalization is associated with good prognosis. While this association for the Morisita-Horn index was confirmed in the validation cohort (*p* = 0.00067, HR = 0.41, 95 % CI = 0.25 − 0.7), Pearson correlation was not statistically significant in the validation cohort (*p* = 0.099; Fig. 2b). We further investigated different spatial parameters used for the calculation of the two measures, including a Voronoi vs square tilling/tessellation and different polygon sizes (Additional file 1, Additional file 2: Figure S2-S5). We found that the combination of the Morisita-Horn index with square tessellation is robust to polygon size and the most prognostic, although Voronoi tessellation yielded scores with a higher correlation with visual scoring of colocalization (Pearson correlation *p* = 5.6 × 10^−5^), indicating that the visual estimation of colocalization by the pathological criteria used in this study integrates an adjustment for cell density somewhat similar to the Voronoi tessellation. In summary, our data suggest that colocalization of cancer cells and immune cells, as indicated by high scores of the Morisita-Horn index (henceforth referred to as the Morisita index), predicts favorable prognosis in breast cancer.

### Immune-cancer cell colocalization is associated with a good prognosis in Her2+ and luminal A tumors

**Fig. 3:**
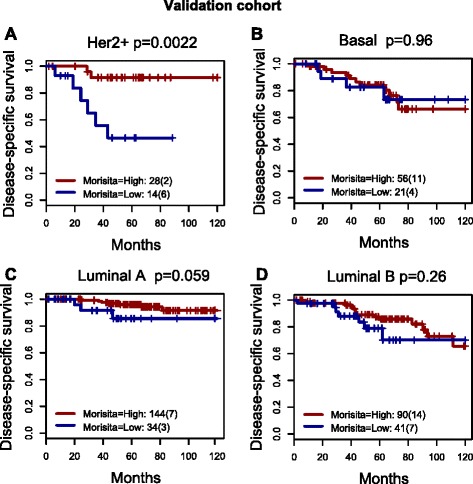
The association between immune-cancer cell colocalization and breast cancer prognosis is highly subtype-specific. Kaplan-Meier curves for the validation cohort alone are shown for human epidermal growth factor receptor 2-positive (*Her2*+) (**a**), basal (**b**), luminal A (**c**), and luminal B (**d**) Pam50 subtypes. The thresholds for dichotomizing two indices were optimized in the discovery cohort of the subtype and then used for both the discovery and validation cohorts (0.639985 for luminal A, 0.7439517 for luminal B, 0.699701 for basal and 0.7106531 for Her2)

We investigated the association between colocalization with the intrinsic molecular subtypes defined by the PAM50 gene set. Using an optimal threshold derived from the same subtype in the discovery cohort (Additional file 2: Figure S6), we found a significant association with favorable prognosis in the Her2 subtype for both cohorts (discovery *p* = 0.00017, HR = 0.29, 95 % CI = 0.15 − 0.57; validation *p* = 0.0022, HR = 0.12, 95 % CI = 0.02 − 0.61; Table 2; Fig. 3) and a significant association with favorable prognosis in the discovery and a borderline effect in the validation cohort for the luminal A subtype (discovery *p* = 0.0014, HR = 0.13, 95 % CI: 0.04–0.45; Validation *p* = 0.059, HR = 0.29, CI: 0.07–1.14; Table 2). In basal or luminal B subtypes, there was no association with prognosis in the discovery or validation cohort (Additional file 2: Table S2).

**Table 2:** Prognostic value of immune-cancer cell colocalization measures using univariate (white background) and multivariate (gray background) Cox regression in the discovery and validation cohorts for all breast cancers and Her2+ cancer defined by PAM50 and by SNP6

		Hazard ratio (95 % CI)	*P* value	Conc	Hazard ratio (95 % CI)	*P* value	Conc
All breast cancers		Discovery cohort (475 samples)			Validation cohort (514 samples)		
	Morisita	0.6 (0.42–0.86)	**0.0052**	0.56	0.41 (0.25–0.7)	**0.00067**	0.75
	Lymphnode	2.36 (1.62–3.46)	9.4×10^−6^	0.607	3.55 (1.92–6.56)	5.4×10^−5^	0.73
	Size	2.26 (1.63–3.13)	8.9×10^−7^	0.612	2.42 (1.57–3.73)	6.5×10^−5^	0.74
	Grade	2.18 (1.59–3.01)	1.8×10^−6^	0.627	2.76 (1.66–4.59)	9.6×10^−5^	0.619
	Morisita	0.59 (0.41–0.85)	**0.005**	0.699	0.4 (0.24–0.69)	**0.00085**	0.9
	Lymphnode	1.78 (1.19–2.65)	0.0046		2.2 (1.16–4.17)	0.015	
	Size	1.89 (1.34–2.68)	0.00033		1.88 (1.17–3.03)	0.0096	
	Grade	1.82 (1.31–2.53)	0.00035		2.58 (1.53–4.34)	0.00038	
Luminal A		Discovery cohort (150 samples)			Validation cohort (178 samples)		
	Morisita	0.13 (0.04–0.45)	**0.00014**	0.715	0.29 (0.07–1.14)	0.059	0.618
	Lymphnode	1.43 (0.46–4.42)	0.54	0.552	1.93 (0.5–7.47)	0.33	0.576
	Size	4.38 (1.46–13.14)	0.0067	0.692	2.13 (0.69–6.62)	0.19	0.58
	Grade	1.84 (0.83–4.1)	0.13	0.619	2.47 (0.9–6.79)	0.071	0.62
	Morisita	0.15 (0.04–0.53)	**0.003**	0.839	0.33 (0.08–1.31)	0.11	0.733
	Lymphnode	1.01 (0.3–3.41)	0.98		1.18 (0.29–4.8)	0.81	
	Size	4.17 (0.99–17.56)	0.052		2.09 (0.54–8.14)	0.29	
	Grade	1.44 (0.61–3.42)	0.41		2.47 (0.86–7.13)	0.095	
*Her2* by PAM50		Discovery cohort (65 samples)			Validation cohort (42 samples)		
	Morisita	0.29 (0.15–0.57)	**0.00017**	0.65	0.12 (0.02–0.61)	**0.0022**	0.808
	Lymphnode	3.59 (1.55–8.3)	0.0015	0.64	NA	0.0098	0.666
	Size	1.74 (0.97–3.12)	0.064	0.577	4.83 (1.48–15.78)	0.0051	0.689
	Grade	1.68 (0.78–3.62)	0.18	0.574	0.29 (0.07–1.23)	0.075	0.529
	Morisita	0.38 (0.17–0.84)	**0.016**	0.734	0.1 (0.01–0.66)	**0.017**	0.896
	Lymphnode	2.2 (0.87–5.57)	0.095		NA	1	
	Size	1.47 (0.83–2.61)	0.18		0.78 (0.16–3.83)	0.76	
	Grade	1.8 (0.81–4.02)	0.15		0.37 (0.08–1.74)	0.21	
*Her2*+ by SNP6		Discovery cohort (72 samples)			Validation cohort (56 samples)		
	Morisita	0.49 (0.25–0.98)	**0.039**	0.597	0.05 (0.01–0.4)	**9.3×10** ^**−5**^	0.808
	Lymphnode	3.31 (1.5–7.31)	0.0017	0.637	7.31 (0.89–59.98)	0.031	0.666
	Size	1.49 (0.82–2.73)	0.19	0.557	3.52 (1–12.36)	0.05	0.689
	Grade	1.56 (0.68–3.59)	0.29	0.551	1.41 (0.36–5.58)	0.62	0.529
	Morisita	0.44 (0.21–0.91)	**0.027**	0.722	0.03 (0–0.3)	**0.0031**	0.896
	Lymphnode	3.68 (1.49–9.09)	0.0048		2.65 (0.28–24.99)	0.39	
	Size	2.01 (1–4.04)	0.049		3.42 (1.1–10.64)	0.034	
	Grade	1.46 (0.57–3.75)	0.43		1.86 (0.33–10.42)	0.48	

Multivariate Cox regression includes lymph node status, tumor size, tumor grade, and the colocalization measure; age as a continuous or dichotomized variable using the optimal cutoff search was not associated with survival in all cancers or any subtype and hence was not considered. Bold text indicates significant *p* values for the newly proposed measures. *Conc* concordance, *Her2*+ human epidermal growth factor receptor 2-positive

### Immune-cancer cell colocalization outperforms other scores of immune infiltrate in Her2+ cancer

**Fig. 4:**
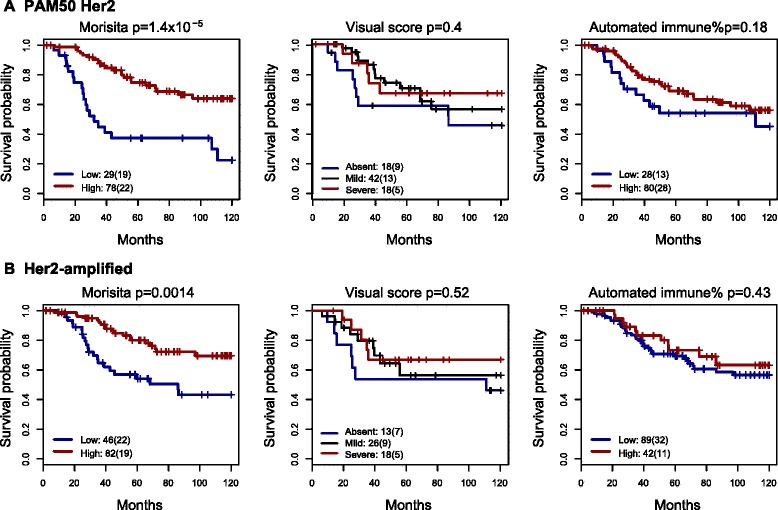
Immune-cancer cell colocalization is a strong prognostic factor in the Her2+ subtype, while immune cell abundance is not. Kaplan-Meier curves to show differences in disease-specific survival stratified by the Morisita index, visual and automated scores of immune abundance in human epidermal growth factor receptor 2 (*Her2*) subtype defined by PAM50 (**a**) and Her2-amplified (**b**) samples. Automated immune abundance was estimated as the percentage of cells that are lymphocytes in H&E images using a cutoff of 8 % (“Methods”)

To further examine the prognostic value of immune-cancer colocalization for Her2+ tumors, we compared the Morisita index with visual and automated scores of immune abundance in the Her2+ subtype defined using PAM50 gene expression-based subtyping or using Her2 amplification status from SNP6 copy number data (both cohorts combined, Fig. 4a-b). The Morisita index appeared to be superior to visual and automated scoring of immune abundance for patient stratification, highlighting the importance of investigating spatial patterns beyond cell abundance. In addition, two automated scores of immune response, namely intra-tumor lymphocyte ratio [29] and hotspots [16] were not found to be prognostic in these Her2+ subtypes (*p* >0.05 in the validation cohort). For both definitions of Her2+ cancer, the subtype-specific cutoffs for dichotomizing the Morisita index were the same (0.71). High Morisita index was again significantly correlated with good prognosis in the Her2-amplified samples (univariate analysis discovery: *p* = 0.039, HR = 0.49, 95 % CI = 0.25 − 0.98; validation: *p* = 9 × 10^−5^, HR = 0.05, 95 % CI = 0.01 − 0.4; Table 2).

### Immune-cancer colocalization is an independent prognostic factor in Her2+ cancer

Next, we examined the Morisita index with multivariate analyses for each cohort separately in Her2+ tumors. For both definitions of the Her2+ subtype, multivariate analysis showed prognostic value of the Morisita index independent of and stronger than those of node, size and grade in both cohorts (Her2-amplified discovery: *p* = 0.027, HR = 0.44, 95 % CI: 0.21–0.91; validation: *p* = 0.0031, HR = 0.03, 95 % CI = 0–0.3; Table 2). Multivariate analysis revealed additional prognostic value of the Morisita index given any of the clinicopathologic variables tested including grade, node size, *ER* status, immune abundance and *TP53* mutation (Additional file 2: Figure S7). Moreover, this association was consistently observed when using bootstrap analysis to test robustness due to small sample sizes in some of the comparisons (Additional file 1, Additional file 2: Figure S8). In another test using decreasing amounts of tissue, the Morisita index remained prognostic in Her2+ cancer with as little as 50 % of tissue (Additional file 1, Additional file 2: Figure S9-10).

### Immune-cancer cell colocalization predicts long-term outcome after chemotherapy and radiotherapy in Her2+ cancer

**Fig. 5:**
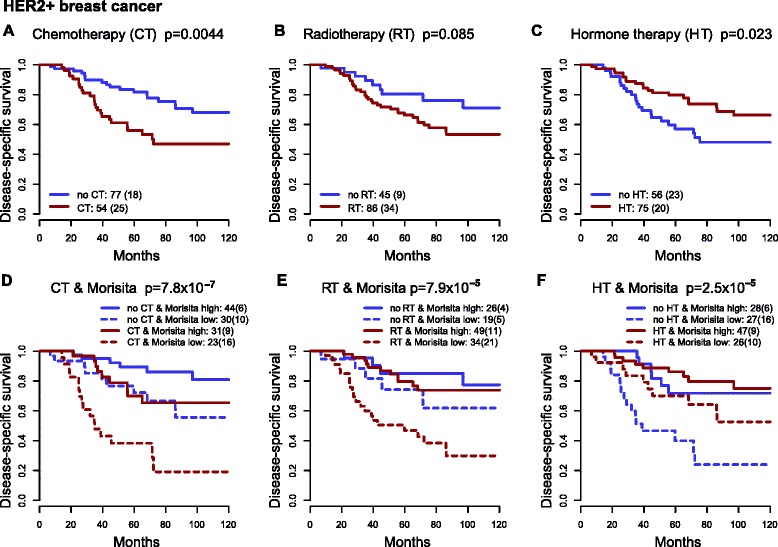
Immune-cancer cell colocalization predicts long-term outcome following chemotherapy (*CT*), radiotherapy (*RT*) and hormone therapy (*HT*) in human epidermal growth factor receptor 2-positive (*Her2+*) breast cancer. Kaplan-Meier curves show differences in disease-specific survival between Her2+ patients with or without CT (**a**), RT (**b**) and HT (**c**). The Morisita index significantly stratifies disease-specific survival for both treated and untreated Her2+ patients, shown by CT treatment and Morisita index (**d**), RT treatment and Morisita index (**e**) and HT treatment and Morisita index (**f**)

We investigated the influence of treatment options including CT, RT and HT on the Her2-amplified subtype and whether outcomes from these treatments were different according to the Morisita index. Patients did not receive anti-Her2 therapies. We found that effect of patient stratification by Morisita was independent of treatments given to these patients (multivariate analysis with Morisita, CT, RT and HT: Morisita *p* = 1.3 × 10^−4^, HR = 0.28, 95 % CI = 0.15 − 0.54; Additional file 2: Table S3). Subsequently, we examined outcome differences separately for each type of treatment given the Morisita index. Patients with late-stage disease were more likely to be given aggressive treatments such as CT and RT, and this was shown in their long-term prognosis (Fig. 5a-c). Nevertheless, CT-treated patients with a high Morisita index had a 65 % survival probability 10 years after diagnosis, significantly better than the 19 % survival probability for patients with a low Morisita index (Fig. 5d, solid red versus dashed red curve, *p* = 0.00065, HR = 0.26, 95 % CI = 0.11 − 0.59). Furthermore, the Morisita index further stratified patients who had not received CT (chemo-naïve) (solid blue versus dashed blue curve; *p* = 0.033, HR = 0.34, 95 % CI = 0.12 − 0.96) and had a 10-year survival probability of 80 % (solid black curve, Fig. 5d), an outlook rare for this subtype before the availability of Her2-inhibitors. Similarly, for other treatments, a low Morisita index also further pinpointed aggressive cancers in the RT-treated (90 patients) and HT-naïve groups (60 patients), after controlling for all these clinicopathologic variables (Fig. 5e and f, Additional file 2: Table S3).

### Immune-cancer cell colocalization is clinical relevant for long-term treatment outcome in the luminal A subtype

**Fig. 6:**
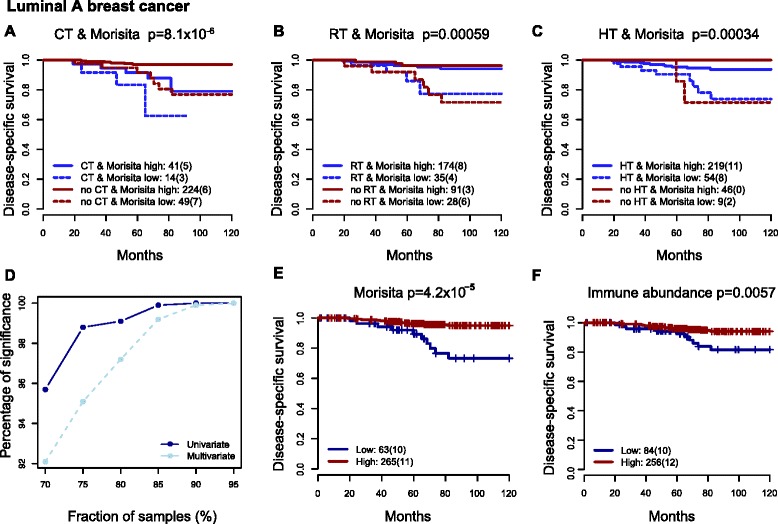
Immune-cancer cell colocalization predicts long-term outcome following radiotherapy (*RT*) and hormone therapy (*HT*) in luminal A breast cancer. Kaplan-Meier curves show differences in disease-specific survival stratified by CT treatment and Morisita index (**a**), RT treatment and Morisita index (**b**) and HT treatment and Morisita index (**c**). (**d**) Percentage of times where Morisita was statistically significant in univariate and multivariate analysis with different amounts of patient samples that were selected randomly 1000 times. Comparison of Morisita (**e**) and automated score of immune abundance (**f**) based on association with disease-specific survival in luminal A cancer

In a similar vein, Morisita was predictive of outcome in patients with luminal A breast cancer who were chemo-naïve (273 patients, *p* = 0.0006, HR = 0.14, 95 % CI = 0.05 − 0.44), RT-treated (209 patients, *p* = 0.03, HR = 0.26, 95 % CI = 0.08-0.89), RT- naïve (119 patients, *p* = 0.005, HR = 0.13, 95 % CI = 0.03 − 0.54), and HT-treated (273 patients, p = 0.0035, HR = 0.25, 95 % CI = 0.1 − 0.64; Fig. 6a-c). In addition, when all treatments and clinical parameters including grade, node, size were considered together, Morisita was the only variable that was significant in a multivariate model (*p* = 0.0008, HR = 0.22, 95 % CI = 0.19 − 0.53) and was further validated with bootstrap analysis (>95 % of the times Morisita was significant with more than 75 % (n=225) of luminal A patients being randomly taken), (Fig. 6d). We found a clear advantage of the Morisita index compared with image analysis-based (Morisita: p=4.2x10^-5^, HR=0.19, 95% CI=0.08-0.47; IA: p=0.0057, HR=0.32, 95% CI=0.14-0.75; Fig. 6e and f) or visual scoring for immune abundance (*p* >0.1).

## Discussion

There is a wide diversity of outcomes for breast cancer, and differential responses to treatments. Reproducible biomarkers that can identify which patients are likely to follow a benign course and which would benefit from chemotherapy or radiotherapy would dramatically improve clinical care. We combined digital pathology with an ecological measure, the Morisita index, to study the spatial colocalization of cancer cells and immune cells. This is analogous to analyzing predator–prey interactions in an ecosystem. Our study, in 1002 breast cancer patients using routine histology sections stained with H&E, reveals that colocalization of cancer cells and immune cells is a significant indicator of favorable survival, independent of and stronger than standard clinical variables, particularly in Her2+ breast cancer. It is a fully reproducible and highly prognostic measure of immune response. This biomarker could be developed into computer-assisted prediction tools for routine pathology and clinical use.

The association between immune-cancer cell colocalization and favorable prognosis is indicative of continued effectiveness of the immune system to identify and target cancer cells to limit their survival, proliferation or invasion. Just as prey can evolve complex predator avoidance adaptations, cancer cells can likewise evolve complex adaptations to evade immune predation [30]. The Morisita index, which has been used to study predator–prey relationships in ecology, thus provides a quantitative measure of immune-cancer colocalization and potential immune predation in specific breast cancers.

Our ecological measure further revealed clinically relevant information about long-term benefit of specific treatments in Her2+ breast cancer. Patients with late-stage disease are more likely to be treated with chemotherapy, yet these patients with a high Morisita index had significantly better outcomes than patients with a low Morisita index, revealing a strong link between immune predation and long-term benefit from chemotherapy even in aggressive Her2+ cancer. This is consistent with evidence that immune-infiltrated Her2+ cancer is highly sensitive to immune-mediated cytotoxic treatments such as chemotherapy [31, 32]. Although our automated scoring of immune-cancer colocalization is conceptually different from the stromal lymphocytic infiltration reported to correlate with benefit from chemotherapy in the neoadjuvant setting [14], our findings support an important role for immune cells in the success of therapy and provide a tool for identifying high-risk patients following chemotherapy for continuous monitoring, and the two types of scores remain to be compared.

Since these data were collected, the standard of care for Her2+ patients has changed with the introduction of Her2-targeted therapies. However, it has been argued that an active immune response is also critical for Her2-inibition treatment [33, 34]. Certain Her2 inhibitors such as trastuzumab are known to mediate their tumor killing effects through the immune system [35, 36]. Furthermore, promising new treatment paradigms for these cancers may involve the addition of anti-PD1 or anti-CD137 immunotherapies to stimulate IFNγ-producing CD8+ T cells and maximize benefits from Her2 inhibitors such as trastuzumab [34] or other therapies [37, 38]. Therefore, our proposed ecological measure of immune predation may be also effective for identifying patients who could benefit from such therapies to reactivate immune programs and for monitoring the success of those therapies in stimulating immune predation of the cancer cells.

Our data show a significant difference in prognosis or long-term benefit of therapies according to immune-cancer colocalization in Her2+ and luminal A tumors, but not triple-negative or basal tumors. However, abundance of lymphocytes has been found to be a prognostic biomarker in triple-negative breast cancer in multiple studies [39, 40], including our previous report on the triple-negative subtype in our cohort [29]. These suggest that there are different ecological dynamics with immune predation in basal versus Her2+ subtypes, and that our ecological measure reveals a distinction in a specific pattern of immune response that was not previously recognized. Our results also highlight the subtype specificity of immune infiltrate patterns and warrant a comprehensive future study on this, including the spatial clustering pattern we recently reported [16], across all breast cancer subtypes.

A limitation in our study is the lack of immunohistochemistry experiments to identify the types of immune cells that are infiltrating (or being excluded from) the tumor. Studying spatial colocalization in the context of immune subsets including T-regulatory and T-effector cells aided by multi-color staining will be a powerful approach for defining the spatial and molecular heterogeneity of the tumor microenvironment, and we are currently pursuing this. In addition, further validation is needed for stratification based on treatment options. We observed increasing variability in the Morisita scores with decreasing amounts of tissue in our test of intra-slide variability with decreasing amounts of tissue (Additional file 1, Additional file 2: Figure S9-10). This is not unexpected, because the Morisita index is a global statistic of colocalization pattern and so loses statistical power as the amount of tumor material decreases. We recommend using tumor sections and not needle biopsy or tissue microarrays for these measures. We found that Morisita remains prognostic with 50 % of tissue (mean 33,826 cells), suggesting that the Morisita index can be applied as a prognostic marker when tissue area is sufficiently large (>46,230 cells, third quantile of cell numbers in 50 % tissue).

## Conclusions

Taken together, we postulate that immune predation exhibits different spatial patterns under specific contexts, which should be accounted for during biomarker development for a specific cancer subtype. The addition of a spatial analysis to the evaluation of the types of immune cells in the microenvironment with immunohistochemistry experiments can reveal further details in the ecological interactions between the neoplastic cells and the immune system. In summary, our study demonstrates the power of applying quantitative image analysis, spatial statistics and ecological theory to the study of tumor microenvironments and the routine pathological assessment of breast cancer. These approaches can be used both for clinical benefit and for revealing the ecological dynamics that are driving any cancer.

### Data and software availability

All data have been deposited at the European Genome-Phenome Archive hosted by the European Bioinformatics Institute [EGAS00000000083]. R code and the dataset for performing the proposed methodology and reproducing reported results are provided as Additional file 3: Sweave file for full reproducibility.

## Additional files

Additional file 1:
**Supplementary methods.** Supplementary information on sample materials and methods. (DOCX 116 kb) Additional file 2: Figure S1. Supplementary Figures and Tables. This file contains the following figures and tables: Validation of image analysis with pathological scores. **Figure S2.** Comparison of Voronoi and Square tessellation. **Figure S3.** Comparing the Morisita-Horn index with Pearson correlation. **Figure S4.** Heatmap to show correlation among Pearson correlation and the Morisita-Horn index computed over different scales with square (S) or Voronoi (V) tessellation. **Figure S5.** Association of immune-cancer correlation with survival according to different spatial scales. **Figure S6.** Optimizing the Morisita index cutoff in the discovery cohort. **Figure S7.** Immune-cancer colocalization sub-stratifies clinical parameters and immune abundance, estrogen receptor (*ER*) status and *TP53* mutation status in human epidermal growth factor receptor 2 (HER2) + subtype. **Figure S8.** Robustness of prognostic value of Morisita index in HER2-amplified subtype. **Figure S9.** Intra-slide score variability. **Figure S10.** Kaplan-Meier curves of the Morisita index estimated using decreasing amount of tissue. **Table S1.** Prognostic value of immune-cancer colocalization measures with Voronoi tessellation using univariate and multivariate Cox regression results in two independent cohorts. **Table S2.** Univariate and multivariate Cox regression analysis result with the Morisita index, node, size and grade in four PAM50 subtypes and HER2-amplified subtype defined by SNP6 microarray with two cohorts. **Table S3.** Groups of multivariate Cox proportional hazard analysis of the Morisita index and treatment, known clinical or other variables in HER2-amplified, HER2-amplified and chemotherapy (CT)-treated, and HER2-amplified and radiotherapy (RT)-treated patients. (DOCX 6725 kb) Additional file 3:
**Sweave file.** R code used for reproducing all results presented in the paper. (PDF 600 kb)

## Abbreviations

CT: chemotherapy
ER: estrogen receptor
H&E: hematoxylin and eosin
Her2: human epidermal growth factor receptor 2
HR: hazard ratio
HT: hormone therapy
IFN: interferon
JT: Jonckheere-Terpstra
RT: radiotherapy
