# Supplementary material for: An ecological measure of immune-cancer colocalization as a prognostic factor for breast cancer
Source: Breast Cancer Res. 2015 Sep 22;17(1):131. doi: 10.1186/s13058-015-0638-4 (PMC4579663; doi:10.1186/s13058-015-0638-4)
Supplement: Additional file 1: — Supplementary methods. Supplementary information on sample materials and methods. (DOCX 116 kb) [file 13058_2015_638_MOESM1_ESM.docx]

**Supplementary Methods**

**Clinical samples**

The complete set of METABRIC (1) samples contains 1,980 primary frozen breast tumors from five contributing hospitals. Among these, 1,026 tumors from three hospitals have good quality H&E sections, whist the H&E samples from the other two hospitals are highly fragmented due to long-term frozen storage. Of these, 24 samples did not contain sufficient tissue for analysis of colocalization. Therefore, we only considered the remaining 1,002 tumors for this study, 989 of which complete follow-up for disease-specific survival was available (median 68.3 months). On average three tumor sections were obtained for each primary tumor and placed onto the same slide (2). Estrogen Receptor (ER*)* status was determined based on the bimodal distribution of *ESR1* expression microarray data since ER status by immunohistochemistry was not available for all samples. The Epidermal growth factor receptor 2 (HER2*)* status was determined by SNP6 amplification. Our image analysis tool was used to classify all whole-tumor sections, resulting in an average of 63,230 cancer cells, 10,670 immune cells and 12,940 stromal cells for each image.

**Spatial configuration with tessellations**

For square tessellation, a fixed square size *h*-by-*h* was used for all tumors. First, *h*=50 was used as the fixed square length and tumor sections were divided into squares of size 50x50, where each unit is 5um. For analysis of different scales, a range of squares of width *h*=40, 50, 60, 70, 80, 100, 150, 250 was used. For Voronoi tessellation, a tessellation is a division of topolographical space initiated by a set of *S* spatial points or seeds*.* Voronoi diagram, as one of the simplest tessellation methods, assigns the smallest convex polygon to a seed *P_i_* whose sides are the perpendicular bisectors of the lines joining *P_i_* to the other points. It is an exhaustive and exclusive model of the topology because a point is assigned to and only to a polygon. To generate Voronoi polygons for a tumor section, cancer cells were randomly sampled and used as ‘seeds’ for tessellation. The number of seeds *S* was chosen so that a similar number of polygons with the square tessellations was obtained. Let $S=\frac{1}{b}\sqrt{D}$, so that *S* scales non-linearly to *D* where the number of tissue pixels is denoted *D*. *b* was estimated to be 3 in 30 randomly sampled tumors to obtain approximately same number of polygons as in square lattices of 50x50. *S* cancer cells were then randomly sampled to produce Voronoi polygons using the R package sp (39). For analysis of different spatial scales, a range of *b*=2,3,4,5,8,10,50,100 was used.

**Measuring spatial colocalization of cancer cells and immune cells**

The number of immune cells and cancer cells for each polygon *i* are obtained and denoted as $n_{i}^{l}$and $n_{i}^{c}$, based on either Voronoi or square tessellation (Supplementary Methods). Polygons with low cell density were removed from subsequent analysis. This was determined by $\frac{n_{i}}{d_{i}}\leq0.02$ for a polygon *i*, $n_{i}$ being the total number of cells and $d_{i}$ being size in pixel of the polygon *i*. Spatial correlation was computed using Pearson correlation and Morisita-Horn’s similarity index with the number of cancer and immune cells in each polygon *i*, $n_{i}^{l}$and $n_{i}^{c}$as input. Let $p_{i}^{l}$ and $p_{i}^{c}$ denote the fraction of immune cells and cancer cells in polygon *i*, i.e. $p_{i}^{l}=\frac{n_{i}^{l}}{\sum_{i} n_{i}^{l}}$*,* $p_{i}^{c}=\frac{n_{i}^{c}}{\sum_{i} n_{i}^{c}}$*,* Morisita-Horn’s similarity index between the cancer and immune community structure in a tumor is:

$$M=\frac{2\sum_{i} p_{i}^{l}p_{i}^{c}}{\sum_{i} {(p_{i}^{l})}^{2}+\sum_{i} {(p_{i}^{c})}^{2}}.$$

**The Morisita index is a robust measure for studying the tumor microenvironment**

Because cell densities vary across a tissue section, we tested a Voronoi tessellation of the images, so as to produce a more Normal distribution of cell numbers across the regions being measured (Methods, Fig. S2a for an example). Subsequently, the number of cancer cells and the number of lymphocytes within each of the polygons was obtained (Fig. S2b). Anderson-Darling normality test was used for assessing the distribution of cell numbers in polygons for each tumor. For 50 randomly selected tumors, the numbers of cells per square and per tessellation polygon were computed and Anderson-Darling test was employed to compute the A statistics, for which a high score indicates that the distribution is less likely to be normal-like. As we hypothesized, the Voronoi tessellation indeed generated a more normal-like distribution of cell numbers in the polygons than the Square tessellation as determined by the Anderson-Darling normality test (5) (mean A=7.7 for Voronoi and 14.9 for square, averaged over 50 random samples; Fig. S2c-d). Scores from the Morisita index calculated using both lattice tessellation methods were highly similar (cor=0.96, Fig. S3a), supporting its robustness to data distribution. In contrast, scores from Pearson correlation based on the two tessellation methods were less similar (cor=0.66, Fig. S3b). Except for Pearson correlation with square lattice, both measures based on the two tessellations were significantly associated with breast cancer prognosis. The Voronoi tessellation improved the predictive power of the Pearson correlation for disease-specific survival (p=0.0029 in the Voronoi tessellation and p=0.099 on the square tessellation, Validation cohort; Fig. S3c). However, the Morisita index was highly statistically significant (p<0.001) for both cohorts (Fig. S3d, Table S1).

Since spatial scales have been shown to be important parameters of ecological studies (6), we investigated the influence on our analysis from the use of different spatial scales for both square and Voronoi tessellations. Morisita index and Pearson correlation were computed based on square and Voronoi tessellation of eight different scales, where larger scale indicates larger regions and thus a smaller number of data points per tumor (Methods). Correlation heatmaps of these results show highly correlated Morisita index regardless of the type or scale of tessellation (Fig. S4). Pearson correlation computed with Voronoi polygons show highly variable results, and correlations computed with squares were correlated among themselves but not with Morisita scores (Fig. S4). Analysis of their association with survival also shows that Pearson correlation and the Morisita index computed with Voronoi tessellation yielded more variable result over different scales (Fig. S5a). In contrast, with square tessellation the Morisita index is consistently associated with survival over all scales (Fig. S5). These results demonstrate the robustness of the Morisita index for studying the tumor microenvironment over different spatial configurations, and we henceforth focused on the Morisita index based on the simple 250μm-by-250μm square tessellation for our subsequent analyses.

**The prognostic value of immune-cancer colocalization is independent of clinicopathologic variables in unselected breast cancers**

To investigate whether colocalization measures yield additional value to standard breast cancer clinical parameters (Table 1), we performed multivariate analysis. For unselected breast cancers, Morisita provides independent value in predicting improved disease-specific survival (Table 2; Morisita cut-off 0.69; Discovery p=0.005, HR=0.59, CI=0.41-0.85; Validation: p=0.00085, HR=0.4, CI 0.24-0.69) in addition to lymphnode metastasis, tumor size and tumor grade in both breast cancer cohorts. In particular, Morisita further stratified node positive (517 patients, p=0.0001, HR=0.5, CI=0.35-0.71), large tumor size (66 size 3 patients, p=0.002, HR=0.3, CI=0.13-0.68), and high-grade tumors (491 grade 3 patients, p=1.1x10^-5^, HR=0.46, CI=0.32-0.65). Age as a continuous or dichotomized variable using the optimal cut-off search was not associated with survival in all cancers or any subtype and therefore was not considered. Nevertheless, multivariate analysis with Morisita and its associated variables showed independent value of Moristia (Discovery p=0.026, HR=0.59, CI=0.37-0.94; Validation p=0.003, HR=0.36, CI=0.18-0.7).

**Bootstrap analysis**

As some of the cohorts we compared were relatively small such as HER+, we performed bootstrap analysis to test the robustness of Morisita by randomly sampling progressively lower amount of patients for univariate and multivariate survival analysis. Notably, in HER2-amplified cancers (as this includes more samples), Morisita was found to be significant for both univariate and multivariate analysis in >99% of the 1,000 random sampling procedures when more than 70% (96) HER2-amplified patients were sampled (Fig. S8a). In addition, even with different spatial scales, the Morista index was consistently prognostic in multivariate analysis controlled for grade, node and size (Fig. S8b). Taken together, these data consistently support the clinical relevance of cancer-immune colocalization measured by the Morisita index for patients with HER2 aberrations.

**Test of intra-slide variability**

**W**e split a slide into two parts by finding a line perpendicular to the longer axis of the slide that divide the slide into two parts containing roughly equal-size number of square polygons based on which the Morisita was computed. We chose this approach because in the METABRIC dataset tumor sections vary in size and the boundary of different sections are often difficult to define. Then we computed the Morisita index for the left and right parts independently and evaluated the variability. We repeated the same analysis but divided the slides into two parts containing 25% and 75% of the polygons. This is to evaluate the performance and variability of the Morisita index with decreasing amount of tissue.

We found that the differences in the Morisita index between whole-slide scores (W) and part-slide scores increase with decreasing amount of tissue area used for estimating part-slide scores (Fig. S9). Percentages of consistent classifications (Morisita-high and low) between whole-slide scores and estimates using 75% slide, 50% left, 50% right, 25% slide are 94.2%, 89.1%, 89.8%, 84.3%. In addition, we ran the same survival analysis of the Morisita scores estimated with decreasing amount of tissue in Her2+ subtype. We used the same method to find optimal cutoff in the discovery cohort and used the same cutoff in the validation. We found that estimates using 75% and 50% but not 25% of tissues remain prognostic in both discovery and validation cohort (Fig. S10).

**References**

1. Curtis C, Shah SP, Chin S-F, Turashvili G, Rueda OM, Dunning MJ, et al. The genomic and transcriptomic architecture of 2,000 breast tumours reveals novel subgroups. Nature. 2012 Jun 21;486(7403):346–52.

2. Yuan Y, Failmezger H, Rueda OM, Ali HR, Graf S, Chin S-FF, et al. Quantitative image analysis of cellular heterogeneity in breast tumors complements genomic profiling. Sci Transl Med. 2012/11/24 ed. 2012;4(157):157ra143.

3. Janowczyk A, S. C, Madabhushi A. Quantifying local heterogeneity via morphologic scale: Distinguishing tumoral from stromal regions. J Pathol Inf. 2013;4(Suppl).

4. Basavanhally AN, Ganesan S, Agner S, Monaco JP, Feldman MD, Tomaszewski JE, et al. Computerized image-based detection and grading of lymphocytic infiltration in HER2+ breast cancer histopathology. IEEE Trans Biomed Eng. 2009/11/04 ed. 2010;57(3):642–53.

5. Anderson TW, Darling DA. Asymptotic Theory of Certain “Goodness of Fit” Criteria Based on Stochastic Processes. The Annals of Mathematical Statistics. 1952. p. 193–212.

6. Rose GA, Leggett WC. The importance of scale to predator-prey spatial correlations: an example of Atlantic fishes. Ecology. 1990. p. 33–43.
