# Supplementary material for: An ecological measure of immune-cancer colocalization as a prognostic factor for breast cancer
Source: Breast Cancer Res. 2015 Sep 22;17(1):131. doi: 10.1186/s13058-015-0638-4 (PMC4579663; doi:10.1186/s13058-015-0638-4)
Supplement: Additional file 2: Figure S1. — Supplementary Figures and Tables. This file contains the following figures and tables: Validation of image analysis with pathological scores. Figure S2. Comparison of Voronoi and Square tessellation. Figure S3. Comparing the Morisita-Horn index with Pearson correlation. Figure S4. Heatmap to show correlation among Pearson correlation and the Morisita-Horn index computed over different scales with square (S) or Voronoi (V) tessellation. Figure S5. Association of immune-cancer correlation with survival according to different spatial scales. Figure S6. Optimizing the Morisita index cutoff in the discovery cohort. Figure S7. Immune-cancer colocalization sub-stratifies clinical parameters and immune abundance, estrogen receptor (ER) status and TP53 mutation status in human epidermal growth factor receptor 2 (HER2) + subtype. Figure S8. Robustness of prognostic value of Morisita index in HER2-amplified subtype. Figure S9. Intra-slide score variability. Figure S10. Kaplan-Meier curves of the Morisita index estimated using decreasing amount of tissue. Table S1. Prognostic value of immune-cancer colocalization measures with Voronoi tessellation using univariate and multivariate Cox regression results in two independent cohorts. Table S2. Univariate and multivariate Cox regression analysis result with the Morisita index, node, size and grade in four PAM50 subtypes and HER2-amplified subtype defined by SNP6 microarray with two cohorts. Table S3. Groups of multivariate Cox proportional hazard analysis of the Morisita index and treatment, known clinical or other variables in HER2-amplified, HER2-amplified and chemotherapy (CT)-treated, and HER2-amplified and radiotherapy (RT)-treated patients. (DOCX 6725 kb) [file 13058_2015_638_MOESM2_ESM.docx]

**Supplementary Figures and Tables**

**Figure S1. Validation of image analysis with pathological scores**. (left to right): Correlation between pathological scores of tumor cellularity and automated scores of cancer cell to tissue area ratio, cancer cells to all cells ratio; correlation between pathological scores of lymphocytic infiltration and automated scores of lymphocyte to all cells ratio. P-values are given by JT-trend test.

**
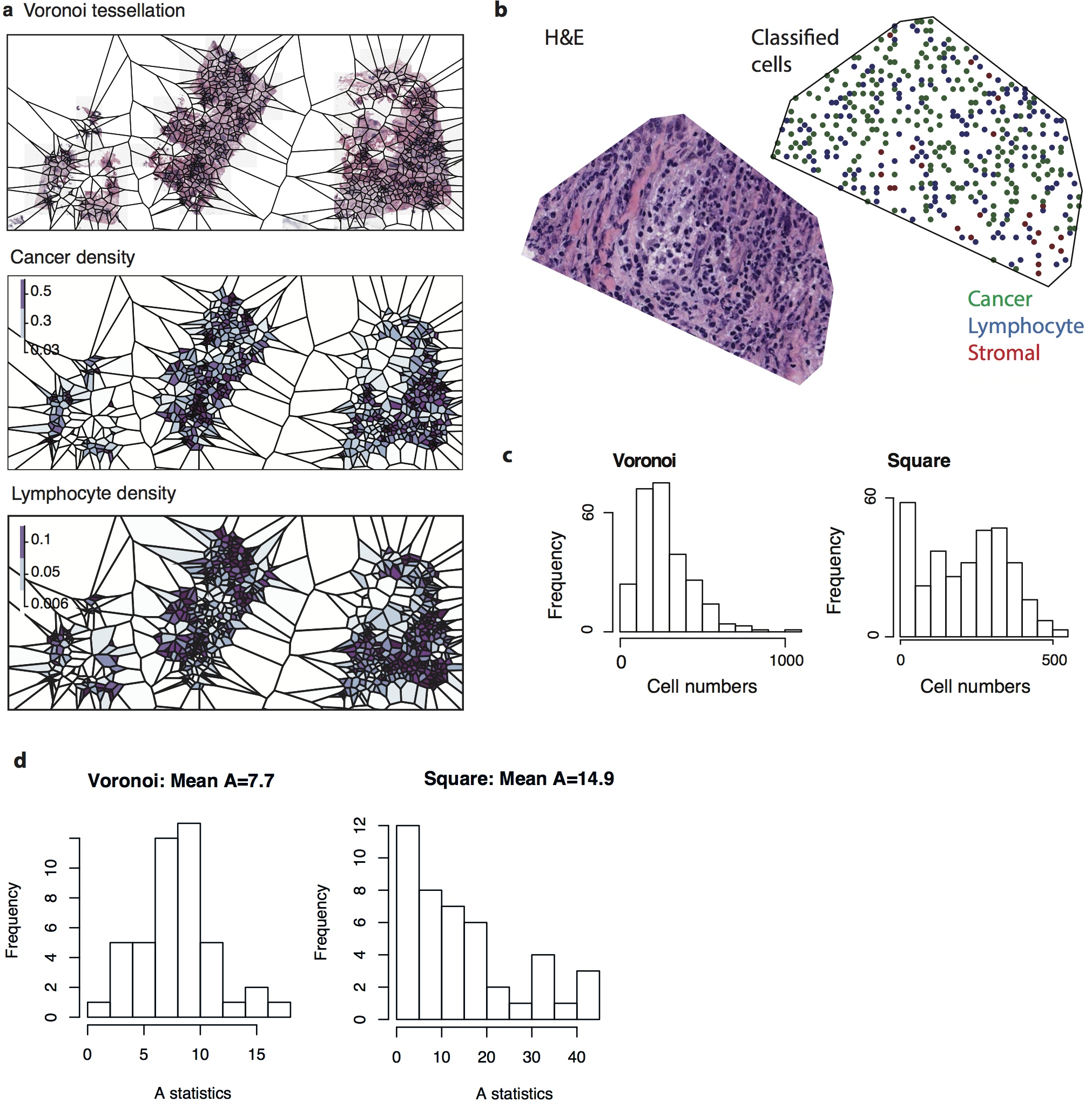
**

**Figure S2. Comparison of Voronoi and Square tessellation. (a)** An example of Voronoi polygons in the sample shown in Fig.1. **(b)** High resolution view of a Voronoi polygon; shown are the H&E and identified cells in the polygon. **(c)** Distribution of the number of cells in all polygons in this sample. (**d**) Distribution of the Anderson-Darling A statistics for cell numbers in Voronoi and square polygons of 50 randomly sampled tumors.

**Figure S3. Comparing the Morisita-Horn index with Pearson correlation. (a)** Scatter plot to show correlation between Pearson correlation calculated with Square and Voronoi tessellation. **(b)** Scatter plot to show correlation between the Moristia-Horn index calculated with Square and Voronoi tessellation. (**c**) Kaplan-Meier curves illustrate disease-specific survival probabilities of patient groups in two breast cancer subsets stratified by Pearson correlation or **(d)** the Morisita-Horn index, calculated using Voronoi tessellation.

**Figure S4. Heatmap to show correlation among Pearson correlation and the Morisita-Horn index computed over different scales with square (S) or Voronoi (V) tessellation.** Scale size for Voronoi tessellation ranges from 2-100, and for squares ranges from 40-250 per unit, where for both smaller numbers indicate smaller polygons.

**Figure S5. Association of immune-cancer correlation with survival according to different spatial scales.** –log_10_ p-values from **(a)** univariate Cox regression model and **(a)** multivariate Cox model with node, size and grade for Pearson correlation and the Morisita-Horn index computed over different scales with square (S) or Voronoi (V) tessellation in the validation cohort. (c) –log_10_ p-values from multivariate Cox model in HER2+ validation cohort.

**Figure S6. Optimizing the Morisita index cut-off in the Discovery cohort.** For each subtype, left panel: a cut-off was chosen from a range of 20%-80% and correlation with disease-specific survival computed. The dashline marks the significance level of *p*=0.05; right panel: Kaplan-Meier curves display the difference in survival according to this cutoff.

**Figure S7. Immune-cancer colocalization sub-stratifies clinical parameters and lymphocyte abundance, *ER* status and *TP53* mutation status in HER2+ subtype.** Kaplan-Meier curves to show differences in disease-specific survival between Her2+ patients stratified by (top, variable alone, bottom: variable plus Morisita index): (**a**) node metastasis status, (**b**) tumor size (3 or <3 because there is no difference in survival for size 1 and 2), (**c**) tumor grade (3 or <3, because there are only 3 Grade 1 patients), (**d**) lymphocyte abundance (lym), (**e**) *ER* status, and (**f**) *TP53* mutation status.

**Figure S8. Robustness of prognostic value of Morisita index in HER2-amplified subtype.** (**a**) Percentage of times where Morisita was found to be significant in uni- and multivariate analysis with different amount of patient samples that were selected randomly 1,000 times. (**b**) Log-rank test p-values of Moristia in multivariate survival analysis of the HER2-amplied samples, Validation cohort, over different spatial scales represented as the square widths used for tessellation.

**Figure S9. Intra-slide score variability**. Top left: Barplots showing number of samples with consistent/difference classifications between whole-slide scores and estimates using 50% left/right part of the slides (W-L/R cons./differ), as well as consistent/difference classifications between 50% left and right parts of the slide (L-R cons./differ). Top right: Barplots showing number of samples with consistent/difference classifications between whole-slide scores and estimates using 25%/75% slide (W-25%/75% cons./differ), as well as consistent/difference classifications between them. Bottom left: Scattered plots to show absolute differences between the whole-section scores (100%) and 75% slide scores compared with differences between 100% and 25% scores. Each point is a sample. Bottom right: Scattered plots to show absolute differences between the whole-section scores (100%) and 75% slide scores compared with differences between 100% and 50% scores.

**Figure S10. Kaplan-Meier curves of the Morisita index estimated using decreasing amount of tissue: A**. 75% tissue of the slide, **B**. 50% tissue on the right hand side, **C**. 50% tissue on the left hand side, **D**. 25% tissue.

**Table S1. Prognostic value of immune-cancer co-localization measures with Voronoi tessellation using univariate and multivariate Cox regression results in two independent cohorts**. Blue sections show results from multivariate regression. Uni-: Univariate Cox regression; Multi-: Multivariate Cox regression including node, size and grade as well as the co-localization measure; HR: Hazard Ratio; CI: lower and higher 95% Confidence Interval; Conc: Concordance. Bold text show p-values of the newly proposed measures.

|  | | **Cohort 1 (475 samples)** | | | **Cohort 2 (514 samples)** | | |
| --- | --- | --- | --- | --- | --- | --- | --- |
|  |  | HR(CI) | *p*-value | Conc | HR(CI) | *p*-value | Conc |
| Spatial  Cor  (Voronoi) | Uni- | 0.58 ( 0.37 - 0.91 ) | **0.016** | 0.549 | 0.38 ( 0.2 - 0.74 ) | **0.0029** | 0.597 |
|  | Multi-  Multi-node  Multi-size  Multi- grade | 0.52 ( 0.33 - 0.82 )  1.81 ( 1.21 - 2.71 )  1.93 ( 1.36 - 2.72 )  1.85 ( 1.33 - 2.57 ) | **0.0051**  0.0038  0.00021  0.00025 | 0.702 | 0.37 ( 0.18 - 0.73 )  2.25 ( 1.19 - 4.26 )  1.96 ( 1.21 - 3.17 )  2.44 ( 1.46 - 4.09 ) | **0.0042**  0.013  0.0064  0.00071 | 0.756 |
| Morisita  (Voronoi) | Uni- | 0.59 ( 0.4 - 0.86 ) | **0.0068** | 0.552 | 0.38 ( 0.22 - 0.66 ) | **0.00027** | 0.611 |
|  | Multi-  Multi-node  Multi-size  Multi- grade | 0.63 ( 0.43 - 0.92 )  1.77 ( 1.18 - 2.64 )  1.93 ( 1.36 - 2.74 )  1.82 ( 1.31 - 2.53 ) | **0.018**  0.0056  0.00023  4.0x10^-4^ | 0.699 | 0.39 ( 0.23 - 0.67 )  2.19 ( 1.16 - 4.13 )  1.82 ( 1.14 - 2.92 )  2.58 ( 1.53 - 4.35 ) | **0.00072**  0.016  0.012  0.00036 | 0.76 |

**Table S2. Univariate and multivariate Cox regression analysis result with the Morisita index, node, size and grade in the Basal and Luminal B PAM50 subtypes in two cohorts**. Uni-: Univariate Cox regression; Multi-: Multivariate Cox regression; HR: Hazard Ratio; CI: lower and higher 95% Confidence Interval; Conc: Concordance; Inf: Infinite number indicating the Cox model didn’t converge. Bold text show significant p-values (<0.05) of the Morisita index. Numbers under subtype names show the number of samples in each subtype, two cohorts combined.

|  | | **Cohort 1** | | | **Cohort 2** | | |
| --- | --- | --- | --- | --- | --- | --- | --- |
|  |  | HR(CI) | *p*-value | Conc | HR(CI) | *p*-value | Conc |
| Basal  (164) | Uni-Morisita | 0.54 ( 0.26 - 1.1 ) | 0.086 | 0.574 | 0.97 ( 0.31 - 3.04 ) | 0.96 | 0.518 |
|  | Uni-node | 1.06 ( 0.51 - 2.21 ) | 0.88 | 0.508 | 2.35 ( 0.82 - 6.74 ) | 0.1 | 0.607 |
|  | Uni-size | 2.28 ( 1.26 - 4.15 ) | 0.0062 | 0.639 | 2.37 ( 0.83 - 6.79 ) | 0.11 | 0.627 |
|  | Uni- grade | 1.04 ( 0.25 - 4.35 ) | 0.96 | 0.506 | 103390394.03 ( 0 - Inf ) | 0.1 | 0.583 |
|  | Multi-Morisita | 0.52 ( 0.25 - 1.07 ) | 0.077 | 0.665 | 0.64 ( 0.2 - 2.06 ) | 0.45 | 0.696 |
|  | Multi-node | 0.78 ( 0.37 - 1.65 ) | 0.51 |  | 1.75 ( 0.61 - 5.02 ) | 0.3 |  |
|  | Multi-size | 2.5 ( 1.31 - 4.75 ) | 0.0054 |  | 2.11 ( 0.61 - 7.28 ) | 0.24 |  |
|  | Multi- grade | 1.55 ( 0.36 - 6.75 ) | 0.56 |  | 89806379.13 ( 0 - Inf ) | 1 |  |
| LumB  (263) | Uni-Morisita | 1.41 ( 0.71 - 2.8 ) | 0.33 | 0.55 | 0.59 ( 0.24 - 1.49 ) | 0.26 | 0.569 |
|  | Uni-node | 2.27 ( 1.18 - 4.37 ) | 0.012 | 0.597 | 3.55 ( 1.04 - 12.07 ) | 0.03 | 0.61 |
|  | Uni-size | 1.34 ( 0.7 - 2.55 ) | 0.37 | 0.532 | 1.77 ( 0.78 - 3.99 ) | 0.17 | 0.595 |
|  | Uni- grade | 1.08 ( 0.61 - 1.93 ) | 0.79 | 0.515 | 1.58 ( 0.66 - 3.79 ) | 0.3 | 0.588 |
|  | Multi- Morisita | 1.31 ( 0.65 - 2.64 ) | 0.44 | 0.612 | 0.61 ( 0.24 - 1.55 ) | 0.3 | 0.678 |
|  | Multi-node | 1.94 ( 0.96 - 3.92 ) | 0.066 |  | 2.85 ( 0.79 - 10.24 ) | 0.11 |  |
|  | Multi-size | 1.17 ( 0.59 - 2.33 ) | 0.66 |  | 1.42 ( 0.59 - 3.42 ) | 0.43 |  |
|  | Multi- grade | 0.96 ( 0.53 - 1.74 ) | 0.9 |  | 1.28 ( 0.53 - 3.04 ) | 0.58 |  |

**Table S3. Groups of multivariate Cox proportional hazard analysis of the Morisita index and treatment, known clinical or other variables in HER2-amplified, HER2-amplified & CT-treated, and HER2-amplified & RT-treated patients.** Significant p-values are bolded.

| HER2+ (128 patients) | | | |
| --- | --- | --- | --- |
|  | HR(CI) | *p*-value | Conc |
| **Morisita** | 0.28(0.15-0.54) | **1.3x10^-4^** | 0.724 |
| CT | 2.3(1.03-5.13) | **0.042** |  |
| RT | 1.7(0.8-3.64) | 0.17 |  |
| HT | 0.84(0.39-1.82) | 0.66 |  |
| **Morisita** | 0.26(0.11-0.61) | **0.0054** | 0.713 |
| lym | 1.63(0.71-3.74) | 0.66 |  |
| ER | 0.52(0.27-1.03) | 0.067 |  |
| TP53 | 1.27(0.63-2.57) | 0.38 |  |
| **Morisita** | 0.26(0.13-0.5) | **7x10^-5^** | 0.752 |
| grade | 1.5(0.67-3.35) | 0.32 |  |
| node | 3.42(1.5-7.79) | **0.0358** |  |
| size | 2.23(1.28-3.89) | **0.0045** |  |
| HER2+, CT-treated (58 patients) | | | |
|  | HR(CI) | *p*-value | Conc |
| **Morisita** | 0.22(0.09-0.53) | **7.5x10^-4^** | 0.746 |
| grade | 0.97(0.34-2.73) | 0.95 |  |
| node | 2.66(0.62-11.47) | 0.19 |  |
| size | 1.94(0.93-4.05) | 0.079 |  |
| **Morisita** | 0.13(0.04-0.42) | **6.5x10^-4^** | 0.729 |
| lym | 2.33(0.79-6.87) | 0.12 |  |
| ER | 0.68(0.22-2.11) | 0.51 |  |
| TP53 | 0.41(0.16-1.05) | 0.063 |  |
| HER2+, RT-treated (90 patients) | | | |
|  | HR(CI) | *p*-value | Conc |
| **Morisita** | 0.22(0.1-0.49) | **2.1x10^-4^** | 0.727 |
| grade | 1.54(0.63-3.75) | 0.34 |  |
| node | 1.83(0.78-4.33) | 0.17 |  |
| size | 1.9(1.05-3.41) | **0.033** |  |
| **Morisita** | 0.15(0.05-0.4) | **1.7x10^-4^** | 0.741 |
| lym | 2.44(0.99-6.02) | 0.052 |  |
| ER | 0.36(0.17-0.8) | **0.012** |  |
| TP53 | 1.02(0.45-2.32) | 0.96 |  |
| HER2+, not HT-treated (60 patients) | | | |
|  | HR(CI) | *p*-value | Conc |
| **Morisita** | 0.19(0.07-0.5) | **9.4x10^-4^** | 0.737 |
| grade | 1.22(0.33-4.47) | 0.76 |  |
| node | 1.88(0.62-5.7) | 0.27 |  |
| size | 1.96(0.98-3.92) | 0.058 |  |
| **Morisita** | 0.11(0.03-0.43) | **0.0014** | 0.754 |
| lym | 1.56(0.56-4.28) | 0.39 |  |
| ER | 1.9(0.46-7.77) | 0.37 |  |
| TP53 | 0.33(0.12-0.89) | **0.029** |  |
