# Supplementary material for: An ecological measure of immune-cancer colocalization as a prognostic factor for breast cancer
Source: Breast Cancer Res. 2015 Sep 22;17(1):131. doi: 10.1186/s13058-015-0638-4 (PMC4579663; doi:10.1186/s13058-015-0638-4)
Supplement: Additional file 3: — Sweave file. R code used for reproducing all results presented in the paper. (PDF 600 kb) [file 13058_2015_638_MOESM3_ESM.pdf]

Supplementary materials, R code and data for – Co-localisation –

Contents

1 Preparation **2**
1.1 Load data and functions 2
1.2 Clinical parameters of the samples 2
1.3 Clinical parameters and co-localisation indices 3

2 Association between cancer-lymphocyte co-localisation and breast cancer survival **4**
2.1 Prognostic value of spatial correlation based on Voronoi tessellation 4
2.2 Comparisons of prognostic value for all four variables 6
2.3 Correlation among all four variables 7
2.4 Additional value to standard clinical parameters 9
2.5 Robustness of Cox model 17
2.6 Clinical parameters and correlation with the Morisita index 18
2.7 Morisita index in addition to clinicopathologic variables 22

3 Differences in prognostic value of Morisita index according to breast cancer subtypes **24**
3.1 Breast cancer subtypes 24
3.2 Morisita index in LumA 25
3.3 Morisita index in LumB and Basal 26
3.4 Morisita index and treatments in LumA 28
3.5 Robustness of Cox model in LumA 32
3.6 Morisita index in Her2+ 33
3.7 Robustness of Cox model in Her2+ 34
3.8 Morisita index and clinical parameters in Her2+ 35
3.9 Morisita index and treatments in Her2+ 37
3.10 Compare with lymphocytic infiltration in Her2+ 42
3.11 Lym interaction with Morisita in ER- 44

4 Generation of data step-by-step **47**
4.1 Image analysis data 47
4.2 Voronoi tessellation 47
4.3 Square tessellation 47
4.4 Computing correlation and Morisita index 48

5 Session Info **49**

1 Preparation

1.1 Load data and functions

To reproduce our result, first source the R functions needed for analysis and plotting and load the data for 1,026 breast cancer samples, which is available as part of the paper supplement and online ("http://yuanlab.org").

```
library(survival)
# load data file from local copy or from URL
if (file.exists("./data/DF.rdata")){
        load("./data/DF.rdata")
        load("./data/trait.rdata")
} else {
  load(url("http://yuanlab.org/software/Morisita2014/data/trait.rdata"))
  load(url("http://yuanlab.org/software/Morisita2014/data/DF.rdata"))
}

if (file.exists("functions.R")){
        source("functions.R")
} else {
  source(url("http://yuanlab.org/software/Morisita2014/functions.R"))
}
```

1.2 Clinical parameters of the samples

Each row in this clinical file is a sample/patient from the METABRIC database. Columns indicate: public METABRIC ID (public), image ID (file), 10-year disease-specific survival (S_10year), Pam50 intrinsic subtype classification (Pam50Subtype), IntClust subtypes (IntClustMemb), sample contributing hospital sites (Site), proportions of cancer, stromal cells and lymphocytes (cancer, stromal, lym), pathological scores of lymphocytic infiltration (Lymphocytic.infiltration), lymph node status (node), tumour size (size), clinical grade (grade), er and her2 status from IHC staining, and er status from microarray (ER.Expr), Her2 status from microarray (Her2.SNP6). The Site object describes the contributing hospitals and will be used to annotate samples from two cohorts, as described in the paper.

```
head(trait)

##   file grade node size Pam50Subtype IntClustMemb   S_10year
## 1 1645     3    1    2       Normal            4  99.96667+
## 2 1729     3    0    1         LumA            4  49.46667+
## 3 1646     2    1    1         LumB            3 101.76667+
## 4 1647     2    1    2         LumB            9  57.36667+
## 5 1648     3    1    2         LumB            9  41.36667
## 6 1649     3    0    2         LumB            7   7.80000
##    public Site  er her2 ER.Expr Her2.SNP6    cancer
## 1 MB-0000    2 pos null       +         0 0.7120942
## 2 MB-0002    2 pos null       +         0 0.7633116
## 3 MB-0005    2 pos null       +         0 0.7752416
## 4 MB-0006    2 pos    0       +         0 0.7045304
## 5 MB-0008    2 pos    0       +         0 0.7203789
## 6 MB-0010    2 pos null       +         0 0.7281893
##      stromal       lym TP53 Lymphocyte.infiltration    ct
## 1 0.13327698 0.1546288    0                    mild  null
```

```
## 2 0.02526503 0.2114234    1                    mild  null
## 3 0.03581177 0.1889466    1                    mild OTHER
## 4 0.04042977 0.2550399    0                    mild OTHER
## 5 0.06208759 0.2175335    1                    mild  CAPE
## 6 0.04939248 0.2224182    1                    mild  null
##         rt     ht
## 1       CW TAM/AI
## 2       CW    TAM
## 3     null    TAM
## 4       CW     AI
## 5 CW-NODAL    TAM
## 6       CW    TAM

set2 <- rep(TRUE, nrow(trait))
Site <- list(Site1=grepl(1, trait$Site[set2]), Site2=grepl(2, trait$Site[set2]))
```

The summary statistics of breast tumours in our cohort are given below. We focus on disease-specific survival (DSS) within 10 years from diagnosis.

```
summary(trait$S_10year)

##       time               status
##  Min.   :  0.2667   Min.   :0.0000
##  1st Qu.: 45.5167   1st Qu.:0.0000
##  Median : 72.2000   Median :0.0000
##  Mean   : 75.2533   Mean   :0.1807
##  3rd Qu.:120.0000   3rd Qu.:0.0000
##  Max.   :120.0000   Max.   :1.0000
##  NA's   :3          NA's   :13
```

Now the estimated median follow-up time can be calculated by the reverse Kaplan-Meier method. We invert the censoring index for death to estimate time to loss of follow up.

```
survfit(Surv(trait$S_10year[,1], trait$S_10year[,2]==0) ~ 1)

## Call: survfit(formula = Surv(trait$S_10year[, 1], trait$S_10year[,
##     2] == 0) ~ 1)
##
##    14 observations deleted due to missingness
## records   n.max n.start  events  median 0.95LCL 0.95UCL
##  1012.0  1012.0  1012.0   829.0    88.5    83.5    98.2
```

There are 2 censoring events for DSS, and median DSS (shown earlier) will closely approximate median follow up.

1.3 Clinical parameters and co-localisation indices

The first two columns of DF are cancer-immune correlation and Morisita index obtained from Voronoi tessellation. The 3rd and 4th columns are the same but obtained from square tessellation. We will refer to each of these variables as DF1, DF2... for the sake of simplicity in this document.

```
head(DF)

##           Cor.V Morisita.V     Cor.S Morisita.S
## 1645 0.7522241  0.8938435 0.6374599  0.8411327
```

```
## 1729 0.5617537  0.8259938 0.6379874  0.8442363
## 1646 0.6298261  0.8308742 0.4784415  0.8375827
## 1647 0.6939971  0.8993823 0.7151616  0.8891862
## 1648 0.7176022  0.8919380 0.6737525  0.8530288
## 1649 0.6791014  0.8774062 0.5083210  0.8599050
```

Distribution of DF scores in the two cohorts are visualised for Voronoi.

```
par(mfrow=c(2,2))
hist(DF[Site[[1]],1], xlim=range(DF[,1], na.rm=T), main='Cohort 1', xlab='Spatial Cor (Voronoi
hist(DF[Site[[2]],1], xlim=range(DF[,1], na.rm=T), main='Cohort 2', xlab="Spatial Cor (Voronoi
hist(DF[Site[[1]],2], xlim=range(DF[,2], na.rm=T), main='Cohort 1', xlab='Morisita (Voronoi)')
hist(DF[Site[[2]],2], xlim=range(DF[,2], na.rm=T), main='Cohort 2', xlab="Morisita  (Voronoi)"
```

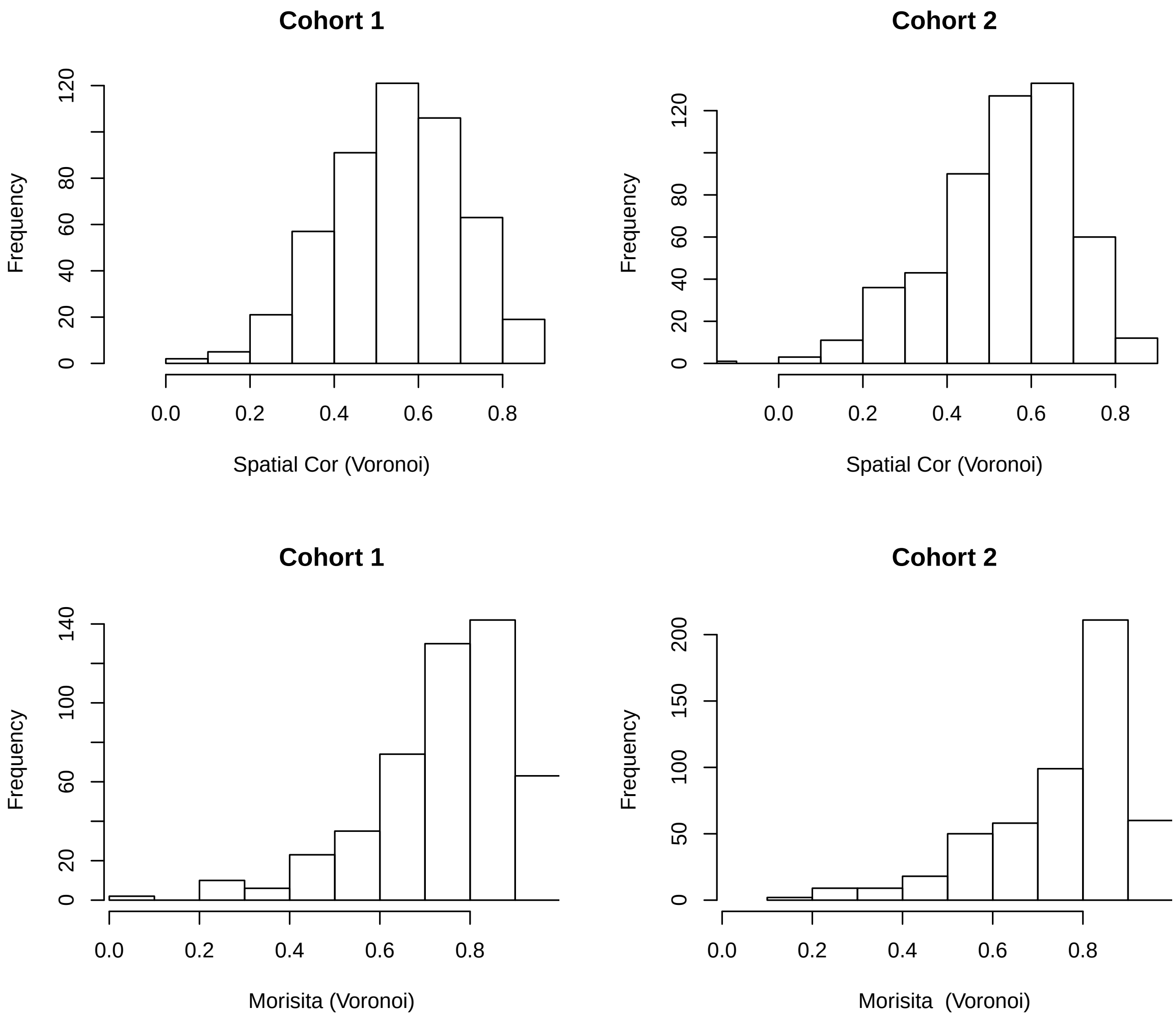

2 Association between cancer-lymphocyte co-localisation and breast cancer survival

2.1 Prognostic value of spatial correlation based on Voronoi tessellation

Using Site 1 as the discovery cohort and Site 2 as the validation cohort, we will test the association between prognosis and a predictive variable. i is set to 1 to refer to the ITL score in the matrix mat.

We search a range of quantiles from 20% to 80% for the optimal cut-off in the discovery cohort at 5% interval (0.20 0.25 0.30 0.35 0.40 0.45 0.50 0.55 0.60 0.65 0.70 0.75 0.80).

```
s <- 1
i <- 1
testrange=seq(0.2,.8,len=13)
library(survival)
p <- sapply(testrange, function(q){
  dat <- data.frame(x=DF[Site[[s]],i]>quantile(DF[Site[[s]],i], q, na.rm=T), S=trait$S_10year[
  fit <- survfit(S ~ x,data=dat)
  test <- survdiff(S ~ x, data=dat, rho=0)
  p.val <- 1 - pchisq(test$chisq, length(test$n) - 1)
  p.val})
```

Now plot the p-values from the log-rank test across different quantiles.

```
plot(testrange, -log(p), pch=19, xlab='Quantile range')
abline(h=-log(0.05), lty=2)
q <- testrange[which.min(p)]
abline(v=q)
```

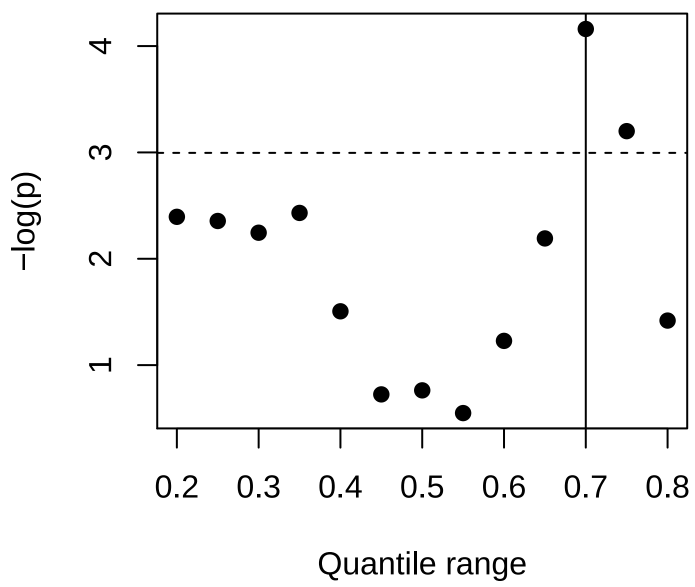


```
th <- quantile(DF[Site[[s]],i], q, na.rm=T)
th_DF1 <- th
```

A cut-off of 0.6378519 at 0.7% quantile is identified as the optimal. Now we test this cut-off in both cohorts using KM curves to illustrate the result.

```
par(mfrow=c(1,2))
for (j in 1:2){
  tmp <- replace.vector(DF[Site[[j]],i]>th_DF1, c(TRUE, FALSE), c('High', 'Low'))
  try( plotSurv(trait$S_10year[Site[[j]],], tmp, fileType='', name=colnames(DF)[i]))
}
```

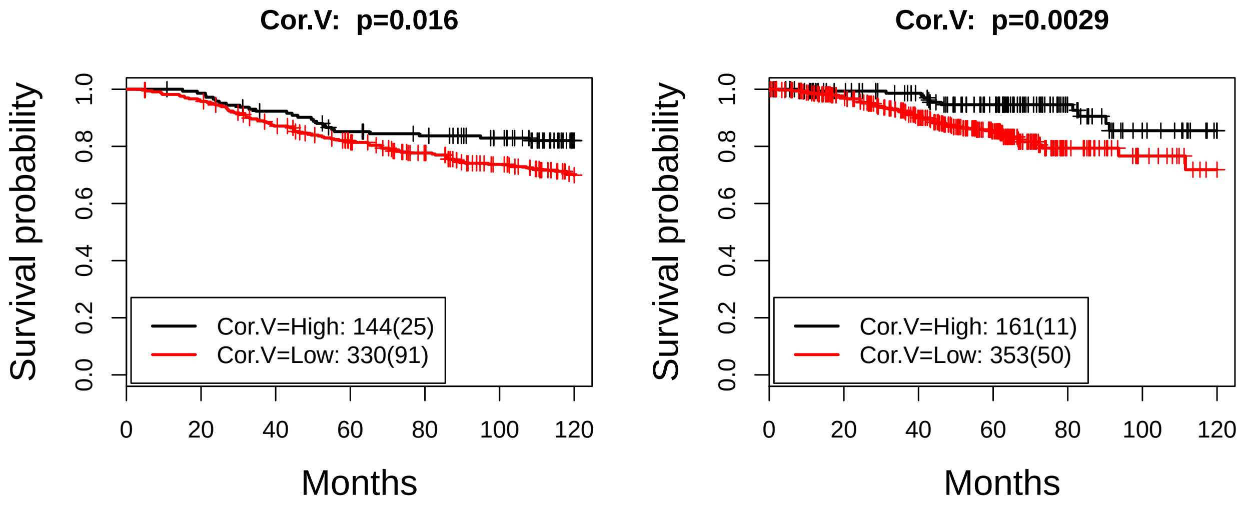

2.2 Comparisons of prognostic value for all four variables

The same test can be performed for all four variables. First, optimise the cut-offs in the discovery cohorts and record them in the Th object. Then these cut-offs are used in producing KM plots in both cohorts.

```
Th <- NULL
par(mfrow=c(3,3))
for (i in 2:4){
p <- sapply(testrange, function(q){
  dat <- data.frame(x=DF[Site[[s]],i]>quantile(DF[Site[[s]],i], q, na.rm=T), S=trait$S_10year[
  fit <- survfit(S ~ x,data=dat)
  test <- survdiff(S ~ x, data=dat, rho=0)
  p.val <- 1 - pchisq(test$chisq, length(test$n) - 1)
  p.val})

plot(testrange, -log(p), pch=19, xlab='Quantile range', main=colnames(DF)[i])
abline(h=-log(0.05), lty=2)
q <- testrange[which.min(p)]
th <- quantile(DF[Site[[s]],i], q, na.rm=T)
abline(v=q)
Th <- c(Th, th)

for (j in 1:2){
  tmp <- replace.vector(DF[Site[[j]],i]>th, c(TRUE, FALSE), c('High', 'Low'))
  try( plotSurv(trait$S_10year[Site[[j]],], tmp, fileType='', name=colnames(DF)[i]))
}
}
```

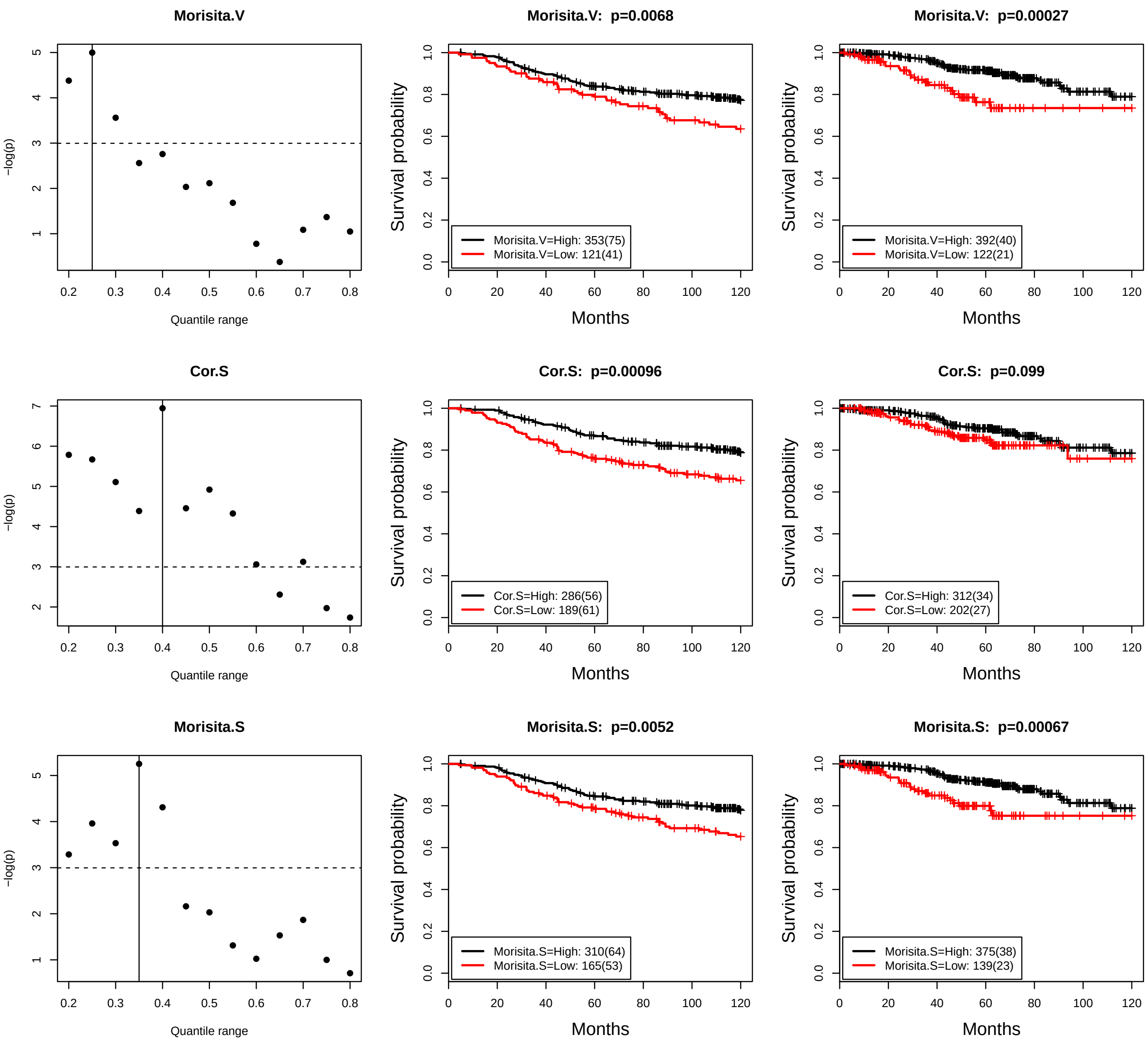

The cutoff points are 0.6677343, 0.4884236, 0.6940734, respectively.

2.3 Correlation among all four variables

Correlation between these variables are high.

```
par(mfrow=c(1,2))
plot(DF[,1], DF[,3], xlab='Voronoi', ylab='Square', main=paste('Correlation, Cor=', signif(cor
legend('bottomright', pch=19, col=1:2, legend=c('Otherwise', 'Breast-cancer death'))
abline(a=0,b=1)
plot(DF[,2], DF[,4], xlab='Voronoi', ylab='Square', main=paste('Morisita, Cor =', signif(cor(D
legend('bottomright', pch=19, col=1:2, legend=c('Otherwise', 'Breast-cancer death'))
abline(a=0,b=1)
```

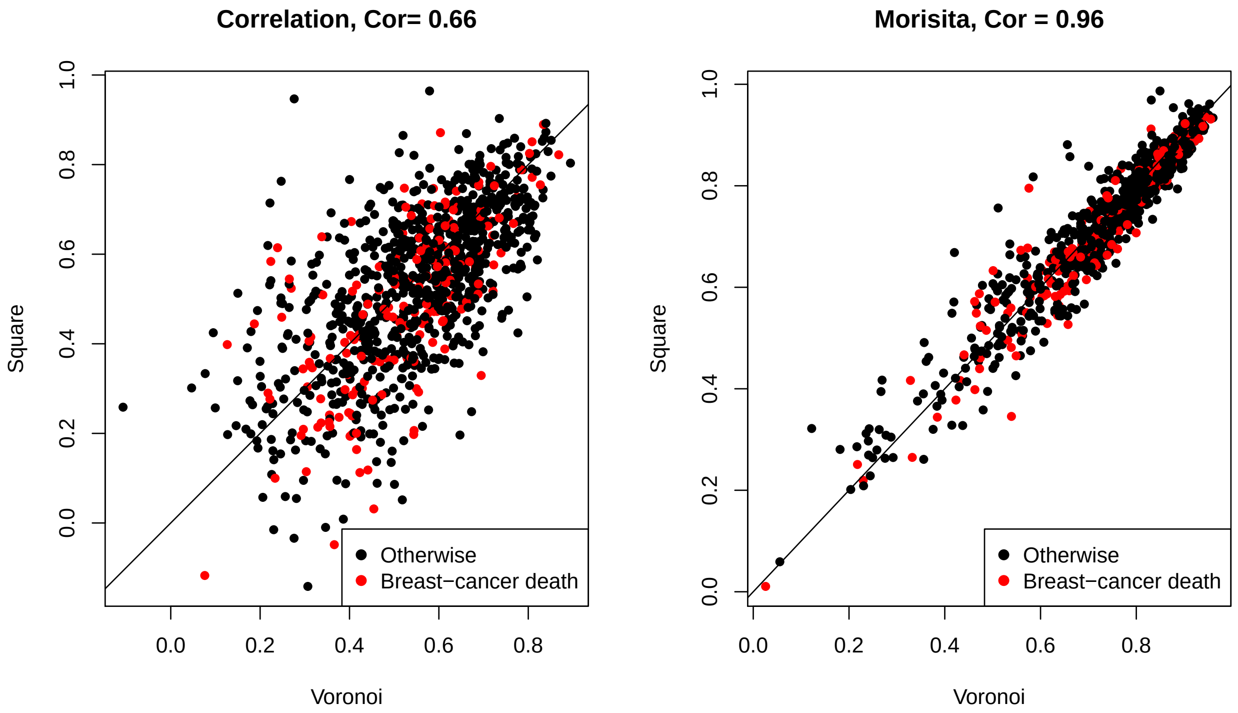

And correlation between correlation and Morisita with the same tessellation:

```
cor.test(DF[,1], DF[,2], use='complete')

##
##  Pearson's product-moment correlation
##
## data:  DF[, 1] and DF[, 2]
## t = 58.6005, df = 999, p-value < 2.2e-16
## alternative hypothesis: true correlation is not equal to 0
## 95 percent confidence interval:
##  0.8653708 0.8933810
## sample estimates:
##       cor
## 0.8801397

  cor.test(DF[,3], DF[,4], use='complete')

##
##  Pearson's product-moment correlation
##
## data:  DF[, 3] and DF[, 4]
## t = 37.8765, df = 1000, p-value < 2.2e-16
## alternative hypothesis: true correlation is not equal to 0
## 95 percent confidence interval:
##  0.7409257 0.7919161
## sample estimates:
##      cor
## 0.767633
```

2.4 Additional value to standard clinical parameters

Additional value of DFs to node and size in our samples is demonstrated here using univariate and multivariate Cox regression analysis in Cohort 1. We used the log-rank test result from univariate analysis and Wald test results from multivariate analysis.

```
x <- DF[,1]>th_DF1
print("Cohort 1")

## [1] "Cohort 1"

set2 <- Site[[1]]
summary(coxph(trait$S_10year[set2,]~x[set2]))

## Call:
## coxph(formula = trait$S_10year[set2, ] ~ x[set2])
##
##   n= 474, number of events= 116
##    (36 observations deleted due to missingness)
##
##                  coef exp(coef) se(coef)      z Pr(>|z|)
## x[set2]TRUE -0.5397    0.5829   0.2259 -2.389   0.0169 *
## ---
## Signif. codes:
## 0 '***' 0.001 '**' 0.01 '*' 0.05 '.' 0.1 ' ' 1
##
##             exp(coef) exp(-coef) lower .95 upper .95
## x[set2]TRUE    0.5829      1.715    0.3744    0.9076
##
## Concordance= 0.549  (se = 0.022 )
## Rsquare= 0.013   (max possible= 0.946 )
## Likelihood ratio test= 6.28  on 1 df,   p=0.0122
## Wald test            = 5.71  on 1 df,   p=0.01688
## Score (logrank) test = 5.85  on 1 df,   p=0.01559

summary(coxph(S_10year[set2,]~node[set2], data=trait))

## Call:
## coxph(formula = S_10year[set2, ] ~ node[set2], data = trait)
##
##   n= 498, number of events= 122
##    (12 observations deleted due to missingness)
##
##               coef exp(coef) se(coef)    z Pr(>|z|)
## node[set2] 0.8604    2.3641   0.1942 4.43 9.43e-06 ***
## ---
## Signif. codes:
## 0 '***' 0.001 '**' 0.01 '*' 0.05 '.' 0.1 ' ' 1
##
##            exp(coef) exp(-coef) lower .95 upper .95
## node[set2]     2.364      0.423     1.616     3.459
##
## Concordance= 0.607  (se = 0.023 )
## Rsquare= 0.042   (max possible= 0.947 )
## Likelihood ratio test= 21.17  on 1 df,   p=4.201e-06
```

```
## Wald test             = 19.62  on 1 df,   p=9.435e-06
## Score (logrank) test = 20.86  on 1 df,   p=4.943e-06
```

```
summary(coxph(trait$S_10year[set2,]~size[set2], data=trait))
```

```
## Call:
## coxph(formula = trait$S_10year[set2, ] ~ size[set2], data = trait)
##
##   n= 498, number of events= 122
##    (12 observations deleted due to missingness)
##
##              coef exp(coef) se(coef)     z Pr(>|z|)
## size[set2] 0.8162    2.2618   0.1661 4.914 8.91e-07 ***
## ---
## Signif. codes:
## 0 '***' 0.001 '**' 0.01 '*' 0.05 '.' 0.1 ' ' 1
##
##            exp(coef) exp(-coef) lower .95 upper .95
## size[set2]     2.262     0.4421     1.633     3.132
##
## Concordance= 0.612  (se = 0.023 )
## Rsquare= 0.048   (max possible= 0.947 )
## Likelihood ratio test= 24.38  on 1 df,   p=7.918e-07
## Wald test             = 24.15  on 1 df,   p=8.91e-07
## Score (logrank) test = 24.53  on 1 df,   p=7.329e-07
```

```
summary(coxph(trait$S_10year[set2,]~grade[set2], data=trait))
```

```
## Call:
## coxph(formula = trait$S_10year[set2, ] ~ grade[set2], data = trait)
##
##   n= 485, number of events= 120
##    (25 observations deleted due to missingness)
##
##               coef exp(coef) se(coef)     z Pr(>|z|)
## grade[set2] 0.7810    2.1837   0.1635 4.777 1.78e-06 ***
## ---
## Signif. codes:
## 0 '***' 0.001 '**' 0.01 '*' 0.05 '.' 0.1 ' ' 1
##
##             exp(coef) exp(-coef) lower .95 upper .95
## grade[set2]     2.184     0.4579     1.585     3.008
##
## Concordance= 0.627  (se = 0.024 )
## Rsquare= 0.055   (max possible= 0.948 )
## Likelihood ratio test= 27.41  on 1 df,   p=1.648e-07
## Wald test             = 22.82  on 1 df,   p=1.776e-06
## Score (logrank) test = 24.3  on 1 df,   p=8.238e-07
```

```
summary(coxph(trait$S_10year[set2,]~x[set2]+trait$node[set2]+trait$size[set2]+trait$grade[set2[illegible]
```

```
## Call:
## coxph(formula = trait$S_10year[set2, ] ~ x[set2] + trait$node[set2] +
##     trait$size[set2] + trait$grade[set2])
```

```
##
##   n= 461, number of events= 114
##    (49 observations deleted due to missingness)
##
##                        coef exp(coef) se(coef)      z
## x[set2]TRUE        -0.6547    0.5196   0.2335 -2.804
## trait$node[set2]    0.5951    1.8132   0.2055  2.896
## trait$size[set2]    0.6551    1.9254   0.1770  3.701
## trait$grade[set2]   0.6135    1.8468   0.1677  3.658
##                    Pr(>|z|)
## x[set2]TRUE        0.005053 **
## trait$node[set2]   0.003783 **
## trait$size[set2]   0.000214 ***
## trait$grade[set2]  0.000254 ***
## ---
## Signif. codes:
## 0 '***' 0.001 '**' 0.01 '*' 0.05 '.' 0.1 ' ' 1
##
##                    exp(coef) exp(-coef) lower .95 upper .95
## x[set2]TRUE           0.5196     1.9246    0.3288    0.8212
## trait$node[set2]      1.8132     0.5515    1.2120    2.7125
## trait$size[set2]      1.9254     0.5194    1.3610    2.7237
## trait$grade[set2]     1.8468     0.5415    1.3295    2.5655
##
## Concordance= 0.702  (se = 0.027 )
## Rsquare= 0.12   (max possible= 0.947 )
## Likelihood ratio test= 58.83  on 4 df,   p=5.1e-12
## Wald test            = 53.67  on 4 df,   p=6.178e-11
## Score (logrank) test = 56.43  on 4 df,   p=1.633e-11
```

Similarly for the Cohort 2, DF1 independently predicts DSS in addition to node, grade and size.

```
set2 <- Site[[2]]
print("Cohort 2")

## [1] "Cohort 2"

summary(coxph(trait$S_10year[set2,]~x[set2]))

## Call:
## coxph(formula = trait$S_10year[set2, ] ~ x[set2])
##
##   n= 514, number of events= 61
##    (2 observations deleted due to missingness)
##
##                  coef exp(coef) se(coef)     z Pr(>|z|)
## x[set2]TRUE -0.9628    0.3818   0.3354 -2.87   0.0041 **
## ---
## Signif. codes:
## 0 '***' 0.001 '**' 0.01 '*' 0.05 '.' 0.1 ' ' 1
##
##             exp(coef) exp(-coef) lower .95 upper .95
## x[set2]TRUE    0.3818      2.619    0.1978    0.7369
##
```

```
## Concordance= 0.597  (se = 0.032 )
## Rsquare= 0.019   (max possible= 0.742 )
## Likelihood ratio test= 9.88  on 1 df,   p=0.001671
## Wald test            = 8.24  on 1 df,   p=0.004101
## Score (logrank) test = 8.86  on 1 df,   p=0.002909

summary(coxph(S_10year[set2,]~node[set2], data=trait))

## Call:
## coxph(formula = S_10year[set2, ] ~ node[set2], data = trait)
##
##   n= 509, number of events= 60
##    (7 observations deleted due to missingness)
##
##              coef exp(coef) se(coef)     z Pr(>|z|)
## node[set2] 1.2664    3.5481   0.3136 4.038 5.39e-05 ***
## ---
## Signif. codes:
## 0 '***' 0.001 '**' 0.01 '*' 0.05 '.' 0.1 ' ' 1
##
##            exp(coef) exp(-coef) lower .95 upper .95
## node[set2]     3.548     0.2818     1.919     6.561
##
## Concordance= 0.642  (se = 0.034 )
## Rsquare= 0.038   (max possible= 0.739 )
## Likelihood ratio test= 19.78  on 1 df,   p=8.682e-06
## Wald test            = 16.31  on 1 df,   p=5.392e-05
## Score (logrank) test = 18.59  on 1 df,   p=1.62e-05

summary(coxph(trait$S_10year[set2,]~size[set2], data=trait))

## Call:
## coxph(formula = trait$S_10year[set2, ] ~ size[set2], data = trait)
##
##   n= 511, number of events= 61
##    (5 observations deleted due to missingness)
##
##              coef exp(coef) se(coef)     z Pr(>|z|)
## size[set2] 0.8832    2.4187   0.2212 3.992 6.55e-05 ***
## ---
## Signif. codes:
## 0 '***' 0.001 '**' 0.01 '*' 0.05 '.' 0.1 ' ' 1
##
##            exp(coef) exp(-coef) lower .95 upper .95
## size[set2]     2.419     0.4134     1.568     3.732
##
## Concordance= 0.643  (se = 0.034 )
## Rsquare= 0.031   (max possible= 0.743 )
## Likelihood ratio test= 15.95  on 1 df,   p=6.504e-05
## Wald test            = 15.94  on 1 df,   p=6.549e-05
## Score (logrank) test = 16.06  on 1 df,   p=6.123e-05

summary(coxph(trait$S_10year[set2,]~grade[set2], data=trait))
```

```
## Call:
## coxph(formula = trait$S_10year[set2, ] ~ grade[set2], data = trait)
##
##   n= 488, number of events= 61
##    (28 observations deleted due to missingness)
##
##                coef exp(coef) se(coef)     z Pr(>|z|)
## grade[set2] 1.0137    2.7557   0.2599 3.899 9.64e-05 ***
## ---
## Signif. codes:
## 0 '***' 0.001 '**' 0.01 '*' 0.05 '.' 0.1 ' ' 1
##
##             exp(coef) exp(-coef) lower .95 upper .95
## grade[set2]     2.756     0.3629     1.656     4.587
##
## Concordance= 0.646  (se = 0.036 )
## Rsquare= 0.04   (max possible= 0.755 )
## Likelihood ratio test= 19.93  on 1 df,   p=8.041e-06
## Wald test            = 15.21  on 1 df,   p=9.64e-05
## Score (logrank) test = 16.84  on 1 df,   p=4.066e-05
```

```
summary(coxph(trait$S_10year[set2,]~x[set2]+trait$node[set2]+trait$size[set2]+trait$grade[set2
```

```
## Call:
## coxph(formula = trait$S_10year[set2, ] ~ x[set2] + trait$node[set2] +
##     trait$size[set2] + trait$grade[set2])
##
##   n= 483, number of events= 60
##    (33 observations deleted due to missingness)
##
##                      coef exp(coef) se(coef)      z
## x[set2]TRUE       -1.0037    0.3665   0.3504 -2.864
## trait$node[set2]   0.8118    2.2519   0.3258  2.492
## trait$size[set2]   0.6721    1.9583   0.2463  2.729
## trait$grade[set2]  0.8921    2.4402   0.2636  3.384
##                   Pr(>|z|)
## x[set2]TRUE       0.004178 **
## trait$node[set2]  0.012714 *
## trait$size[set2]  0.006361 **
## trait$grade[set2] 0.000714 ***
## ---
## Signif. codes:
## 0 '***' 0.001 '**' 0.01 '*' 0.05 '.' 0.1 ' ' 1
##
##                   exp(coef) exp(-coef) lower .95 upper .95
## x[set2]TRUE          0.3665     2.7283    0.1844    0.7284
## trait$node[set2]     2.2519     0.4441    1.1891    4.2644
## trait$size[set2]     1.9583     0.5107    1.2084    3.1734
## trait$grade[set2]    2.4402     0.4098    1.4556    4.0909
##
## Concordance= 0.756  (se = 0.04 )
## Rsquare= 0.096   (max possible= 0.753 )
## Likelihood ratio test= 49  on 4 df,   p=5.851e-10
## Wald test            = 39.92  on 4 df,   p=4.507e-08
```

```
## Score (logrank) test = 43.24  on 4 df,   p=9.241e-09
```

The same univariate and multivariate analysis applied for the other variables.

```
for (i in 2:4){
        print(colnames(DF)[i])
        x<- DF[,i]>Th[i-1]
        set2 <- Site[[1]]
        print(summary(coxph(trait$S_10year[set2,]~x[set2])))
        print(summary(coxph(trait$S_10year[set2,]~x[set2]+trait$node[set2]+trait$size[set2]+tr
}
```

```
## [1] "Morisita.V"
## Call:
## coxph(formula = trait$S_10year[set2, ] ~ x[set2])
##
##   n= 474, number of events= 116
##    (36 observations deleted due to missingness)
##
##                 coef exp(coef) se(coef)      z Pr(>|z|)
## x[set2]TRUE -0.5204    0.5943   0.1943 -2.679  0.00739 **
## ---
## Signif. codes:
## 0 '***' 0.001 '**' 0.01 '*' 0.05 '.' 0.1 ' ' 1
##
##             exp(coef) exp(-coef) lower .95 upper .95
## x[set2]TRUE    0.5943      1.683    0.4061    0.8696
##
## Concordance= 0.551  (se = 0.02 )
## Rsquare= 0.014   (max possible= 0.946 )
## Likelihood ratio test= 6.76  on 1 df,   p=0.009317
## Wald test            = 7.18  on 1 df,   p=0.007386
## Score (logrank) test = 7.34  on 1 df,   p=0.006744
##
## Call:
## coxph(formula = trait$S_10year[set2, ] ~ x[set2] + trait$node[set2] +
##     trait$size[set2] + trait$grade[set2])
##
##   n= 461, number of events= 114
##    (49 observations deleted due to missingness)
##
##                       coef exp(coef) se(coef)      z
## x[set2]TRUE       -0.4352    0.6472   0.1958 -2.222
## trait$node[set2]   0.5672    1.7633   0.2056  2.759
## trait$size[set2]   0.6575    1.9299   0.1789  3.674
## trait$grade[set2]  0.5952    1.8134   0.1686  3.530
##                   Pr(>|z|)
## x[set2]TRUE       0.026254 *
## trait$node[set2]  0.005794 **
## trait$size[set2]  0.000239 ***
## trait$grade[set2] 0.000415 ***
## ---
## Signif. codes:
```

```
## 0 '***' 0.001 '**' 0.01 '*' 0.05 '.' 0.1 ' ' 1
##
##                    exp(coef) exp(-coef) lower .95 upper .95
## x[set2]TRUE           0.6472     1.5452    0.4409    0.9499
## trait$node[set2]      1.7633     0.5671    1.1786    2.6382
## trait$size[set2]      1.9299     0.5182    1.3590    2.7407
## trait$grade[set2]     1.8134     0.5514    1.3031    2.5235
##
## Concordance= 0.698  (se = 0.027 )
## Rsquare= 0.112   (max possible= 0.947 )
## Likelihood ratio test= 54.68  on 4 df,   p=3.789e-11
## Wald test            = 50.05  on 4 df,   p=3.517e-10
## Score (logrank) test = 52.03  on 4 df,   p=1.362e-10
##
## [1] "Cor.S"
## Call:
## coxph(formula = trait$S_10year[set2, ] ~ x[set2])
##
##   n= 475, number of events= 117
##    (35 observations deleted due to missingness)
##
##                 coef exp(coef) se(coef)      z Pr(>|z|)
## x[set2]TRUE -0.6021    0.5476   0.1851 -3.252  0.00115 **
## ---
## Signif. codes:
## 0 '***' 0.001 '**' 0.01 '*' 0.05 '.' 0.1 ' ' 1
##
##              exp(coef) exp(-coef) lower .95 upper .95
## x[set2]TRUE     0.5476      1.826     0.381    0.7872
##
## Concordance= 0.578  (se = 0.023 )
## Rsquare= 0.022   (max possible= 0.947 )
## Likelihood ratio test= 10.51  on 1 df,   p=0.001188
## Wald test            = 10.58  on 1 df,   p=0.001146
## Score (logrank) test = 10.9  on 1 df,   p=0.0009622
##
## Call:
## coxph(formula = trait$S_10year[set2, ] ~ x[set2] + trait$node[set2] +
##     trait$size[set2] + trait$grade[set2])
##
##   n= 462, number of events= 115
##    (48 observations deleted due to missingness)
##
##                       coef exp(coef) se(coef)      z
## x[set2]TRUE       -0.5450    0.5799   0.1886 -2.890
## trait$node[set2]   0.5550    1.7420   0.2039  2.723
## trait$size[set2]   0.6436    1.9033   0.1802  3.572
## trait$grade[set2]  0.5607    1.7520   0.1682  3.334
##                   Pr(>|z|)
## x[set2]TRUE       0.003854 **
## trait$node[set2]  0.006477 **
## trait$size[set2]  0.000354 ***
## trait$grade[set2] 0.000855 ***
```

```
## ---
## Signif. codes:
## 0 '***' 0.001 '**' 0.01 '*' 0.05 '.' 0.1 ' ' 1
##
##                    exp(coef) exp(-coef) lower .95 upper .95
## x[set2]TRUE           0.5799     1.7245    0.4007    0.8392
## trait$node[set2]      1.7420     0.5740    1.1682    2.5977
## trait$size[set2]      1.9033     0.5254    1.3370    2.7093
## trait$grade[set2]     1.7520     0.5708    1.2600    2.4359
##
## Concordance= 0.697  (se = 0.027 )
## Rsquare= 0.117   (max possible= 0.948 )
## Likelihood ratio test= 57.4  on 4 df,   p=1.019e-11
## Wald test            = 53.24  on 4 df,   p=7.593e-11
## Score (logrank) test = 55.83  on 4 df,   p=2.181e-11
##
## [1] "Morisita.S"
## Call:
## coxph(formula = trait$S_10year[set2, ] ~ x[set2])
##
##   n= 475, number of events= 117
##    (35 observations deleted due to missingness)
##
##                 coef exp(coef) se(coef)      z Pr(>|z|)
## x[set2]TRUE -0.5132    0.5986   0.1858 -2.762  0.00574 **
## ---
## Signif. codes:
## 0 '***' 0.001 '**' 0.01 '*' 0.05 '.' 0.1 ' ' 1
##
##             exp(coef) exp(-coef) lower .95 upper .95
## x[set2]TRUE    0.5986      1.671    0.4159    0.8615
##
## Concordance= 0.56  (se = 0.022 )
## Rsquare= 0.016   (max possible= 0.947 )
## Likelihood ratio test= 7.43  on 1 df,   p=0.00641
## Wald test            = 7.63  on 1 df,   p=0.005738
## Score (logrank) test = 7.8  on 1 df,   p=0.005226
##
## Call:
## coxph(formula = trait$S_10year[set2, ] ~ x[set2] + trait$node[set2] +
##     trait$size[set2] + trait$grade[set2])
##
##   n= 462, number of events= 115
##    (48 observations deleted due to missingness)
##
##                      coef exp(coef) se(coef)      z
## x[set2]TRUE       -0.5263    0.5908   0.1874 -2.809
## trait$node[set2]   0.5754    1.7778   0.2033  2.830
## trait$size[set2]   0.6384    1.8935   0.1778  3.591
## trait$grade[set2]  0.5998    1.8217   0.1679  3.572
##                   Pr(>|z|)
## x[set2]TRUE       0.004975 **
## trait$node[set2]  0.004650 **
```

```
## trait$size[set2]  0.000329 ***
## trait$grade[set2] 0.000354 ***
## ---
## Signif. codes:
## 0 '***' 0.001 '**' 0.01 '*' 0.05 '.' 0.1 ' ' 1
##
##                   exp(coef) exp(-coef) lower .95 upper .95
## x[set2]TRUE          0.5908     1.6927    0.4092     0.853
## trait$node[set2]     1.7778     0.5625    1.1936     2.648
## trait$size[set2]     1.8935     0.5281    1.3364     2.683
## trait$grade[set2]    1.8217     0.5489    1.3109     2.532
##
## Concordance= 0.699  (se = 0.027 )
## Rsquare= 0.116   (max possible= 0.948 )
## Likelihood ratio test= 56.75  on 4 df,   p=1.393e-11
## Wald test            = 52.29  on 4 df,   p=1.202e-10
## Score (logrank) test = 54.5  on 4 df,   p=4.136e-11
```

By modifying the code above, one can swap the discovery with the validation cohort and reproduce our results in the paper.

2.5 Robustness of Cox model

In the paper we then focused on DF4 Morisita index by Square tessellation. To test its robustness, we estimate the univariate hazard ratio on DSS using a Cox proportional hazards model using all samples.

```
Morisita <-DF[,4]>Th[3]
summary(coxph(trait$S_10year ~ Morisita))

## Call:
## coxph(formula = trait$S_10year ~ Morisita)
##
##   n= 989, number of events= 178
##    (37 observations deleted due to missingness)
##
##                  coef exp(coef) se(coef)      z Pr(>|z|)
## MorisitaTRUE -0.6497    0.5222   0.1516 -4.285 1.83e-05 ***
## ---
## Signif. codes:
## 0 '***' 0.001 '**' 0.01 '*' 0.05 '.' 0.1 ' ' 1
##
##              exp(coef) exp(-coef) lower .95 upper .95
## MorisitaTRUE    0.5222      1.915    0.3879    0.7029
##
## Concordance= 0.579  (se = 0.018 )
## Rsquare= 0.018   (max possible= 0.904 )
## Likelihood ratio test= 17.51  on 1 df,   p=2.861e-05
## Wald test            = 18.36  on 1 df,   p=1.83e-05
## Score (logrank) test = 19.01  on 1 df,   p=1.3e-05
```

The hazard ratio for Morisita is 0.52 (lower 95% 0.38, higher 95% 0.70). We also check the robustness of our results using bootstrap analysis. We sampled the data with replacement 1,000 times and repeated the log-rank survival analysis:

```r
n <- length(Morisita)
set.seed(45)
resB1 <- replicate(1000,1-pchisq(
         survdiff(trait$S_10year ~ Morisita,
         subset=sample(1:n, replace=TRUE)
         )$chisq,1))
mean(resB1 < 0.05)

## [1] 0.984
```

This means in 98% our results of univariate analysis stay significant in the perturbed data.

```r
set.seed(45)
resB2 <- replicate(1000, summary(coxph(trait$S_10year ~ Morisita+trait$node+trait$size+trait$g
    subset=sample(1:sum(n), replace=TRUE)))$coef[1,5])
mean(resB2 < 0.05)

## [1] 0.987
```

This means in 99% our results of multivariate analysis stay significant. These demonstrated the stability of the Morisita index as a prognostic marker and prognostic factor in breast cancers.

2.6 Clinical parameters and correlation with the Morisita index

Prognostic values of other known clinical parameters in our cohorts can be visualised with Kaplan-Meier curves.

```r
par(mfrow=c(2,3))
plotSurv(trait$S_10year, trait$size, name='size')
plotSurv(trait$S_10year, trait$node, name='node')
plotSurv(trait$S_10year, trait$grade, name='Grade')
plotSurv(trait$S_10year, trait$ER, name='PAM50')
plotSurv(trait$S_10year, trait$Lymphocyte.infiltration, name='Pathological LI')
plotSurv(trait$S_10year, trait$TP53, name='TP53')
```

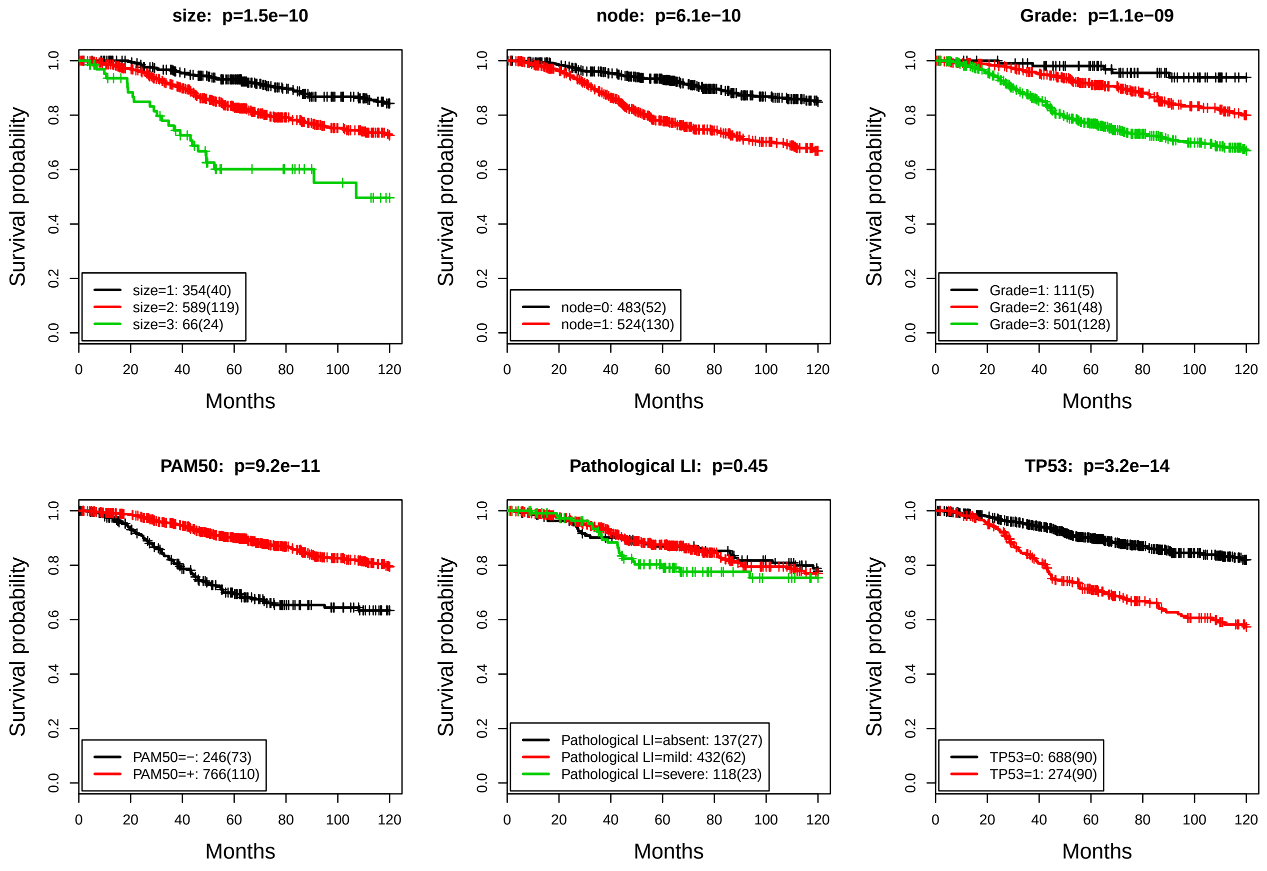

Correlation between the Morisita index and the main clinical parameters can be shown in box plots:

```
set2 <- Site[[1]]
fea <- DF[,4]
feaName <- 'Morisita'
par(mfrow=c(3,3), mar=c(4,3,3,1))
Boxplot(fea[set2]~trait$node[set2], xlab="Node", ylab=feaName)
Boxplot(fea[set2]~trait$size[set2], xlab="Size", ylab=feaName)
Boxplot(fea[set2]~trait$grade[set2], xlab="Grade", ylab=feaName)
lym <- trait$lym[set2] > 0.08
Boxplot(fea[set2]~lym, xlab="Lym", ylab=feaName)
Boxplot(fea[set2]~ as.factor(trait$ER.Expr[set2]), xlab="ER.Expr", ylab=feaName)
Boxplot(fea[set2]~ as.factor(trait$Her2.SNP6[set2]==2), xlab="Her2.SNP6", ylab=feaName)
Boxplot(fea[set2]~ as.factor(trait$TP53[set2]), xlab="TP53 mutation", ylab=feaName)
Pam50Subtype <- trait$Pam50Subtype
Pam50Subtype[Pam50Subtype %in%c('NC', 'Normal')] <- NA
Boxplot(fea[set2]~ as.factor(Pam50Subtype[set2]), xlab="Pam50Subtype", ylab=feaName)
Boxplot(fea[set2]~ as.factor(trait$IntClustMemb[set2]), xlab="IntClustMemb", ylab=feaName)
```

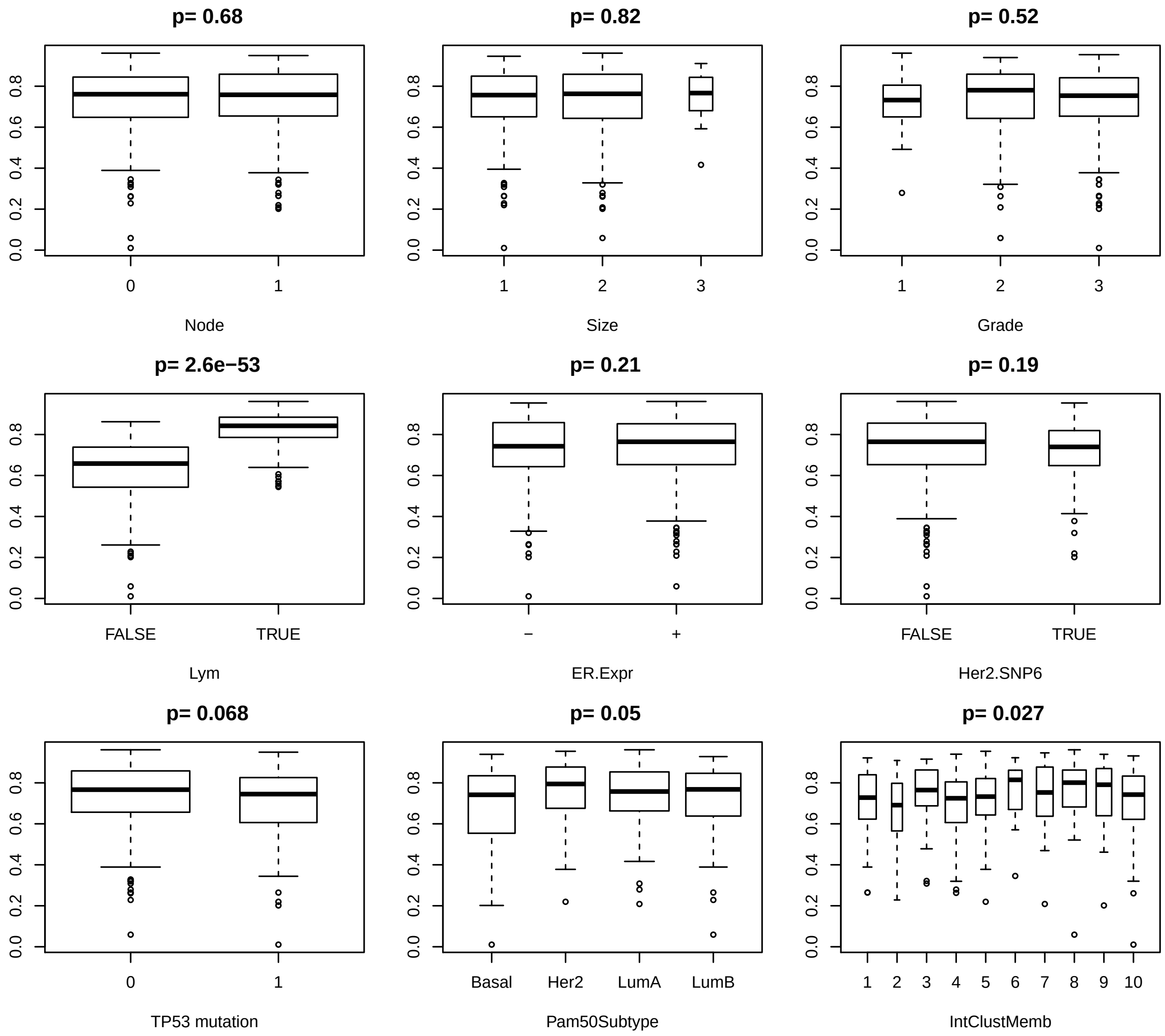


```
set2 <- Site[[2]]
fea <- DF[,4]
feaName <- 'Morisita'
par(mfrow=c(3,3), mar=c(4,3,3,1))
Boxplot(fea[set2]~trait$node[set2], xlab="Node", ylab=feaName)
Boxplot(fea[set2]~trait$size[set2], xlab="Size", ylab=feaName)
Boxplot(fea[set2]~trait$grade[set2], xlab="Grade", ylab=feaName)
lym <- trait$lym[set2] > 0.08
Boxplot(fea[set2]~lym, xlab="Lym", ylab=feaName)
Boxplot(fea[set2]~ as.factor(trait$ER.Expr[set2]), xlab="ER.Expr", ylab=feaName)
Boxplot(fea[set2]~ as.factor(trait$Her2.SNP6[set2]==2), xlab="Her2.SNP6", ylab=feaName)
Boxplot(fea[set2]~ as.factor(trait$TP53[set2]), xlab="TP53 mutation", ylab=feaName)
Pam50Subtype <- trait$Pam50Subtype
Pam50Subtype[Pam50Subtype %in%c('NC', 'Normal')] <- NA
Boxplot(fea[set2]~ as.factor(Pam50Subtype[set2]), xlab="Pam50Subtype", ylab=feaName)
Boxplot(fea[set2]~ as.factor(trait$IntClustMemb[set2]), xlab="IntClustMemb", ylab=feaName)
```

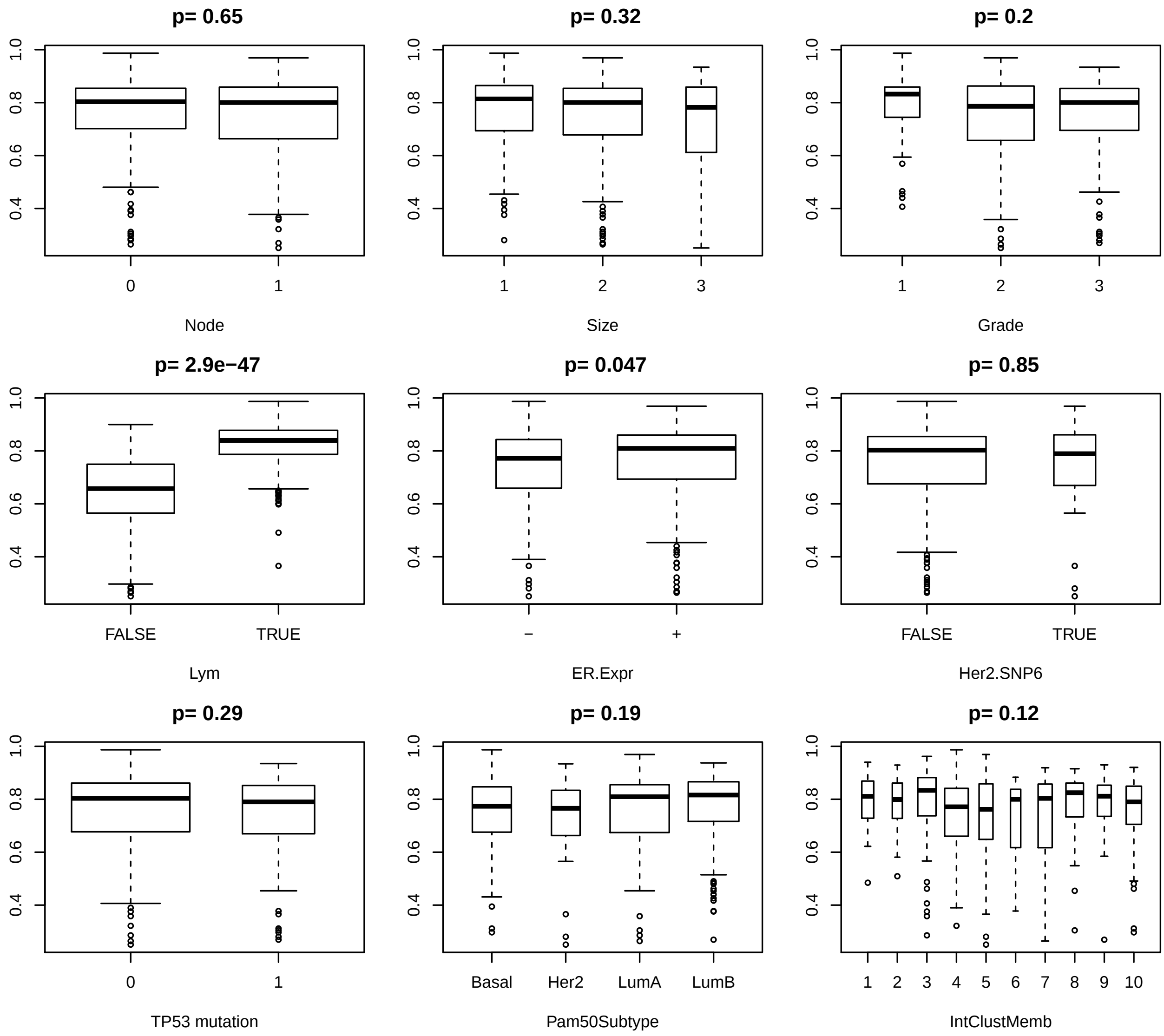

Since the Morisita index is not significantly correlated with grade node and size, but lymphocyte abundance, ER status, TP53 mutations. We then use multivariate Cox regression to test its value with these parameters in two cohorts.

```
set2 <- Site[[1]]
lym<-trait$lym > 0.08
summary(coxph(trait$S_10year[set2,]~Morisita[set2]+lym[set2]+trait$TP53[set2]+trait$ER[set2]))

## Call:
## coxph(formula = trait$S_10year[set2, ] ~ Morisita[set2] + lym[set2] +
##     trait$TP53[set2] + trait$ER[set2])
##
##   n= 465, number of events= 117
##    (45 observations deleted due to missingness)
##
##                          coef exp(coef) se(coef)      z
## Morisita[set2]TRUE -0.5158    0.5970   0.2326 -2.217
## lym[set2]TRUE       0.2540    1.2892   0.2300  1.105
## trait$TP53[set2]    0.8354    2.3058   0.2113  3.954
## trait$ER[set2]+    -0.5573    0.5727   0.2111 -2.640
##                    Pr(>|z|)
## Morisita[set2]TRUE  0.02661 *
## lym[set2]TRUE       0.26936
```

```
## trait$TP53[set2]    7.7e-05 ***
## trait$ER[set2]+     0.00829 **
## ---
## Signif. codes:
## 0 '***' 0.001 '**' 0.01 '*' 0.05 '.' 0.1 ' ' 1
##
##                      exp(coef) exp(-coef) lower .95 upper .95
## Morisita[set2]TRUE      0.5970     1.6749    0.3784    0.9419
## lym[set2]TRUE           1.2892     0.7757    0.8214    2.0232
## trait$TP53[set2]        2.3058     0.4337    1.5239    3.4890
## trait$ER[set2]+         0.5727     1.7460    0.3787    0.8662
##
## Concordance= 0.696  (se = 0.027 )
## Rsquare= 0.099   (max possible= 0.95 )
## Likelihood ratio test= 48.32  on 4 df,   p=8.093e-10
## Wald test            = 50.77  on 4 df,   p=2.493e-10
## Score (logrank) test = 55.96  on 4 df,   p=2.045e-11
```

```
set2 <- Site[[2]]
summary(coxph(trait$S_10year[set2,]~Morisita[set2]+lym[set2]+trait$TP53[set2]+trait$ER[set2]))
```

```
## Call:
## coxph(formula = trait$S_10year[set2, ] ~ Morisita[set2] + lym[set2] +
##     trait$TP53[set2] + trait$ER[set2])
##
##   n= 474, number of events= 58
##    (42 observations deleted due to missingness)
##
##                         coef exp(coef) se(coef)      z
## Morisita[set2]TRUE -1.0132    0.3630   0.3419 -2.963
## lym[set2]TRUE       0.1046    1.1103   0.3432  0.305
## trait$TP53[set2]    0.8419    2.3207   0.2943  2.861
## trait$ER[set2]+    -0.5013    0.6058   0.3124 -1.605
##                    Pr(>|z|)
## Morisita[set2]TRUE  0.00304 **
## lym[set2]TRUE       0.76049
## trait$TP53[set2]    0.00422 **
## trait$ER[set2]+     0.10860
## ---
## Signif. codes:
## 0 '***' 0.001 '**' 0.01 '*' 0.05 '.' 0.1 ' ' 1
##
##                      exp(coef) exp(-coef) lower .95 upper .95
## Morisita[set2]TRUE      0.3630     2.7545    0.1857    0.7096
## lym[set2]TRUE           1.1103     0.9007    0.5667    2.1754
## trait$TP53[set2]        2.3207     0.4309    1.3036    4.1313
## trait$ER[set2]+         0.6058     1.6508    0.3284    1.1174
##
## Concordance= 0.703  (se = 0.039 )
## Rsquare= 0.058   (max possible= 0.748 )
## Likelihood ratio test= 28.56  on 4 df,   p=9.59e-06
## Wald test            = 31.46  on 4 df,   p=2.461e-06
## Score (logrank) test = 34.15  on 4 df,   p=6.942e-07
```

Thus Morisita still holds independent value to these parameters.

2.7 Morisita index in addition to clinicopathologic variables

```
set2 <- rep(TRUE, length(Morisita))
cols <- c('darkred', 'darkblue')
ltys <- c(1,1,4,4)
cols2 <- c('darkblue', 'darkblue','darkred','darkred')
ltys2 <- c(1,4,1,4)
par(mfrow=c(2,3))
plotSurv(trait$S_10year[set2], trait$node[set2], name='Node', col=cols, lty=ltys, mark.time=F)
plotSurv(trait$S_10year[set2], trait$size[set2]==3, name='Size', col=cols, lty=ltys, mark.time
plotSurv(trait$S_10year[set2], trait$grade[set2]==3, name='Grade', col=cols, lty=ltys, mark.ti
plotSurv(trait$S_10year[set2], -1*(Morisita[set2]) -2*(trait$node[set2]!=1), name='Node & Mori
plotSurv(trait$S_10year[set2], -1*(Morisita[set2]) -2*(trait$size[set2]!=3), name='Size & Mori
plotSurv(trait$S_10year[set2], -1*(Morisita[set2]) -2*(trait$grade[set2]!=3), name='Grade & Mo
```

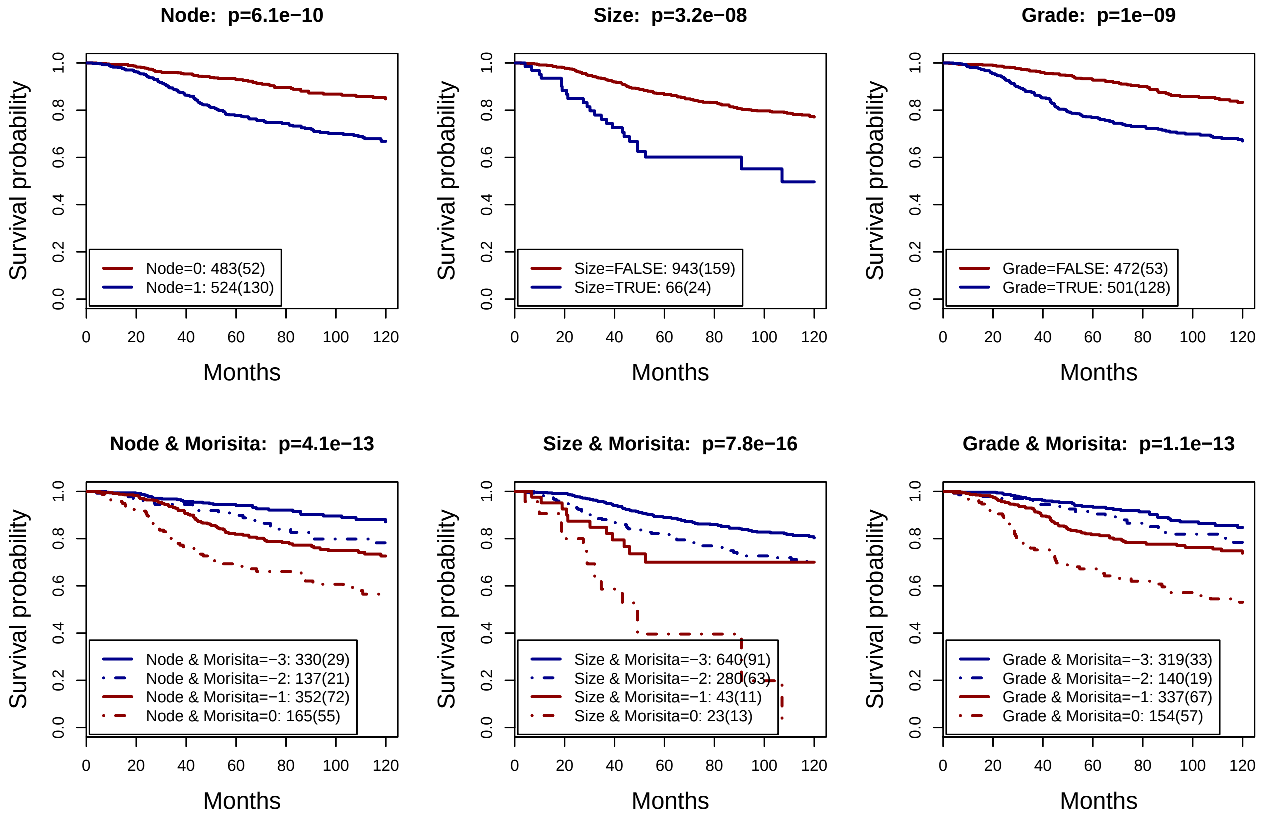

And other parameters for breast cancer including lymphocyte abundance, ER status and TP53 mutation:

```
par(mfrow=c(2,3))
plotSurv(trait$S_10year[set2], trait$lym[set2]<0.08, name='Lym', col=cols, lty=ltys, mark.time
plotSurv(trait$S_10year[set2], trait$ER.Expr[set2]=='-', name='ER', col=cols, lty=ltys, mark.t
plotSurv(trait$S_10year[set2], trait$TP53[set2]==0, name='TP53', col=cols, lty=ltys, mark.time
plotSurv(trait$S_10year[set2], -1*(Morisita[set2]) + -2*(trait$lym[set2]>0.08), name='Lym & Mo
plotSurv(trait$S_10year[set2], -('+'==trait$ER.Expr[set2])*2- 1*( Morisita[set2]), name='ER &
plotSurv(trait$S_10year[set2], -2*trait$TP53[set2] - 1*( Morisita[set2]), name='TP53 & Morisit
```

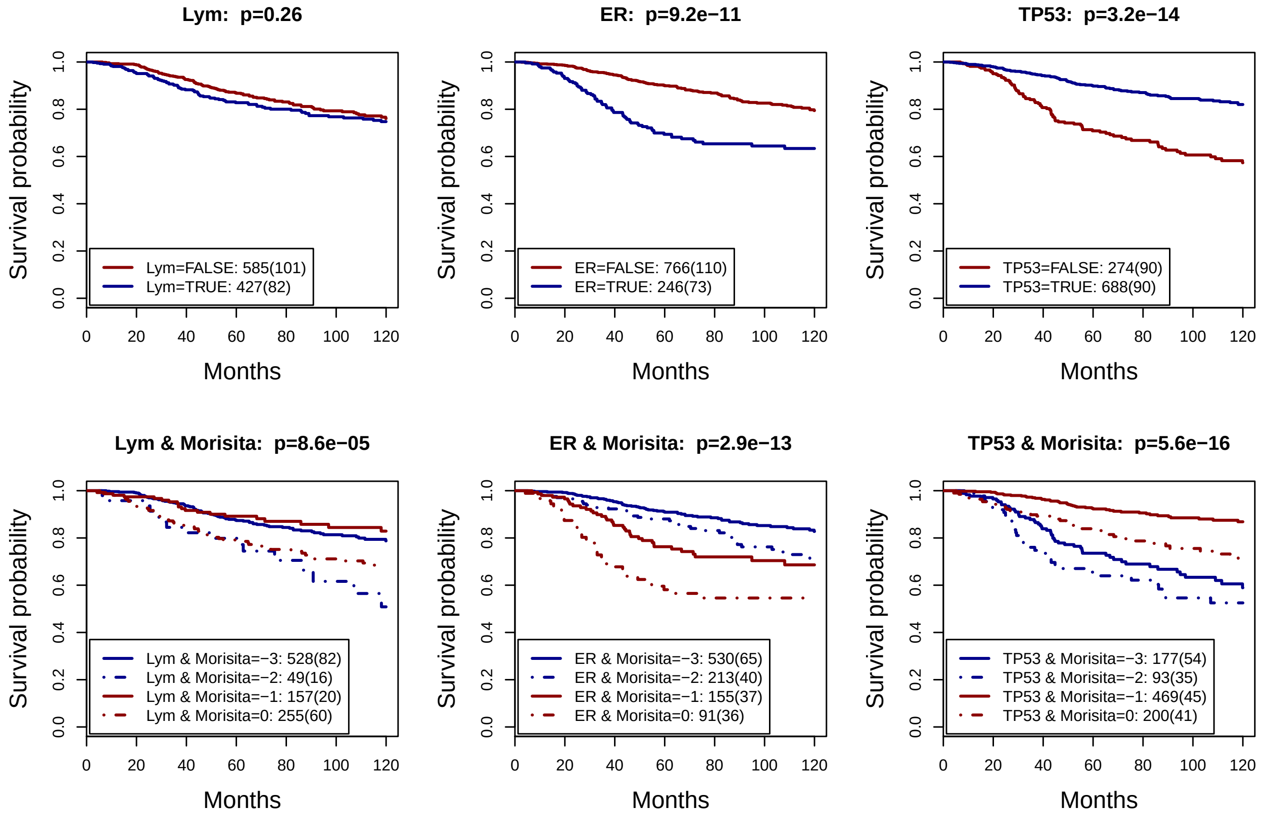

3 Differences in prognostic value of Morisita index according to breast cancer subtypes

3.1 Breast cancer subtypes

We first quickly examine Morisita index using the same threshold for all of the subtypes:

```
par(mfrow=c(3,3))
z <- Morisita
subset=rep(TRUE, length(z))
plotSurv(trait$S_10year[subset], z[subset], name='All')
plotSurv(trait$S_10year[grepl('-', trait$ER.Expr) &subset], z[grepl('-', trait$ER.Expr) &subse
plotSurv(trait$S_10year[!grepl('-', trait$ER.Expr) &subset], z[!grepl('-', trait$ER.Expr) &sub
plotSurv(trait$S_10year[grepl('Basal', trait$Pam50Subtype) &subset], z[ grepl('Basal', trait$P
plotSurv(trait$S_10year[grepl('LumA', trait$Pam50Subtype) &subset], z[ grepl('LumA', trait$Pam
plotSurv(trait$S_10year[grepl('LumB', trait$Pam50Subtype) &subset], z[ grepl('LumB', trait$Pam
plotSurv(trait$S_10year[grepl('Her2', trait$Pam50Subtype) &subset], z[ grepl('Her2', trait$Pam
her2 <- 2==trait$Her2.SNP6
her2[is.na(her2)] <- FALSE
plotSurv(trait$S_10year[her2], z[her2], name='Her2 SNP6')
plotSurv(trait$S_10year[grepl('-', trait$ER.Expr) & !her2] , z[grepl('-', trait$ER.Expr) & !he
```

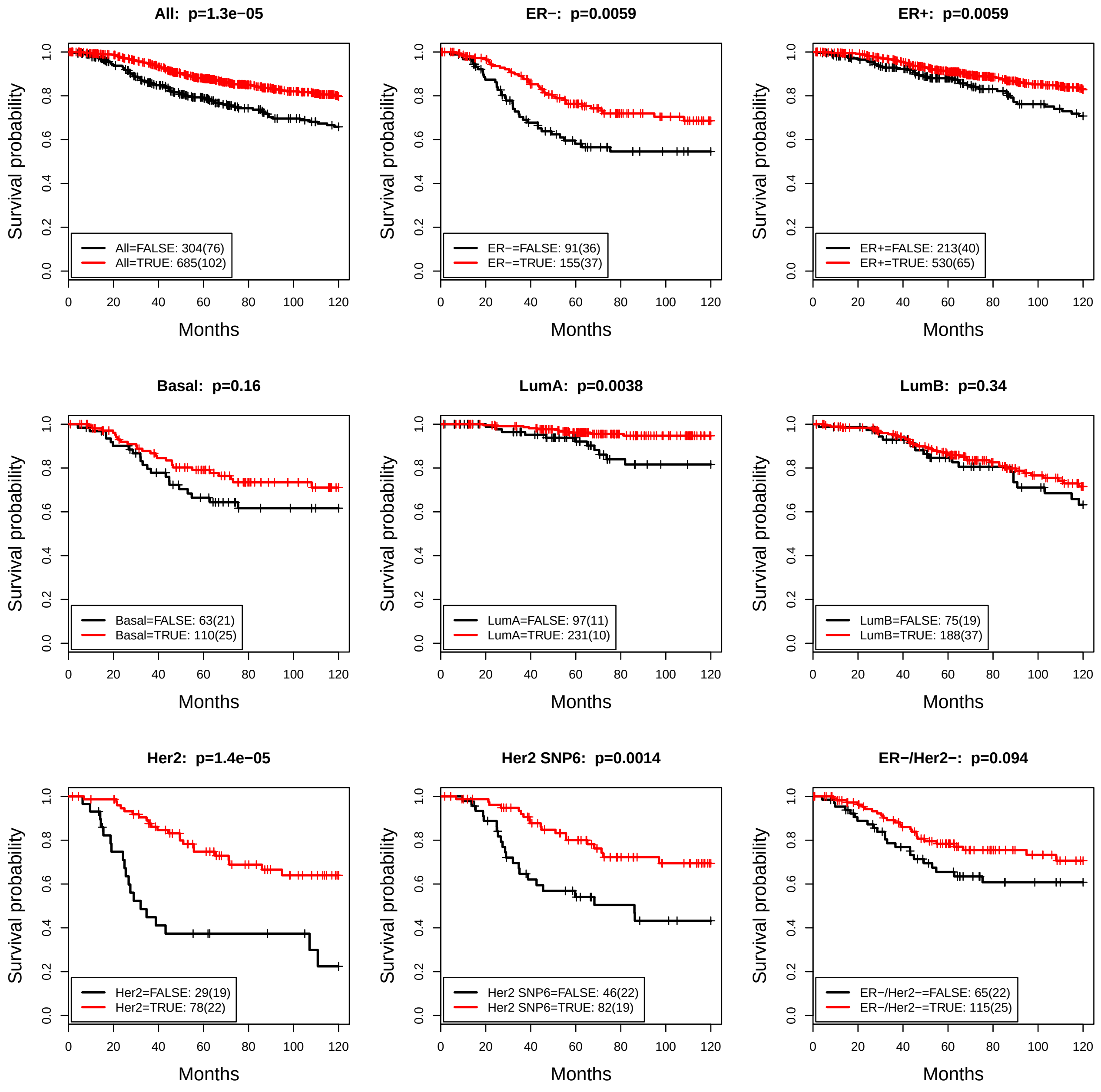

3.2 Morisita index in LumA

First the optimal cut-off was selected from the discovery cohort.

```
set2 <- grepl('LumA', trait$Pam50Subtype) & Site[[1]]
s <- 1
testrange=seq(0.2,.8,len=13)
library(survival)
p <- sapply(testrange, function(q){
  dat <- data.frame(x=DF[set2,4]>quantile(DF[set2,4], q, na.rm=T), S=trait$S_10year[set2])
  fit <- survfit(S ~ x,data=dat)
  test <- survdiff(S ~ x, data=dat, rho=0)
  p.val <- 1 - pchisq(test$chisq, length(test$n) - 1)
  p.val})
```

Now plot the p-values from the log-rank test across different quantiles.

```r
plot(testrange, -log(p), pch=19, xlab='Quantile range')
abline(h=-log(0.05), lty=2)
q <- testrange[which.min(p)]
abline(v=q)
```

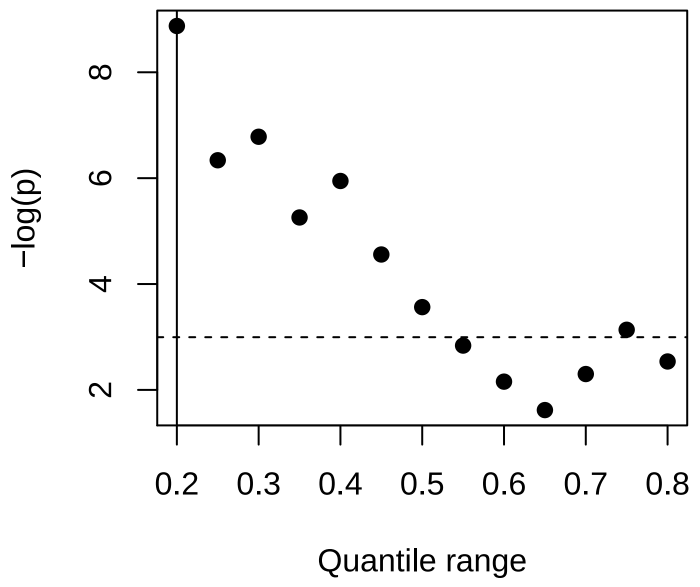


```r
th <- quantile(DF[set2,4], q, na.rm=T)
th_lumA <- th
```

The cutoff selected is 0.639985 at 20 percentile.

3.3 Morisita index in LumB and Basal

```r
set2 <- grepl('LumB', trait$Pam50Subtype) & Site[[1]]
s <- 1
testrange=seq(0.2,.8,len=13)
library(survival)
p <- sapply(testrange, function(q){
  dat <- data.frame(x=DF[set2,4]>quantile(DF[set2,4], q, na.rm=T), S=trait$S_10year[set2])
  fit <- survfit(S ~ x,data=dat)
  test <- survdiff(S ~ x, data=dat, rho=0)
  p.val <- 1 - pchisq(test$chisq, length(test$n) - 1)
  p.val})
```

Now plot the p-values from the log-rank test across different quantiles.

```r
plot(testrange, -log(p), pch=19, xlab='Quantile range')
abline(h=-log(0.05), lty=2)
q <- testrange[which.min(p)]
abline(v=q)
```

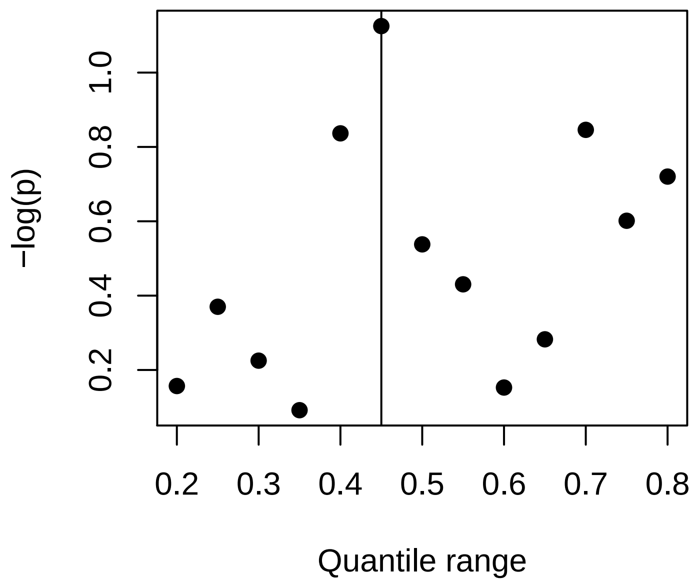


```
th <- quantile(DF[set2,4], q, na.rm=T)
th_lumB <- th
```

The cutoff selected is 0.7439517 at 45 percentile.

```
set2 <- grepl('Basal', trait$Pam50Subtype) & Site[[1]]
s <- 1
testrange=seq(0.2,.8,len=13)
library(survival)
p <- sapply(testrange, function(q){
  dat <- data.frame(x=DF[set2,4]>quantile(DF[set2,4], q, na.rm=T), S=trait$S_10year[set2])
  fit <- survfit(S ~ x,data=dat)
  test <- survdiff(S ~ x, data=dat, rho=0)
  p.val <- 1 - pchisq(test$chisq, length(test$n) - 1)
  p.val})
```

Now plot the p-values from the log-rank test across different quantiles.

```
plot(testrange, -log(p), pch=19, xlab='Quantile range')
abline(h=-log(0.05), lty=2)
q <- testrange[which.min(p)]
abline(v=q)
```

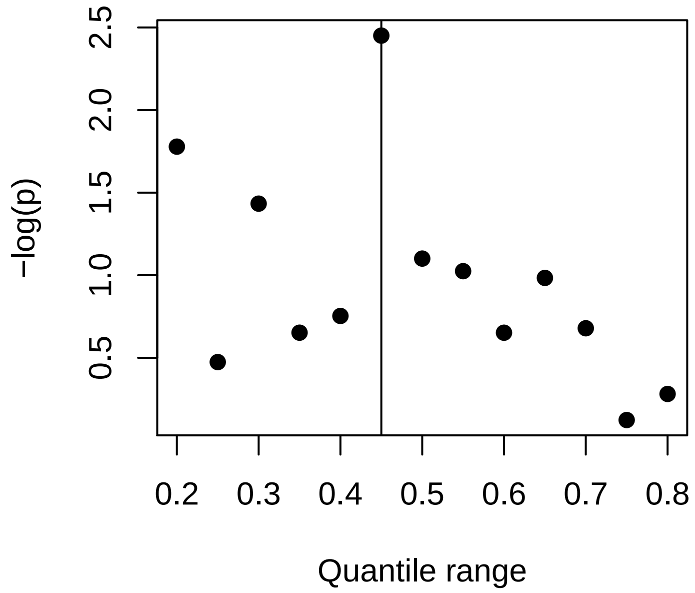


```
th <- quantile(DF[set2,4], q, na.rm=T)
th_basal <- th
```

The cutoff selected is 0.699701 at 45 percentile.

3.4 Morisita index and treatments in LumA

Next, we looked at treatment options on the LumA subtype and whether their effects are different according to the Morisita index. Clinical data on chemotherapy (CT), radiotherapy (RT) and hormonal therapy (HT) are available.

```
z <- DF[,4] > th_lumA
set2 <- grepl('LumA', trait$Pam50Subtype)  & Site[[1]]
ct <- trait$ct!='null'
rt <- trait$rt!='null'
ht <- trait$ht!='null'
par(mfrow=c(2,3))
plotSurv(trait$S_10year[set2], ct[set2], name='CT', col=cols, lty=ltys, mark.time=F)
plotSurv(trait$S_10year[set2], rt[set2], name='RT', col=cols, lty=ltys, mark.time=F)
plotSurv(trait$S_10year[set2], ht[set2], name='HT', col=cols, lty=ltys, mark.time=F)
plotSurv(trait$S_10year[set2], -1*(z[set2]) -2*( ct[set2]), name='CT & Morisita', col=cols2, l
plotSurv(trait$S_10year[set2], -1*(z[set2]) -2*( rt[set2]), name='RT & Morisita', col=cols2, l
plotSurv(trait$S_10year[set2], -1*(z[set2]) -2*( ht[set2]), name='HT & Morisita', col=cols2, l
```

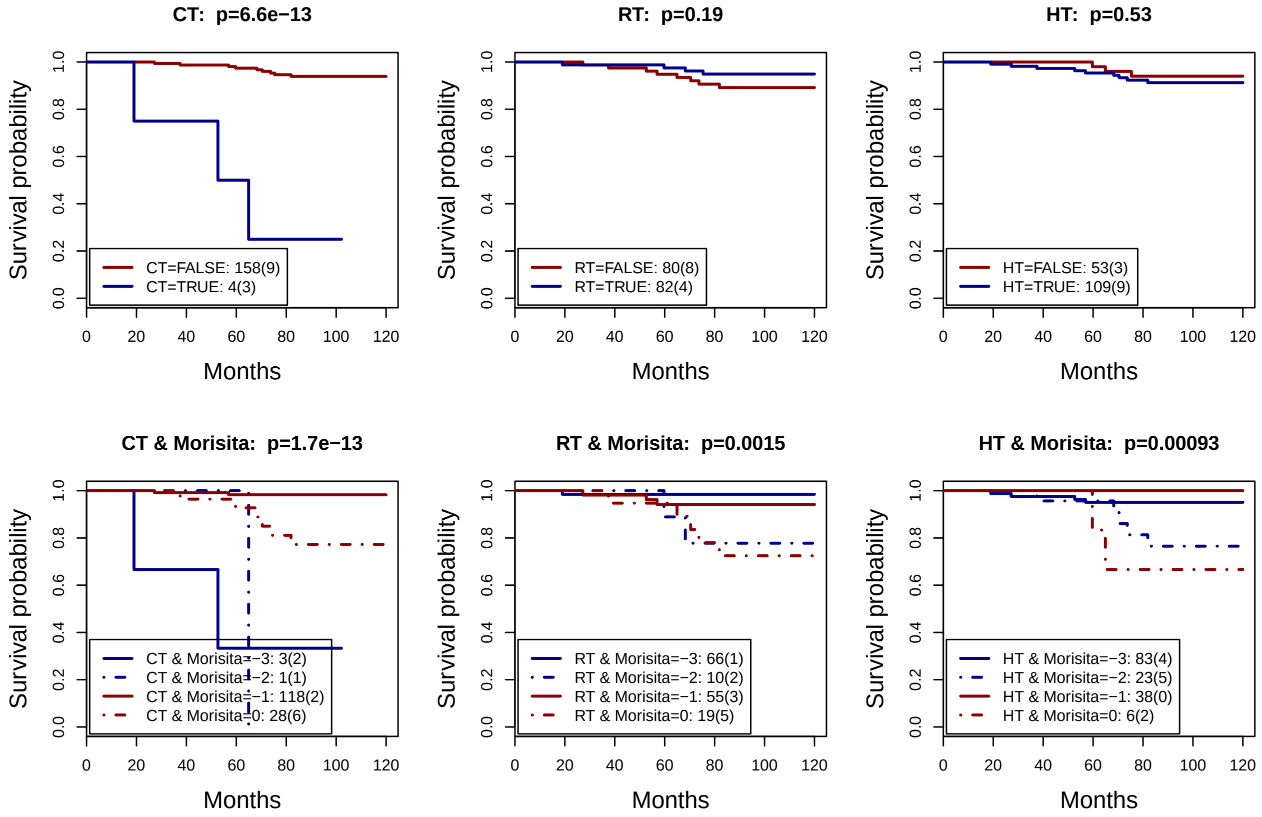


```
z <- DF[,4] > th_lumA
set2 <- grepl('LumA', trait$Pam50Subtype)  & Site[[2]]
ct <- trait$ct!='null'
rt <- trait$rt!='null'
ht <- trait$ht!='null'
par(mfrow=c(2,3))
plotSurv(trait$S_10year[set2], ct[set2], name='CT', col=cols, lty=ltys, mark.time=F)
plotSurv(trait$S_10year[set2], rt[set2], name='RT', col=cols, lty=ltys, mark.time=F)
plotSurv(trait$S_10year[set2], ht[set2], name='HT', col=cols, lty=ltys, mark.time=F)
plotSurv(trait$S_10year[set2], -1*(z[set2]) -2*( ct[set2]), name='CT & Morisita', col=cols2, l
plotSurv(trait$S_10year[set2], -1*(z[set2]) -2*( rt[set2]), name='RT & Morisita', col=cols2, l
plotSurv(trait$S_10year[set2], -1*(z[set2]) -2*( ht[set2]), name='HT & Morisita', col=cols2, l
```

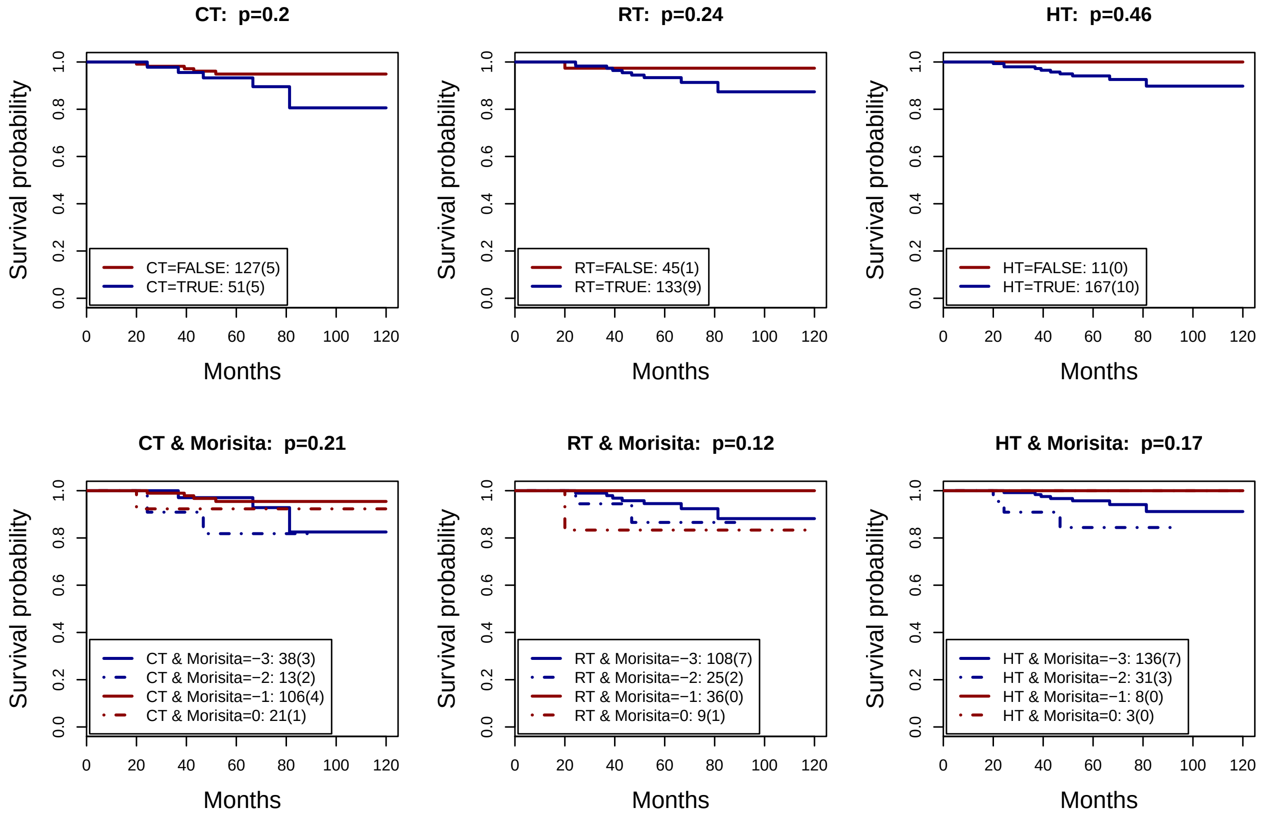

Morisita separated treatment groups and was significant in multivariate analysis considering all these variables:

```
set2 <- grepl('LumA', trait$Pam50Subtype)
summary(coxph(trait$S_10year~z+ct+rt+ht+trait$node+trait$grade+trait$size, subset=set2))

## Call:
## coxph(formula = trait$S_10year ~ z + ct + rt + ht + trait$node +
##     trait$grade + trait$size, subset = set2)
##
##   n= 315, number of events= 21
##    (32 observations deleted due to missingness)
##
##                 coef exp(coef) se(coef)      z Pr(>|z|)
## zTRUE        -1.4888    0.2256   0.4444 -3.350 0.000807 ***
## ctTRUE        0.9473    2.5787   0.5419  1.748 0.080482 .
## rtTRUE       -0.6960    0.4986   0.5262 -1.323 0.185946
## htTRUE       -0.2959    0.7439   0.8594 -0.344 0.730613
## trait$node    0.1290    1.1377   0.5013  0.257 0.796890
## trait$grade   0.6573    1.9296   0.3615  1.818 0.068993 .
## trait$size    0.9884    2.6869   0.5183  1.907 0.056543 .
## ---
## Signif. codes:
## 0 '***' 0.001 '**' 0.01 '*' 0.05 '.' 0.1 ' ' 1
##
##             exp(coef) exp(-coef) lower .95 upper .95
## zTRUE          0.2256     4.4318   0.09445    0.5391
## ctTRUE         2.5787     0.3878   0.89143    7.4593
## rtTRUE         0.4986     2.0057   0.17777    1.3984
## htTRUE         0.7439     1.3444   0.13802    4.0090
```

```
## trait$node      1.1377     0.8789    0.42589    3.0394
## trait$grade     1.9296     0.5182    0.95014    3.9189
## trait$size      2.6869     0.3722    0.97283    7.4212
##
## Concordance= 0.798  (se = 0.065 )
## Rsquare= 0.08   (max possible= 0.516 )
## Likelihood ratio test= 26.11  on 7 df,   p=0.000481
## Wald test            = 22.78  on 7 df,   p=0.00186
## Score (logrank) test = 29.45  on 7 df,   p=0.0001198
```

Morisita stratifies the LumA CT-null, RT-null and RT-treated, and HT-treated groups.

```
set2 <- grepl('LumA', trait$Pam50Subtype) & !ct
summary(coxph(trait$S_10year~z, subset=set2))[8:9]

## $conf.int
##       exp(coef) exp(-coef)  lower .95 upper .95
## zTRUE 0.1476914   6.770875 0.04961044 0.4396806
##
## $logtest
##        test          df       pvalue
## 10.79980190  1.00000000  0.00101511

set2 <- grepl('LumA', trait$Pam50Subtype) & rt
summary(coxph(trait$S_10year~z, subset=set2))[8:9]

## $conf.int
##       exp(coef) exp(-coef)  lower .95 upper .95
## zTRUE 0.2664473   3.753088 0.07978439 0.8898251
##
## $logtest
##       test          df      pvalue
## 3.82781095 1.00000000 0.05040873

set2 <- grepl('LumA', trait$Pam50Subtype) & !rt
summary(coxph(trait$S_10year~z, subset=set2))[8:9]

## $conf.int
##       exp(coef) exp(-coef)  lower .95 upper .95
## zTRUE   0.13666   7.317429 0.03416616 0.5466216
##
## $logtest
##        test          df       pvalue
## 8.453180123 1.000000000 0.003644057

set2 <- grepl('LumA', trait$Pam50Subtype) & ht
summary(coxph(trait$S_10year~z, subset=set2))[8:9]

## $conf.int
##       exp(coef) exp(-coef) lower .95 upper .95
## zTRUE 0.2570633   3.890092 0.1032945 0.6397392
##
## $logtest
##        test          df       pvalue
## 7.466953303 1.000000000 0.006284181
```

3.5 Robustness of Cox model in LumA

To test the robustness in Luminal A cancer, we estimate the univariate hazard ratio on DSS using a Cox proportional hazards model with both cohorts combined.

```
set2 <- grepl('LumA', trait$Pam50Subtype)
summary(coxph(trait$S_10year[set2] ~ z[set2]))

## Call:
## coxph(formula = trait$S_10year[set2] ~ z[set2])
##
##   n= 328, number of events= 21
##    (19 observations deleted due to missingness)
##
##                  coef exp(coef) se(coef)      z Pr(>|z|)
## z[set2]TRUE -1.6114    0.1996   0.4374 -3.684  0.00023 ***
## ---
## Signif. codes:
## 0 '***' 0.001 '**' 0.01 '*' 0.05 '.' 0.1 ' ' 1
##
##             exp(coef) exp(-coef) lower .95 upper .95
## z[set2]TRUE    0.1996       5.01   0.08469    0.4705
##
## Concordance= 0.646  (se = 0.041 )
## Rsquare= 0.036   (max possible= 0.504 )
## Likelihood ratio test= 12.05  on 1 df,   p=0.000517
## Wald test            = 13.57  on 1 df,   p=0.0002298
## Score (logrank) test = 16.76  on 1 df,   p=4.25e-05
```

We sampled different amount of data without replacement 1,000 times and repeated the log-rank survival analysis:

```
n <- sum(set2)
x1 <- NULL
for (m in seq(0.7, 0.95, by=0.05)){
        resB1 <- replicate(1000,1-pchisq(
               survdiff(trait$S_10year[set2] ~ z[set2],
                subset=sample(1:n, n*m, replace=FALSE)
               )$chisq,1))
        x1 <- c(x1, mean(resB1 < 0.05))
        }
```

This means in 100% our results of univariate analysis stay significant in the perturbed data.

```
x2 <- NULL
for (m in seq(0.7, 0.95, by=0.05)){
        resB2 <- replicate(1000, summary(coxph(trait$S_10year ~ z+trait$node+trait$size+trait$
            subset=which(set2)[sample(1:n, n*m, replace=FALSE)]))$coef[1,5])
        x2 <- c(x2, mean(resB2 < 0.05))
        }
```

```
matplot(cbind(x1, x2)*100, x=100*seq(0.7, 0.95, by=0.05), type='both', pch=19, col=c('dark blu
```

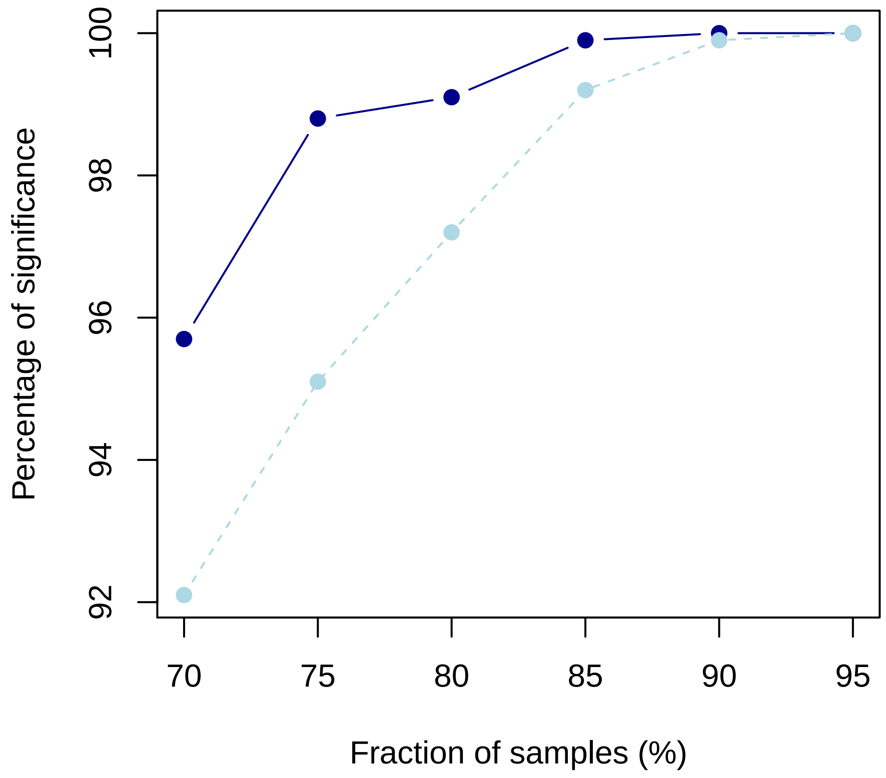

3.6 Morisita index in Her2+

We next focus on the Her2+ subtype determined using the SNP6 data. First the Morisita index was compared with standard clinical parameters including node, size and grade.

```
s <- 1
i <- 4
testrange=seq(0.2,.8,len=13)
p <- sapply(testrange, function(q){
  dat <- data.frame(x=DF[Site[[s]] & her2,i]>quantile(DF[Site[[s]] &her2,i], q, na.rm=T), S=tr
  fit <- survfit(S ~ x,data=dat)
  test <- survdiff(S ~ x, data=dat, rho=0)
  p.val <- 1 - pchisq(test$chisq, length(test$n) - 1)
  p.val})
```

Now plot the p-values from the log-rank test across different quantiles.

```
par(mfrow=c(1,3))
plot(testrange, -log(p), pch=19, xlab='Quantile range')
abline(h=-log(0.05), lty=2)
q <- testrange[which.min(p)]
abline(v=q)
th <- quantile(DF[Site[[s]] & her2,i], q, na.rm=T)
th_her2 <- th

for (j in 1:2){
```

```
  tmp <- replace.vector(DF[Site[[j]] & her2,i]>th_her2, c(TRUE, FALSE), c('High', 'Low'))
  try( plotSurv(trait$S_10year[Site[[j]] & her2,], tmp, fileType='', name=colnames(DF)[i]))
}
```

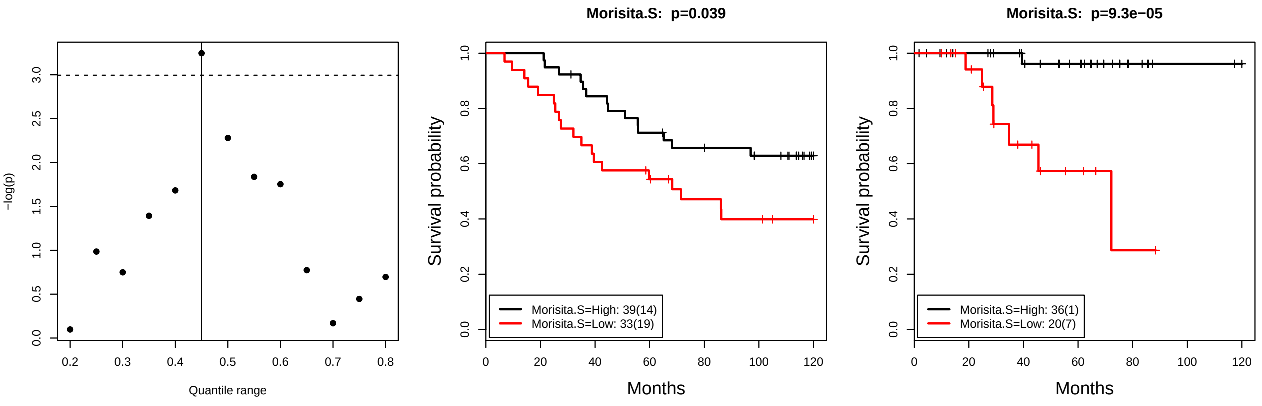


```
z <- DF[,4] > th_her2
```

The cutoff selected is 0.7106531 at 45 percentile.

3.7 Robustness of Cox model in Her2+

To test the robustness in Her2+ cancer, we estimate the univariate hazard ratio on DSS using a Cox proportional hazards model with both cohorts combined.

```
summary(coxph(trait$S_10year[her2] ~ z[her2]))

## Call:
## coxph(formula = trait$S_10year[her2] ~ z[her2])
##
##   n= 128, number of events= 41
##    (8 observations deleted due to missingness)
##
##                  coef exp(coef) se(coef)      z Pr(>|z|)
## z[her2]TRUE -1.1856    0.3056   0.3250 -3.647 0.000265 ***
## ---
## Signif. codes:
## 0 '***' 0.001 '**' 0.01 '*' 0.05 '.' 0.1 ' ' 1
##
##             exp(coef) exp(-coef) lower .95 upper .95
## z[her2]TRUE    0.3056      3.273    0.1616    0.5778
##
## Concordance= 0.654  (se = 0.039 )
## Rsquare= 0.103   (max possible= 0.942 )
## Likelihood ratio test= 13.98  on 1 df,   p=0.0001851
## Wald test            = 13.3  on 1 df,   p=0.0002649
## Score (logrank) test = 14.91  on 1 df,   p=0.0001128
```

We sampled different amount of data without replacement 1,000 times and repeated the log-rank survival analysis:

```
n <- sum(her2)
x1 <- NULL
for (m in seq(0.7, 0.95, by=0.05)){
        resB1 <- replicate(1000,1-pchisq(
               survdiff(trait$S_10year[her2] ~ z[her2],
                subset=sample(1:n, n*m, replace=FALSE)
               )$chisq,1))
        x1 <- c(x1, mean(resB1 < 0.05))
        }
```

This means in 100% our results of univariate analysis stay significant in the perturbed data.

```
x2 <- NULL
for (m in seq(0.7, 0.95, by=0.05)){
        resB2 <- replicate(1000, summary(coxph(trait$S_10year ~ z+trait$node+trait$size+trait$
            subset=which(her2)[sample(1:n, n*m, replace=FALSE)]))$coef[1,5])
        x2 <- c(x2, mean(resB2 < 0.05))
        }
```

```
matplot(cbind(x1, x2)*100, x=100*seq(0.7, 0.95, by=0.05), type='both', pch=19, col=c('dark blu
```

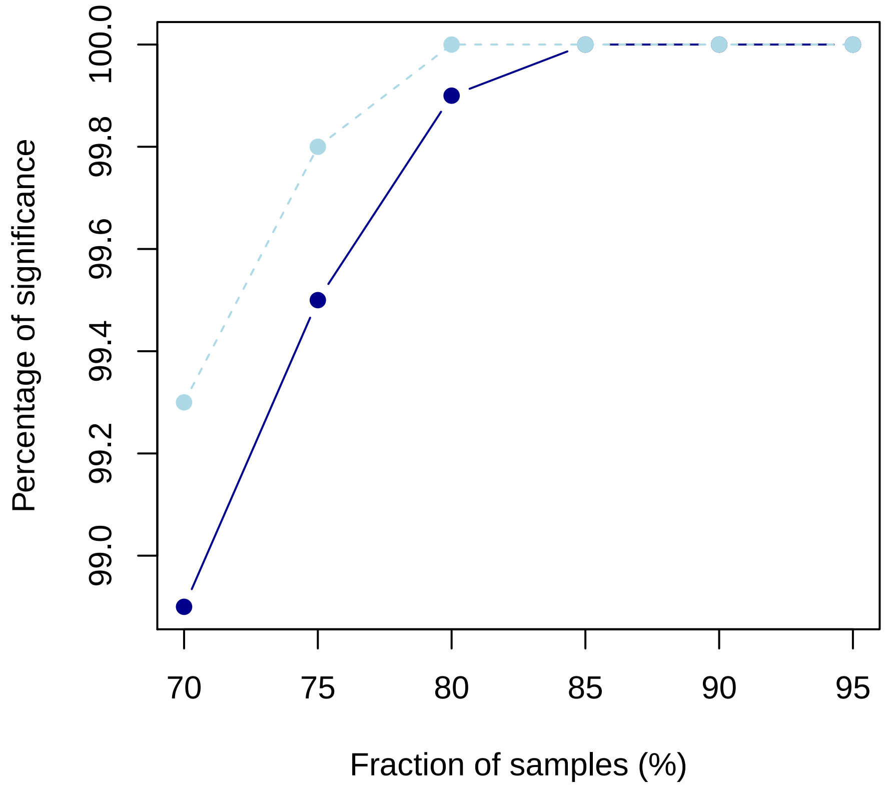

3.8 Morisita index and clinical parameters in Her2+

Node, size and grade:

```
set2 <- her2
cols <- c('darkred', 'darkblue')
ltys <- c(1,1,3,3)
cols2 <- c('darkblue', 'darkblue','darkred','darkred')
ltys2 <- c(1,3,1,3)
par(mfrow=c(2,3))
plotSurv(trait$S_10year[set2], trait$node[set2], name='Node', col=cols, lty=ltys, mark.time=F)
plotSurv(trait$S_10year[set2], trait$size[set2]==3, name='Size', col=cols, lty=ltys, mark.time
plotSurv(trait$S_10year[set2], trait$grade[set2]==3, name='Grade', col=cols, lty=ltys, mark.ti
plotSurv(trait$S_10year[set2], -1*(z[set2]) -2*(trait$node[set2]!=1), name='Node & Morisita',
plotSurv(trait$S_10year[set2], -1*(z[set2]) -2*(trait$size[set2]!=3), name='Size & Morisita',
plotSurv(trait$S_10year[set2], -1*(z[set2]) -2*(trait$grade[set2]!=3), name='Grade & Morisita'
```

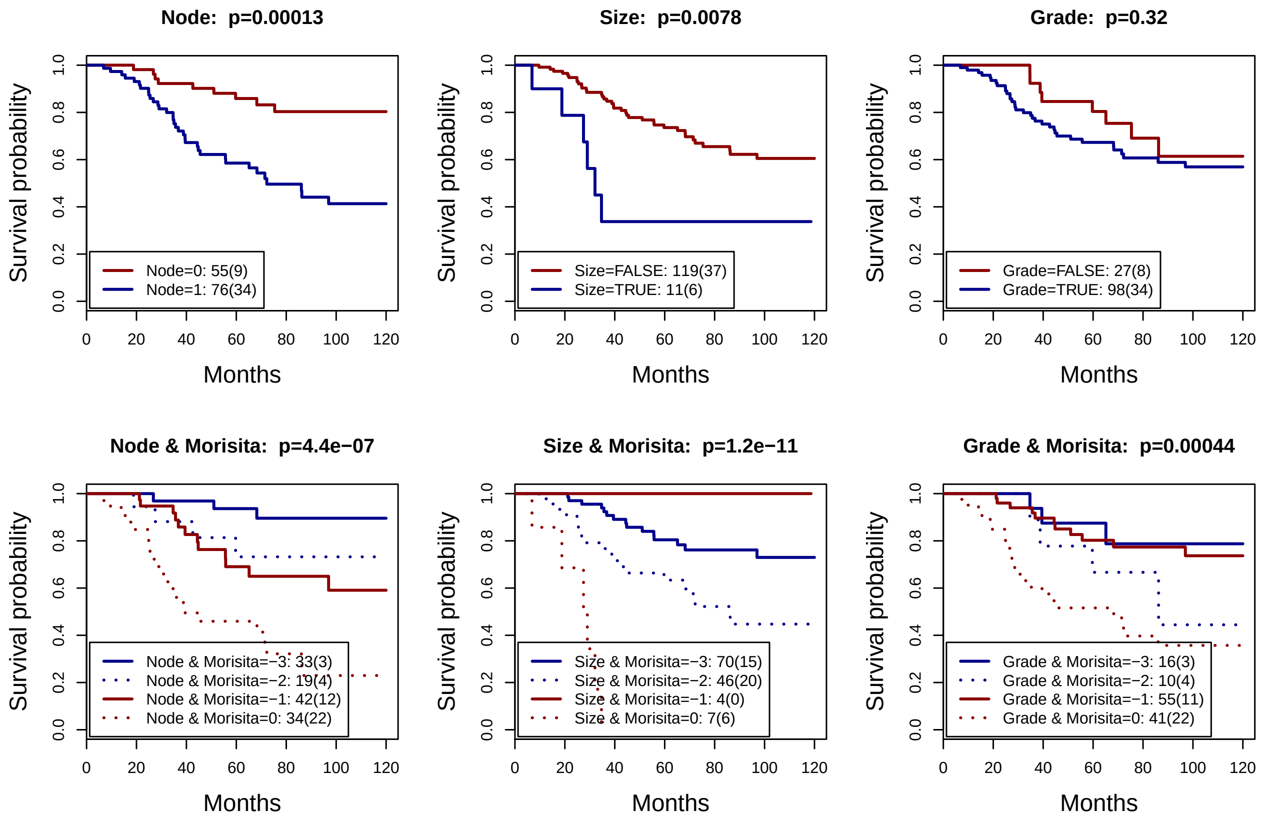

And other parameters for breast cancer including lymphocyte abundance, ER status and TP53 mutation:

```
par(mfrow=c(2,3))
plotSurv(trait$S_10year[set2], trait$lym[set2]<0.08, name='Lym', col=cols, lty=ltys, mark.time
plotSurv(trait$S_10year[set2], trait$ER.Expr[set2]=='-', name='ER', col=cols, lty=ltys, mark.t
plotSurv(trait$S_10year[set2], trait$TP53[set2]==0, name='TP53', col=cols, lty=ltys, mark.time
plotSurv(trait$S_10year[set2], -1*(z[set2]) + -2*(trait$lym[set2]>0.08), name='Lym & Morisita'
plotSurv(trait$S_10year[set2], -('+'==trait$ER.Expr[set2])*2- 1*( z[set2]), name='ER & Morisit
plotSurv(trait$S_10year[set2], -2*trait$TP53[set2] - 1*( z[set2]), name='TP53 & Morisita', col
```

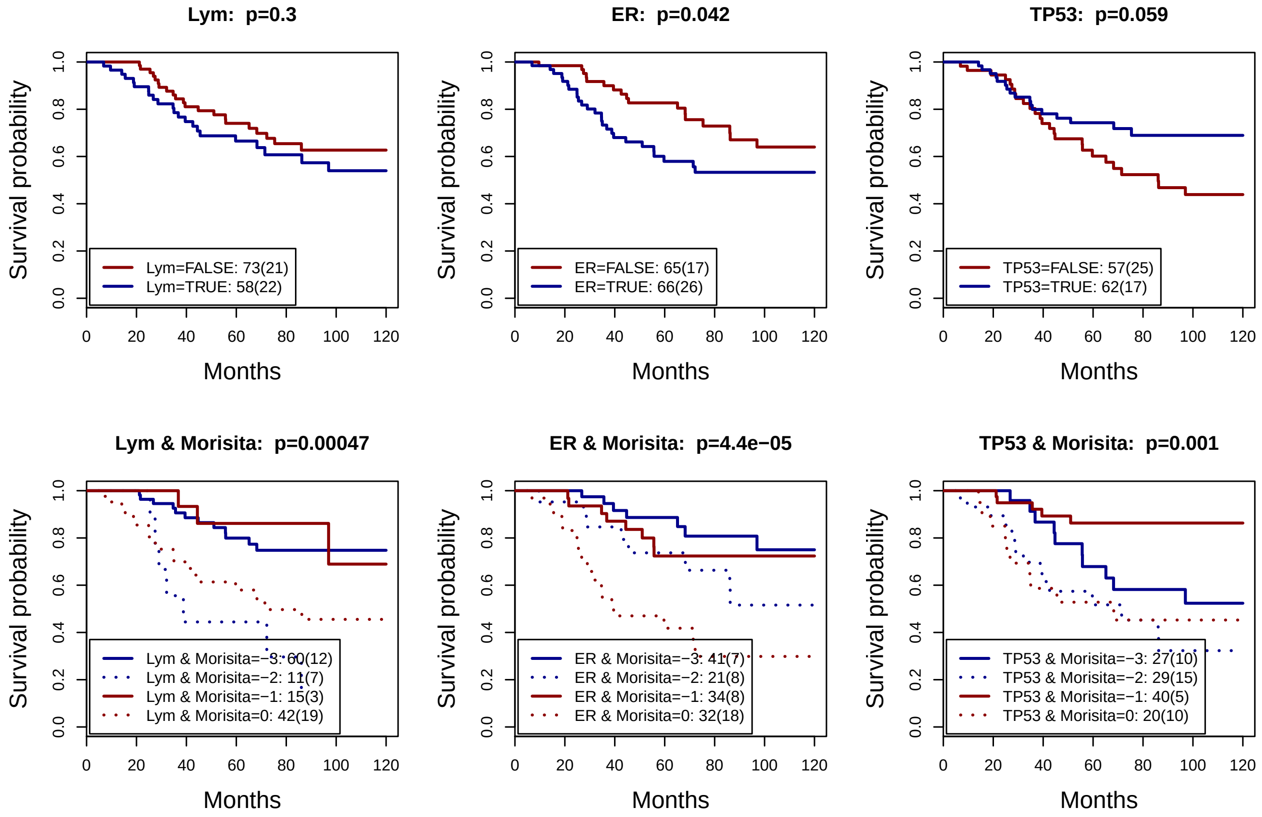

3.9 Morisita index and treatments in Her2+

Next, we looked at treatment options on the Her2+ subtype and whether their effects are different according to the Morisita index. Clinical data on chemotherapy (CT), radiotherapy (RT) and hormonal therapy (HT) are available.

```
set2 <- her2 & Site[[1]]
ct <- trait$ct!='null'
rt <- trait$rt!='null'
ht <- trait$ht!='null'
par(mfrow=c(2,3))
plotSurv(trait$S_10year[set2], ct[set2], name='CT', col=cols, lty=ltys, mark.time=F)
plotSurv(trait$S_10year[set2], rt[set2], name='RT', col=cols, lty=ltys, mark.time=F)
plotSurv(trait$S_10year[set2], ht[set2], name='HT', col=cols, lty=ltys, mark.time=F)
plotSurv(trait$S_10year[set2], -1*(z[set2]) -2*( ct[set2]), name='CT & Morisita', col=cols2, l
plotSurv(trait$S_10year[set2], -1*(z[set2]) -2*( rt[set2]), name='RT & Morisita', col=cols2, l
plotSurv(trait$S_10year[set2], -1*(z[set2]) -2*( ht[set2]), name='HT & Morisita', col=cols2, l
```

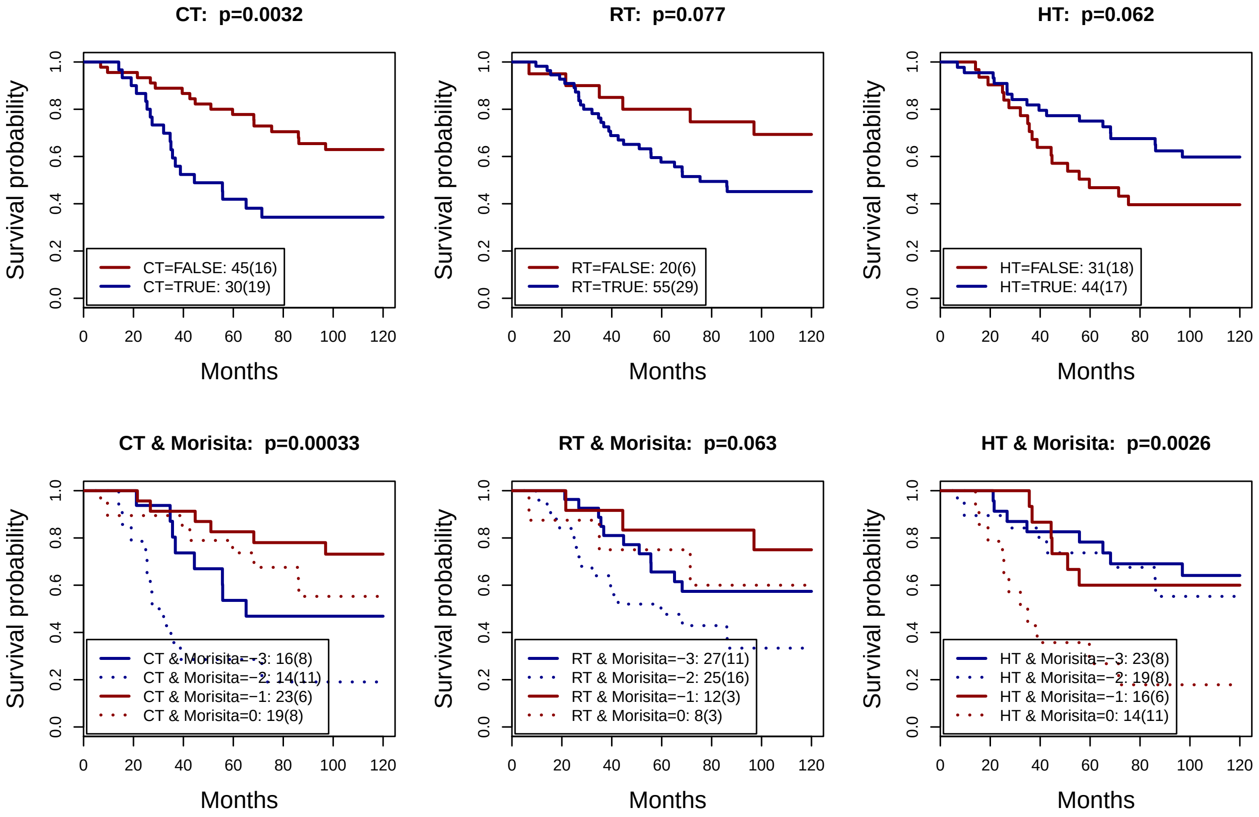


```
set2 <- her2 & Site[[2]]
ct <- trait$ct!='null'
rt <- trait$rt!='null'
ht <- trait$ht!='null'
par(mfrow=c(2,3))
plotSurv(trait$S_10year[set2], ct[set2], name='CT', col=cols, lty=ltys, mark.time=F)
plotSurv(trait$S_10year[set2], rt[set2], name='RT', col=cols, lty=ltys, mark.time=F)
plotSurv(trait$S_10year[set2], ht[set2], name='HT', col=cols, lty=ltys, mark.time=F)
plotSurv(trait$S_10year[set2], -1*(z[set2]) -2*( ct[set2]), name='CT & Morisita', col=cols2, l
plotSurv(trait$S_10year[set2], -1*(z[set2]) -2*( rt[set2]), name='RT & Morisita', col=cols2, l
plotSurv(trait$S_10year[set2], -1*(z[set2]) -2*( ht[set2]), name='HT & Morisita', col=cols2, l
```

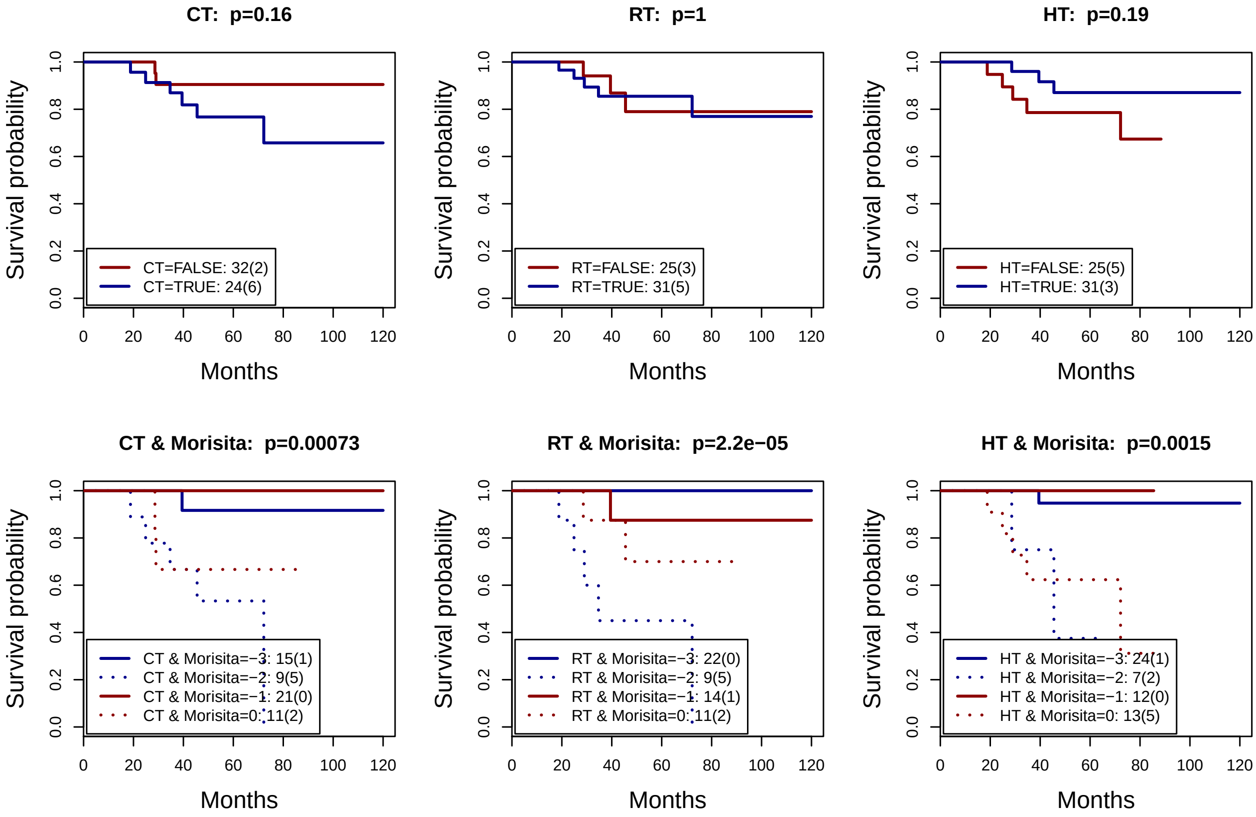

Examine within sites for these treatments:

```
par(mfrow=c(2,3))
set2 <- her2 & Site[[1]]
plotSurv(trait$S_10year[set2], -1*(z[set2]) -2*( ct[set2]), name='CT & Morisita', col=cols2, l
plotSurv(trait$S_10year[set2], -1*(z[set2]) -2*( rt[set2]), name='RT & Morisita', col=cols2, l
plotSurv(trait$S_10year[set2], -1*(z[set2]) -2*( ht[set2]), name='HT & Morisita', col=cols2, l
set2 <- her2 & Site[[2]]
plotSurv(trait$S_10year[set2], -1*(z[set2]) -2*( ct[set2]), name='CT & Morisita', col=cols2, l
plotSurv(trait$S_10year[set2], -1*(z[set2]) -2*( rt[set2]), name='RT & Morisita', col=cols2, l
plotSurv(trait$S_10year[set2], -1*(z[set2]) -2*( ht[set2]), name='HT & Morisita', col=cols2, l
```

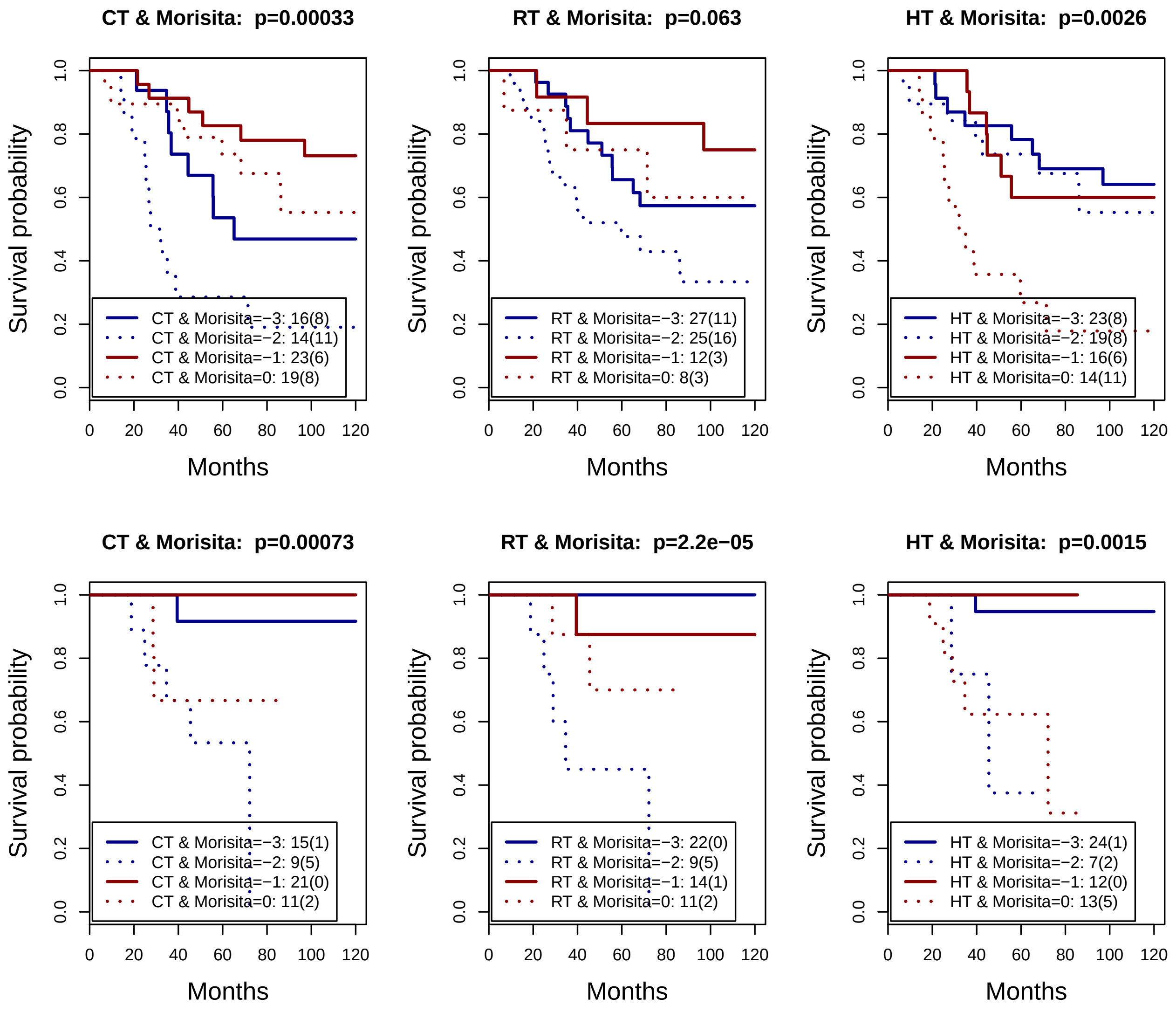

Difference in survival of CT-treated patients according to Morisita:

```
set3=trait$ct[set2]!='null'
summary(coxph(trait$S_10year[set2][set3]~z[set2][set3]))
```

```
## Call:
## coxph(formula = trait$S_10year[set2][set3] ~ z[set2][set3])
##
##   n= 24, number of events= 6
##    (1 observation deleted due to missingness)
##
##                         coef exp(coef) se(coef)     z
## z[set2][set3]TRUE -2.53377   0.07936  1.11625 -2.27
##                   Pr(>|z|)
## z[set2][set3]TRUE   0.0232 *
## ---
## Signif. codes:
## 0 '***' 0.001 '**' 0.01 '*' 0.05 '.' 0.1 ' ' 1
##
##                   exp(coef) exp(-coef) lower .95 upper .95
## z[set2][set3]TRUE   0.07936       12.6  0.008901    0.7075
##
## Concordance= 0.77  (se = 0.107 )
```

```
## Rsquare= 0.267   (max possible= 0.754 )
## Likelihood ratio test= 7.46  on 1 df,   p=0.006315
## Wald test            = 5.15  on 1 df,   p=0.02321
## Score (logrank) test = 8.09  on 1 df,   p=0.004461
```

Difference in survival of none CT-treated patients according to Morisita:

```
set3=trait$ct[set2]=='null'
summary(coxph(trait$S_10year[set2][set3]~z[set2][set3]))

## Call:
## coxph(formula = trait$S_10year[set2][set3] ~ z[set2][set3])
##
##   n= 32, number of events= 2
##
##                          coef  exp(coef)   se(coef)      z
## z[set2][set3]TRUE -2.184e+01  3.271e-10  2.358e+04 -0.001
##                   Pr(>|z|)
## z[set2][set3]TRUE    0.999
##
##                   exp(coef) exp(-coef) lower .95 upper .95
## z[set2][set3]TRUE 3.271e-10  3.057e+09         0       Inf
##
## Concordance= 0.885  (se = 0.165 )
## Rsquare= 0.152   (max possible= 0.314 )
## Likelihood ratio test= 5.28  on 1 df,   p=0.0216
## Wald test            = 0  on 1 df,   p=0.9993
## Score (logrank) test = 5.48  on 1 df,   p=0.01928
```

Three groups of multivariate Cox proportional hazard analysis of cancer-lymphocyte co-localisation computed by the Morisita index based on square tessellation in 136 Her2+ patients.

```
Write(writeCoxSummary(coxph(trait$S_10year~z+ct+rt+ht, subset = set2)), file='./data/morisita_
Write(writeCoxSummary(coxph(trait$S_10year~z+lym+trait$ER.Expr+trait$TP53, subset = set2)), fi
Write(writeCoxSummary(coxph(trait$S_10year~z+trait$grade+trait$node+trait$size, subset = set2)
```

```
table(trait$S_10year[set2 &ct,2], z[set2 & ct])

##
##     FALSE TRUE
##   0     4   14
##   1     5    1

table(trait$S_10year[set2 &!ct,2], z[set2 & !ct])

##
##     FALSE TRUE
##   0     9   21
##   1     2    0
```

Multivariate Cox analysis demonstrated the superior value of the Morisita index in predicting outcome of CT treatment.

```
Write(writeCoxSummary(coxph(trait$S_10year~z+trait$grade+trait$node+trait$size, subset = set2
Write(writeCoxSummary(coxph(trait$S_10year~z+lym+trait$ER.Expr+trait$TP53, subset = set2 & ct)
```

```
Write(writeCoxSummary(coxph(trait$S_10year~z+trait$grade+trait$node+trait$size, subset = set2
Write(writeCoxSummary(coxph(trait$S_10year~z+lym+trait$ER.Expr+trait$TP53, subset = set2 & rt)
```

```
Write(writeCoxSummary(coxph(trait$S_10year~z+trait$grade+trait$node+trait$size, subset = set2
Write(writeCoxSummary(coxph(trait$S_10year~z+lym+trait$ER.Expr+trait$TP53, subset = set2 & !ht
```

3.10 Compare with lymphocytic infiltration in Her2+

We first selected a optimal cutoff for lym using the discovery cohort of Her2+ samples.

```
set2 <- her2
s <- 1
i <- 4
testrange=seq(0.2,.8,len=13)
p <- sapply(testrange, function(q){
  dat <- data.frame(x=trait$lym[Site[[s]] & her2]>quantile(trait$lym[Site[[s]] &her2], q, na.r
  fit <- survfit(S ~ x,data=dat)
  test <- survdiff(S ~ x, data=dat, rho=0)
  p.val <- 1 - pchisq(test$chisq, length(test$n) - 1)
  p.val})
```

Now plot the p-values from the log-rank test across different quantiles.

```
par(mfrow=c(1,3))
plot(testrange, -log(p), pch=19, xlab='Quantile range')
abline(h=-log(0.05), lty=2)
q <- testrange[which.min(p)]
abline(v=q)
th <- quantile(trait$lym[Site[[s]] & her2], q, na.rm=T)
th_her2lym <- th

for (j in 1:2){
  tmp <- replace.vector(trait$lym[Site[[j]] & her2]>th_her2lym, c(TRUE, FALSE), c('High', 'Low
  try( plotSurv(trait$S_10year[Site[[j]] & her2,], tmp, fileType='', name='LymHer2'))
}
```

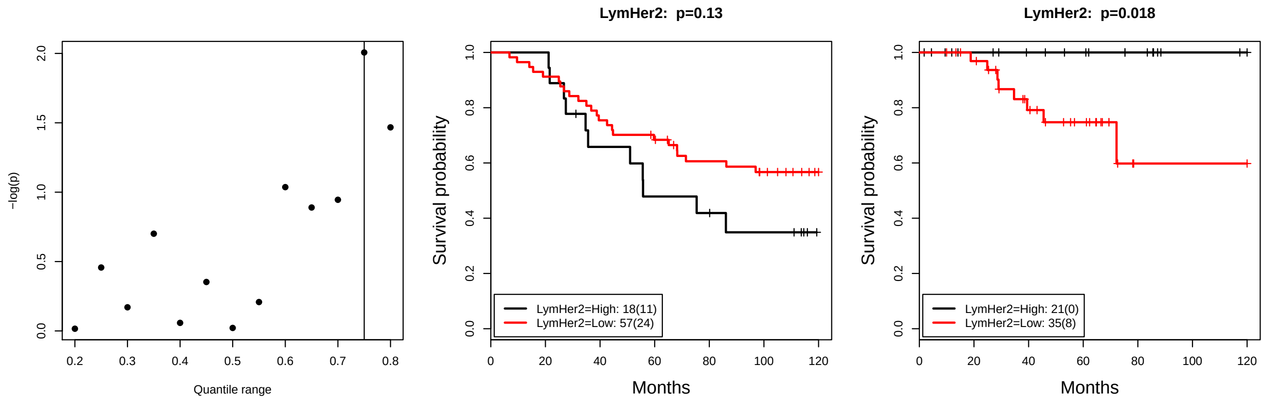

```r
z <- trait$lym > th_her2lym
```

```r
set2 <- her2
par(mfrow=c(2,3))
plotSurv(trait$S_10year[set2], trait$ct[set2]!='null', name='CT', col=cols, lty=ltys, mark.tim
plotSurv(trait$S_10year[set2], trait$rt[set2]!='null', name='RT', col=cols, lty=ltys, mark.tim
plotSurv(trait$S_10year[set2], trait$ht[set2]!='null', name='HT', col=cols, lty=ltys, mark.tim
plotSurv(trait$S_10year[set2], -1*(z[set2]) -2*(trait$ct[set2]=='null'), name='CT & Lym', col=
plotSurv(trait$S_10year[set2], -1*(z[set2]) -2*(trait$rt[set2]=='null'), name='RT & Lym', col=
plotSurv(trait$S_10year[set2], -1*(z[set2]) -2*(trait$ht[set2]=='null'), name='HT & Lym', col=
```

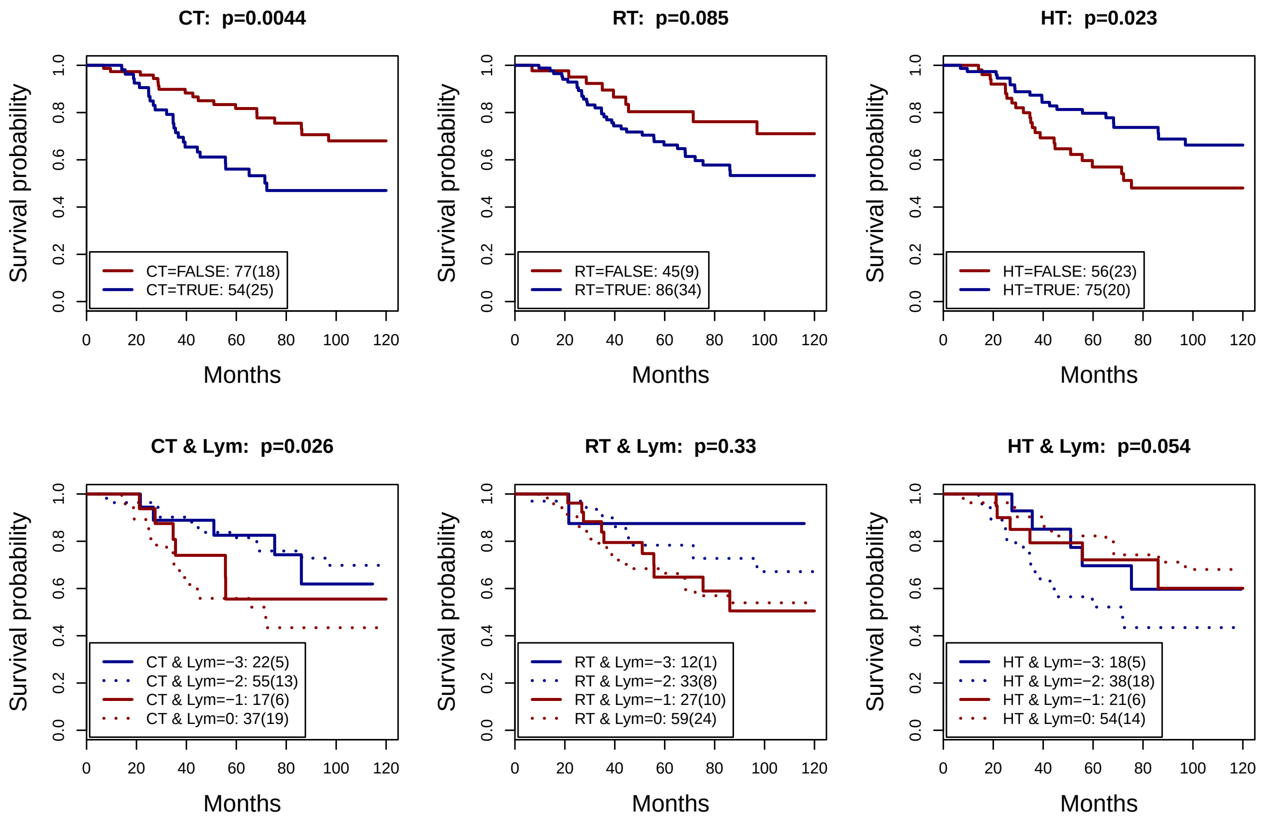

Then we tried the 8% cutoff for lym:

```r
z <- trait$lym > 0.08
par(mfrow=c(2,3))
plotSurv(trait$S_10year[set2], trait$ct[set2]!='null', name='CT', col=cols, lty=ltys, mark.tim
plotSurv(trait$S_10year[set2], trait$rt[set2]!='null', name='RT', col=cols, lty=ltys, mark.tim
plotSurv(trait$S_10year[set2], trait$ht[set2]!='null', name='HT', col=cols, lty=ltys, mark.tim
plotSurv(trait$S_10year[set2], -1*(z[set2]) -2*(trait$ct[set2]=='null'), name='CT & Lym', col=
plotSurv(trait$S_10year[set2], -1*(z[set2]) -2*(trait$rt[set2]=='null'), name='RT & Lym', col=
plotSurv(trait$S_10year[set2], -1*(z[set2]) -2*(trait$ht[set2]=='null'), name='HT & Lym', col=
```

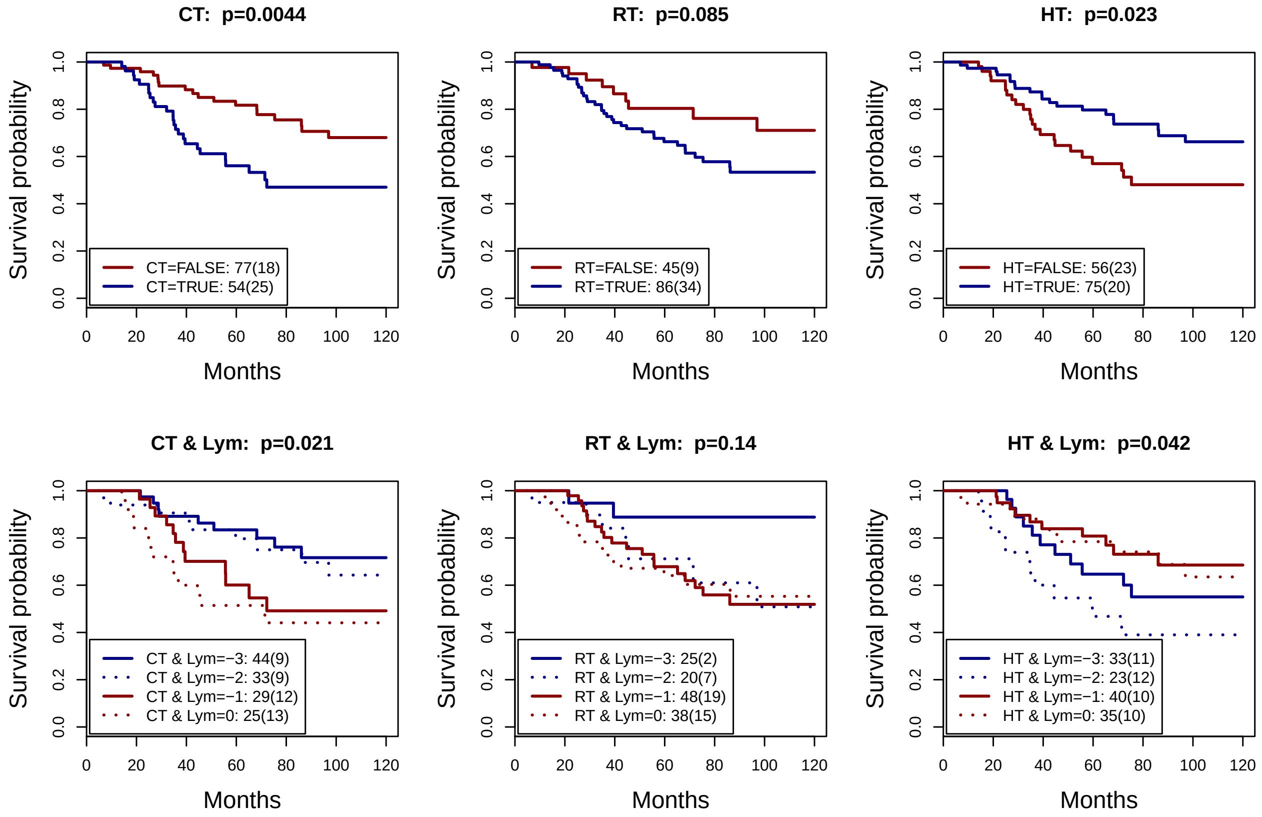

Neither of these analysis show that lym by itself has added value to treatment groups in Her2+ samples.

3.11 Lym interaction with Morisita in ER-

In ER- lymphocyte abundance is associated with prognosis but does not further stratify patient groups by the Morisita index. The optimal cutoff is 0.715724294551309 for ER-.

```
par(mfrow=c(1,3))
cols2 <- c('darkblue', 'darkblue','darkred','darkred')
ltys2 <- c(1,2,1,2)
z <- DF[,4] > 0.715724294551309
set2 <- "-"==trait$ER.Expr
plotSurv(trait$S_10year[set2], lym[set2], name='Lymphocyte abundance', col=c('darkred', 'darkb
plot(DF[set2,4], trait$lym[set2], pch=19, xlab='Morisita', ylab='Lymphocyte abundance', main=c
abline(v=0.715724294551309)
abline(h=0.08)
plotSurv(trait$S_10year[set2], -1*(z[set2]) -2*(lym[set2]), name='Lym & Morisita', col=cols2,
```

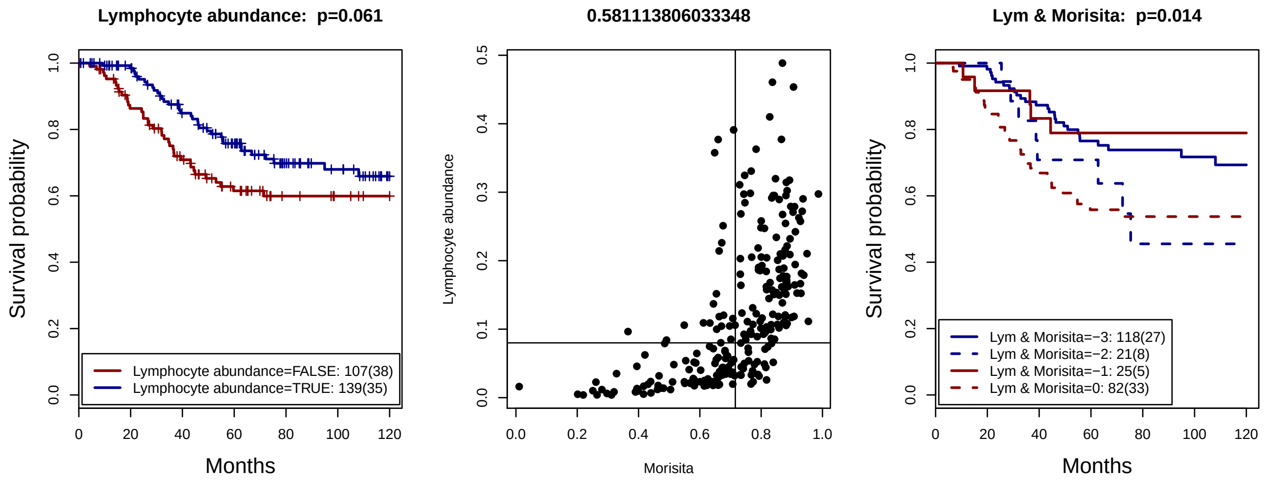


```
table(lym[set2], z[set2])
```

Here we test the differences in prognosis among these groups. First, groups with high morisita:

```
set3 <- z[set2]
survdiff(trait$S_10year[set2][set3]~lym[set2][set3])

## Call:
## survdiff(formula = trait$S_10year[set2][set3] ~ lym[set2][set3])
##
## n=143, 3 observations deleted due to missingness.
##
##                          N Observed Expected (O-E)^2/E
## lym[set2][set3]=FALSE  25        5     5.97    0.1564
## lym[set2][set3]=TRUE  118       27    26.03    0.0358
##                        (O-E)^2/V
## lym[set2][set3]=FALSE     0.192
## lym[set2][set3]=TRUE      0.192
##
##  Chisq= 0.2  on 1 degrees of freedom, p= 0.661
```

Then, groups with low morisita:

```
set3 <- !z[set2]
survdiff(trait$S_10year[set2][set3]~lym[set2][set3])

## Call:
## survdiff(formula = trait$S_10year[set2][set3] ~ lym[set2][set3])
##
## n=103, 2 observations deleted due to missingness.
##
##                         N Observed Expected (O-E)^2/E
## lym[set2][set3]=FALSE 82       33    32.08    0.0262
## lym[set2][set3]=TRUE  21        8     8.92    0.0941
##                        (O-E)^2/V
## lym[set2][set3]=FALSE      0.12
## lym[set2][set3]=TRUE       0.12
##
##  Chisq= 0.1  on 1 degrees of freedom, p= 0.729
```

Groups with high lym:

```
set3 <- lym[set2]
summary(coxph(trait$S_10year[set2][set3]~z[set2][set3]))

## Call:
## coxph(formula = trait$S_10year[set2][set3] ~ z[set2][set3])
##
##   n= 139, number of events= 35
##    (4 observations deleted due to missingness)
##
##                       coef exp(coef) se(coef)      z
## z[set2][set3]TRUE -0.6274    0.5340   0.4033 -1.556
##                   Pr(>|z|)
## z[set2][set3]TRUE     0.12
##
##                   exp(coef) exp(-coef) lower .95 upper .95
## z[set2][set3]TRUE     0.534      1.873    0.2422     1.177
##
## Concordance= 0.539  (se = 0.031 )
## Rsquare= 0.015   (max possible= 0.896 )
## Likelihood ratio test= 2.15  on 1 df,   p=0.1428
## Wald test            = 2.42  on 1 df,   p=0.1198
## Score (logrank) test = 2.5  on 1 df,   p=0.1138
```

Groups with low lym:

```
set3 <- !lym[set2]
summary(coxph(trait$S_10year[set2][set3]~z[set2][set3]))

## Call:
## coxph(formula = trait$S_10year[set2][set3] ~ z[set2][set3])
##
##   n= 107, number of events= 38
##    (1 observation deleted due to missingness)
##
##                       coef exp(coef) se(coef)      z
## z[set2][set3]TRUE -0.9044    0.4048   0.4805 -1.882
##                   Pr(>|z|)
## z[set2][set3]TRUE   0.0598 .
## ---
## Signif. codes:
## 0 '***' 0.001 '**' 0.01 '*' 0.05 '.' 0.1 ' ' 1
##
##                   exp(coef) exp(-coef) lower .95 upper .95
## z[set2][set3]TRUE    0.4048       2.47    0.1578     1.038
##
## Concordance= 0.567  (se = 0.037 )
## Rsquare= 0.04   (max possible= 0.954 )
## Likelihood ratio test= 4.38  on 1 df,   p=0.0363
## Wald test            = 3.54  on 1 df,   p=0.05979
## Score (logrank) test = 3.79  on 1 df,   p=0.05157
```

4 Generation of data step-by-step

We now go back to the process in which the DF measures were generated.

4.1 Image analysis data

CRImage processes a H&E slide by first dividing it into 2,000 pixels by 2,000 pixels sub-images and identifying cells in these sub-images. Therefore the cell locations for these sub-images need to be combined. We provide combined cell identifies and spatial locations for all 181 TNBC whole-section H&E sections as R data files in the 'CellPosAndMask' folder. These files are named by their image ID. Each file contain the x, y and class columns storing x y coordinates as well as the class of each cell in the large H&E slide. There is also a 'mask' binary matrix to denote the tissue area. The resolution of this image is 5um per pixel. Note that some sub images are missing in the output data if the processing failed due to large amount of artefacts or few tissue.

4.2 Voronoi tessellation

We use the getVoronoi function to generate polygon data for each tumour. It requires the cell position data in the 'CellPosAndMask' folder.

```
getVoronoi <- function(Dir, ff, Nr=NULL){
  library(sp)
  res <- try(load(paste(Dir,'/', ff, '.rdata', sep='')))
  if (class(res)!='try-error'){
    CellPos[,'class'] <- as.character(CellPos[,'class'])
    CellPos <- CellPos[(CellPos[,'class']!='a') & (!is.na(CellPos$x) & (!is.na(CellPos$y))), ]

    # divide slide into voronoi regions
    if (is.null(Nr))
      Nr <- sum(Mask)^(.5)/3 #how many regions to divide tumour into
    idx <- sample(which(CellPos$class=='c'), Nr)
    dat <- data.frame(x=CellPos$x[idx], y=CellPos$y[idx])
    xy <- SpatialPoints(cbind(CellPos$x, CellPos$y))
    v2 <- voronoipolygons2(dat)

    # which points belong to the polygons:pp
    pp <- vector('numeric', length=nrow(CellPos) )
    for (x in 1:length(v2@polygons))
        pp[!is.na(over(xy, SpatialPolygons(list(v2@polygons[[x]]))))] <- x

    save(Nr, v2, idx, pp, file=Paste(ff, '.rdata'))
}}
```

This needs to be performed for all tumours.

4.3 Square tessellation

We use the getSquare function to generate square polygon data for each tumour. It requires the cell position data in the 'CellPosAndMask' folder.

```
getSquare <- function(Dir, ff, s=50){
  require(sp)
  res <- try(load(paste(Dir,'/', ff, '.rdata', sep='')))
  if (class(res)!='try-error'){
    CellPos[,'class'] <- as.character(CellPos[,'class'])
```

```
    CellPos <- CellPos[(CellPos[,'class']!='a') & (!is.na(CellPos$x) & (!is.na(CellPos$y))), ]
    dat <- data.frame(x=CellPos$x[idx], y=CellPos$y[idx])
    xy <- SpatialPoints(cbind(CellPos$x, CellPos$y))
    x <- GridTopology(c(s/2,s/2), c(s,s), c(nrow(Mask)/s,ncol(Mask)/s))
    polys <- as(x, "SpatialPolygons")
    pp <- vector('numeric', length=nrow(CellPos) )
    for (x in 1:length(polys@polygons))
      pp[!is.na(over(xy, SpatialPolygons(list(polys@polygons[[x]]))))] <- x
    save(polys, pp, file=Paste(ff, '.rdata'))
  }
}
```

4.4 Computing correlation and Morisita index

Here we compute colocalisation with correlation and the Morisita index.

```
library(sp)
library(vegan)
library(spaa)
DF <- NULL
FF <- NULL

for (ff in trait$file){
 res <- try(load(Paste(ff, '.rdata')))
 if (class(res)!='try-error'){
   res <- load(paste(Dir,'/', ff, '.rdata', sep=''))
   CellPos[,'class'] <- as.character(CellPos[,'class'])
   CellPos <- CellPos[(CellPos[,'class']!='a') & (!is.na(CellPos$x) & (!is.na(CellPos$y))), ]
   dat <- data.frame(x=CellPos$x[idx], y=CellPos$y[idx])

   xy <- SpatialPoints(cbind(CellPos$x, CellPos$y))
   df <-  sapply(1:length(v2@polygons), function(x) sum(pp==x))
   df3 <- sapply(c('c', 'l', 'o'), function(y)
              sapply(1:length(v2@polygons), function(x) sum(pp==x & CellPos$class==y)))
   polys <- SpatialPolygons(v2@polygons)
   Area <- sapply(polys@polygons, function(x) x@area)
   Ne  <- sapply(ne, function(x) median(x$entropy[x$cellType=='c'], na.rm=T))
   SD  <- sapply(ne, function(x) sd(x$entropy[x$cellType=='c'], na.rm=T))
   df3central <- df3[df/Area*10 > 0.2,]
   FF <- c(FF, ff)
   DF <- rbind(DF, c(cor(df3central)[2],
                  niche.overlap(df3central[,1:2], "morisita")
                  ))
  }  }
rownames(DF) <- FF
DF <- DF[match(trait$file, FF),]
rownames(DF) <- trait$file
save(DF, file='./data/DF.rdata')
```

This generates data for Voronoi or square tessellation.

5 Session Info

This document was prepared using R package knitr. Funciton knit2pdf("sweave.rnw") was used to compile the sweave file and generate the pdf file.

```
sessionInfo()

## R version 3.1.1 (2014-07-10)
## Platform: x86_64-apple-darwin13.1.0 (64-bit)
##
## locale:
## [1] en_GB.UTF-8/en_GB.UTF-8/en_GB.UTF-8/C/en_GB.UTF-8/en_GB.UTF-8
##
## attached base packages:
## [1] parallel  splines   stats     graphics  grDevices
## [6] utils     datasets  methods   base
##
## other attached packages:
##  [1] knitr_1.8            SAGx_1.38.0
##  [3] multtest_2.20.0      annotate_1.42.1
##  [5] org.Hs.eg.db_2.14.0  RSQLite_1.0.0
##  [7] DBI_0.3.1            AnnotationDbi_1.26.1
##  [9] GenomeInfoDb_1.0.2   Biobase_2.24.0
## [11] BiocGenerics_0.10.0  biomaRt_2.20.0
## [13] survival_2.37-7      BiocInstaller_1.14.3
## [15] fdrtool_1.2.13
##
## loaded via a namespace (and not attached):
##  [1] bitops_1.0-6    digest_0.6.7    evaluate_0.5.5
##  [4] formatR_1.0     highr_0.4       IRanges_1.22.10
##  [7] MASS_7.3-33     RCurl_1.95-4.5  stats4_3.1.1
## [10] stringr_0.6.2   tcltk_3.1.1     tools_3.1.1
## [13] XML_3.98-1.1    xtable_1.7-4
```
